# Bimodular architecture of bacterial effector SAP05 that drives ubiquitin-independent targeted protein degradation

**DOI:** 10.1073/pnas.2310664120

**Published:** 2023-12-01

**Authors:** Qun Liu, Abbas Maqbool, Federico G. Mirkin, Yeshveer Singh, Clare E. M. Stevenson, David M. Lawson, Sophien Kamoun, Weijie Huang, Saskia A. Hogenhout

**Affiliations:** ^a^Department of Crop Genetics, John Innes Centre, Norwich Research Park, Norwich NR4 7UH, United Kingdom; ^b^Department of Biochemistry and Metabolism, John Innes Centre, Norwich Research Park, Norwich NR4 7UH, United Kingdom; ^c^The Sainsbury Laboratory, University of East Anglia, Norwich NR4 7UH, United Kingdom; ^d^National Key Laboratory of Plant Molecular Genetics, Shanghai Centre for Plant Stress Biology, Centre for Excellence in Molecular Plant Sciences, Chinese Academy of Sciences, Shanghai 20032, China

**Keywords:** targeted protein degradation, ubiquitin-independent, bacterial effector protein, 26S proteasome, phytoplasma

## Abstract

In eukaryotes, targeted protein degradation (TPD) typically depends on a series of interactions among ubiquitin ligases that transfer ubiquitin molecules to substrates leading to degradation by the 26S proteasome. We previously identified that the bacterial effector protein SAP05 mediates ubiquitin-independent TPD. SAP05 forms a ternary complex via interactions with the von Willebrand Factor Type A (vWA) domain of the proteasomal ubiquitin receptor Rpn10 and the zinc-finger (ZnF) domains of the SQUAMOSA-PROMOTER BINDING PROTEIN-LIKE (SPL) and GATA BINDING FACTOR (GATA) transcription factors (TFs). This leads to direct TPD of the TFs by the 26S proteasome. Here, we report the crystal structures of the SAP05–Rpn10^vWA^ complex at 2.17 Å resolution and of the SAP05–SPL5^ZnF^ complex at 2.20 Å resolution. Structural analyses revealed that SAP05 displays a remarkable bimodular architecture with two distinct nonoverlapping surfaces, a “loop surface” with three protruding loops that form electrostatic interactions with ZnF, and a “sheet surface” featuring two β-sheets, loops, and α-helices that establish polar interactions with vWA. SAP05 binding to ZnF TFs involves single amino acids responsible for multiple contacts, while SAP05 binding to vWA is more stable due to the necessity of multiple mutations to break the interaction. In addition, positioning of the SAP05 complex on the 26S proteasome points to a mechanism of protein degradation. Collectively, our findings demonstrate how a small bacterial bimodular protein can bypass the canonical ubiquitin–proteasome proteolysis pathway, enabling ubiquitin-independent TPD in eukaryotic cells. This knowledge holds significant potential for the creation of TPD technologies.

## Significance

This study reveals the structure–function relationships and mechanisms of SAP05 bacterial effectors in a unique protein degradation process. Crystal structures demonstrate that SAP05 acts as a scaffold, facilitating efficient substrate degradation by bringing together the substrate and the 26S proteasome receptor Rpn10. Substrate degradation triggered by direct interaction of SAP05 and a specific 26S proteasome component is independent of ubiquitination, distinct from PROteolysis-TArgeting Chimeras (PROTAC) and other similar systems. This finding can guide the design of SAP05-like molecules for a protein degradation technology with significant potential in biotechnology and biomedicine. It also emphasizes the capacity of proteasome subunits to initiate protein degradation, promoting the advancement of alternative approaches that bypass ubiquitination.

Pathogenic bacteria secrete effector proteins that manipulate host cell processes, aiding in bacterial survival and often resulting in disease (1). These effector proteins are a source of biochemical innovation and include some of the most remarkable proteins known to function inside host cells. Notable examples include Transcription Activator-Like (TAL) effectors, derived from plant pathogenic bacteria, which can be engineered to bind specific DNA sequences (2), type-III effector proteins from animal parasitic bacteria, capable of re-engineering kinase pathways (3), and the CRISPR/Cas systems—immune defenses against bacteriophages, now harnessed as groundbreaking genetic manipulation tools (4). In addition, we recently identified that SAP05 effectors of bacterial phytoplasma pathogens enable the degradation of structured proteins by the 26S proteasome in a ubiquitin-independent manner (5). Similar to other bacterial effectors, the utility of SAP05 proteins can extend beyond the realm of natural biology, as they have the potential to serve as tools in biotechnology and biomedicine. However, unraveling the structure–function relationships and underlying mechanisms of SAP05 bacterial effectors is necessary to pave the way for their applications in synthetic biology.

Efficient and selective protein degradation is crucial for all living organisms (6, 7). A significant portion of energy in cells is devoted to the process of protein degradation. This expenditure of energy is vital for cells to adapt and react to their surrounding environment (8–13). Protein degradation is executed by a diverse family of enzymes, proteases, that hydrolyse peptide bonds (6). To prevent the destruction of proteins not destined for degradation and avoid the accidental disruption of the cellular proteome, protein degradation must be spatially and temporally controlled (14–16).

The 26S proteasome is such a self-compartmentalizing device. It is a highly sophisticated complex with distinct proteolytic activities able to degrade proteins selectively and efficiently in all eukaryotes (14, 17). With 33 different subunits and approximately 2.5 MDa in mass, it is the largest ATP-dependent protease machinery in the cell (16, 18, 19). The subunits are organized in two particles: the catalytic 20S core particle (CP) and the 19S regulatory particle (RP). The 20S CP is a cylindrical complex containing four heptameric rings with multiple catalytic β subunits and can degrade intrinsically disordered proteins by itself, including proteins that are unfolded by damage or mutations (20, 21). The 19S regulatory particle (RP) sits on one or both sides of the CP and is primarily required for the degradation of structured proteins via mechanically translocating substrates into the degradation chamber of the CP.

The degradation of structured proteins in eukaryotic organisms is predominantly regulated by the ubiquitin–proteasome system (UPS) (22, 23). UPS involves prior decoration of substrates with ubiquitin chains via the complex and consecutive actions of E1, E2, and E3 ligase enzymes (20, 24–27). The ubiquitin chains are recognized by 19S RP substrate receptors regulatory particle non-ATPase (Rpn) 1, Rpn10, and Rpn13, which enable substrate degradation by translocating stretched unstructured regions into the 20S CP channel (7, 17). Efficient degradation is also dependent on Rpn11 that removes the ubiquitin units during substrate translocation (8, 15). Beyond binding ubiquitinated substrates, the multimodular Rpn1, Rpn10, and Rpn13 enable transient interactions with ubiquitin-binding shuttle factors, such as RADIATION-SENSITIVE PROTEIN 23 (RAD23), and ubiquitin processing enzymes (7, 12).

A number of bacterial effector proteins target or co-opt the UPS. Among these, SAP05 effectors of bacterial phytoplasma pathogens enable the degradation of structured proteins by the 26S proteasome in a ubiquitin-independent manner (5). SAP05 binds both the von Willibrand factor type A (vWA) domain of Rpn10 and the zinc-finger (ZnF) domain of multiple members of two distinct plant transcription factor families, known as SQUAMOSA-PROMOTER BINDING PROTEIN-LIKE (SPL) and GATA BINDING FACTORS (GATAs) (5). By hijacking Rpn10, SAP05 mediates the degradation of these TFs, leading to dramatic changes in plant development that includes leaf and stem proliferations, neoteny and increased longevity (5), as well as increased plant colonization of phytoplasma insect vectors (28). Other phytoplasma effectors, known as SAP54/phyllogens, were found to hijack the 26S proteasome shuttle factor RAD23 to mediate the degradation of plant MCM1, AGAMOUS, DEFICIENS, and SRF (MADS) domain transcription factors leading to the induction of leaf-like flowers and other changes in flower development (29), and this also occurs in a ubiquitin-independent manner (30). Therefore, phytoplasma effectors appear to directly target ubiquitin receptors or shuttle factors to mediate ubiquitin-independent protein degradation. However, the precise biochemical mechanisms by which these small effector proteins form connections between unrelated proteins to mediate targeted protein degradation in a ubiquitin-independent manner remain uncharacterized.

Here, we determined how SAP05 mediates ubiquitin-independent targeted protein degradation by generating crystal structures of SAP05 in complex with the ZnF domain of SPL5 (SPL5^ZnF^) and with the vWA domain of Rpn10 (Rpn10^vWA^) and validating the ensuing mechanistic model using mutagenesis and protein degradation experiments. We found that SAP05 acts like a scaffold by bridging ZnF and vWA on opposing surfaces at 1:1 stoichiometries. Furthermore, SAP05 binding to vWA does not appear to cause steric hindrance with other 26S proteasome components and their interactors. Our data show how the bacterial SAP05 effector has evolved as a functional adapter of the 26S proteasome to bypass the canonical UPS cellular proteolysis pathway and enable ubiquitin-independent degradation of structured eukaryotic proteins.

## Results

### Crystal Structures of the SAP05–SPL5^ZnF^ and SAP05–Rpn10^vWA^ Complexes Reveal Two Distinct Binding Faces of SAP05.

We previously demonstrated that SAP05 of Aster Yellows phytoplasma strain Witches Broom (AYWB) forms a ternary complex with the ZnF domain of SPL5 (SPL5^ZnF^) and vWA domain of Rpn10 (Rpn10^vWA^) (5). To investigate how SAP05 binds these two larger proteins, we determined crystal structures of SAP05 in complex with SPL5^ZnF^ and with Rpn10^vWA^. We expressed constructs containing SAP05 residues 33 to 135 that correspond to the entire mature part of SAP05 (without the first 32 amino acids encoding the signal peptide that is cleaved off during secretion of the effector), residues 60 to 127 of *Arabidopsis thaliana* SPL5 (accession number AT3G15270) corresponding to the ZnF domain and residues 2 to 193 comprising the vWA domain of *A. thaliana* Rpn10 (accession number AT4G38630) (*SI Appendix*, Fig. S1*A*) in *Escherichia coli*. SAP05 and SPL5^ZnF^ were individually expressed and successfully purified at high purity with immobilized metal-affinity chromatography (IMAC) via 6× His tags followed by tag removal and gel filtration (Superdex 75 26/60) (*SI Appendix*, Fig. S1*B*). As Rpn10^vWA^ formed aggregates upon purification in the absence of SAP05, Rpn10^vWA^ was coexpressed with SAP05 and purified as a complex using the same general method (*SI Appendix*, Fig. S1*B*). To generate the SAP05–SPL5^ZnF^ complex, we mixed equimolar amounts of purified SAP05 and SPL5^ZnF^.

We obtained crystals for both complexes, which yielded X-ray data to 2.20 Å resolution for SAP05–SPL5^ZnF^ and to 2.17 Å resolution for SAP05–Rpn10^vWA^. The structure of the SAP05–SPL5^ZnF^ complex was solved via the single-wavelength anomalous diffraction method due to the presence of Zn^2+^ ions bound to ZnF, and that of SAP05–Rpn10^vWA^ was solved with the molecular replacement method using a copy of SAP05 from the SAP05–SPL5^ZnF^ structure and a homology model for Rpn10^vWA^ as templates. The details of X-ray data processing and structure solution are described in *Materials and Methods*. The X-ray data collection, refinement, and validation statistics are shown in Table 1.

**Table 1.** X-ray data collection, processing, and refinement statistics

Dataset	SAP05–SPL5^ZnF^ SAD phasing	SAP05–SPL5^ZnF^ refinement	SAP05–Rpn10^vWA^
Data collection			
Diamond light source beamline	I04	I04	I04
Wavelength (Å)	1.2770	0.9795	0.9796
Detector	Eiger2 XE 16 M	Eiger2 XE 16 M	Eiger2 XE 16 M
Resolution range (Å)	70.27–2.90 (3.08–2.90)	82.51–2.20 (2.24–2.20)	68.60–2.17 (2.24–2.17)
Space group	*P*2_1_	*P*2_1_	*P*2_1_
Cell parameters (Å/°)	*a* = 78.87, *b* = 165.33, *c* = 80.99, *β* = 109.62	*a* = 78.69, *b* = 165.02, *c* = 80.86, *β* = 109.65	*a* = 42.39, *b* = 68.60, *c* = 49.85, *β* = 92.78
Total no. of measured intensities	507,669 (18,366)	1,386,043 (69,812)	210,355 (18,117)
Unique reflections	75,200 (3,838)	98,262 (4,890)	15,199 (1,337)
Multiplicity	6.8 (4.8)	14.1 (14.3)	13.8 (13.6)
Mean *I*/σ(*I*)	9.2 (1.1)	10.4 (1.2)	10.9 (1.0)
Completeness (%)	98.7 (84.4)	100.0 (100.0)	100.0 (99.9)
*R*_merge_ ^*^	0.104 (1.004)	0.129 (2.029)	0.118 (2.599)
*R*_meas_ ^†^	0.112 (1.126)	0.134 (2.182)	0.122 (2.700)
*CC*_½_ ^‡^	0.998 (0.797)	0.999 (0.791)	0.999 (0.436)
Wilson *B* value (Å^2^)	53.5	47.5	52.7
Refinement			
Resolution range (Å)	–	74.21–2.20 (2.26–2.20)	49.84–2.17 (2.23–2.17)
Reflections: working/free^§^	–	93,364/4,855	14,486/687
*R*_work_ ^¶^	–	0.223 (0.402)	0.198 (0.314)
*R*_free_ ^¶^	–	0.251 (0.394)	0.252 (0.342)
Ramachandran plot: favored/allowed/disallowed (%)	–	99.0/1.0/0.0	99.0/4.0/0.0
R.m.s. bond distance deviation (Å)	–	0.008	0.007
R.m.s. bond angle deviation (°)	–	1.51	1.44
Mean *B* factor (Å^2^)	–	62.6	59.8
PDB accession code	–	8PFC	8PFD

^*^$R_{\text{merge}}\;=\;\Sigma_{hkl}\;\Sigma_{i}\left|I_{i}\left(hkl\right)-\langle I\left(hkl\right)\rangle \right|/\Sigma_{hkl}\;\Sigma_{i}I_{i}\left(hkl\right)$.

^†^$R_{\text{meas}}=\;\Sigma_{hkl}{\left[N/\left(N-1\right)\right]}^{1/2}\times \Sigma_{i}\left|I_{i}\left(hkl\right)-\langle I\left(hkl\right)\rangle \right|/\Sigma_{hkl}\Sigma_{i}I_{i}\left(hkl\right)$   , where $I_{i}\left(hkl\right)$   is the *i*th observation of reflection *hkl*, $\langle I\left(hkl\right)\rangle$   is the weighted average intensity for all observations *i* of reflection *hkl*, and *N* is the number of observations of reflection *hkl*.

^‡^*CC*_½_ is the correlation coefficient between symmetry equivalent intensities from random halves of the dataset.

^§^The dataset was split into “working” and “free” sets consisting of 95 and 5% of the data, respectively. The free set was not used for refinement.

^¶^The R-factors *R*_work_ and *R*_free_ are calculated as follows: $R=\Sigma \left(\left|F_{\mathrm{obs}}-F_{\mathrm{calc}}\right|\right)/\Sigma \left|F_{\mathrm{obs}}\right|$ , where $F_{obs}$ and $F_{calc}$ are the observed and calculated structure factor amplitudes, respectively.

Values in parentheses are for the outer resolution shell.

The structure of SPL5^ZnF^ resembles the previously determined NMR structures of ZnF domain of *A. thaliana* SPL4 (PDB 1UL4; rmsd = 1.56 Å) and SPL7 (PDB 1UL5; rmsd = 1.76 Å) (31). As well, our Rpn10^vWA^ structure is similar to that of vWA in the spinach 26S proteasome Cryo-EM structure (PDB 8AMZ; rmsd = 0.91 Å) (32). However, a search for SAP05 structure homologues using DALI sever (http://ekhidna2.biocenter.helsinki.fi/dali/) (33) and PDBeFold server (https://www.ebi.ac.uk/msd-srv/ssm/) (34) failed to identify any publicly available, experimentally determined structures with significant structural similarity to SAP05.

**Fig. 1.**
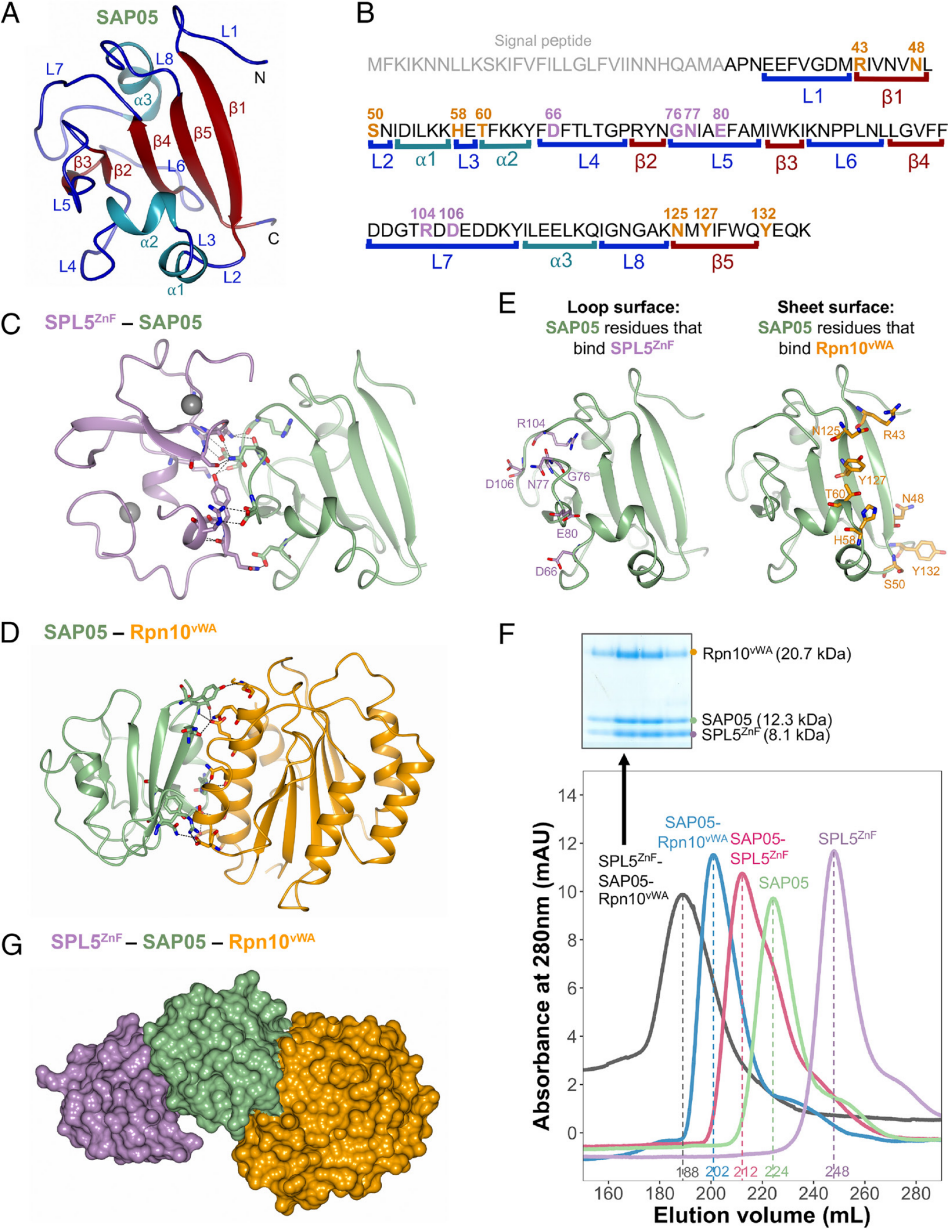
Crystal structures of SAP05–SPL5^ZnF^ and SAP05–Rpn10^vWA^ complexes revealing the bimodular architecture of SAP05. (*A*) Cartoon model illustrating the fold of the SAP05 effector. α-helices, β-sheets, and loops are indicated in cyan, red, and blue, respectively. (*B*) Amino acid sequence of SAP05 highlighting the locations of secondary structures (shown in *A*, color matched) and SPL5^ZnF^ (purple) and Rpn10^vWA^ interacting residues (orange) (shown in *C*–*F*, color-matched). SPL5^ZnF^, zinc-finger domain of SPL5 transcription factors. Rpn10^vWA^, von Willebrand factor type A domain of Rpn10 ubiquitin receptor. (*C*) Crystal structure of the SAP05–SPL5^ZnF^ complex (PDB 8PFC). The Zn^2+^ ions bound to the two ZnF domains are shown in gray. (*D*) Crystal structure of the SAP05–Rpn10^vWA^ complex (PDB 8PFD). In (*C*) and (*D*), the dashed lines indicate the interactions between the residues from both components. (*E*) Interfaces of SAP05 showing the loop surface (purple) and sheet surface (orange) residues that bind to SPL5^ZnF^ and Rpn10^vWA^, respectively. (*F*) Gel filtration chromatograms of SAP05, SPL5^ZnF^, and Rpn10^vWA^ and binary and ternary complexes of these proteins. The Coomassie-stained protein SDS-PAGE gel shows the presence of the three proteins in the SPL5^ZnF^–SAP05–Rpn10^vWA^ ternary complex—see *SI Appendix*, Fig. S1, for the other complexes. Elution volumes are indicated at the bottom of each peak with the same colors. (*G*) Hypothetical ternary structure of SPL5^ZnF^–SAP05–Rpn10^vWA^ obtained by superimposing the crystal structures of SAP05–SPL5^ZnF^ and SAP05–Rpn10^vWA^ complexes.

The SAP05 protein comprises a globular compact structure with five β-strands that form an internal triangular mixed β-sheet core (Fig. 1 *A* and *B*). β-strand 1 (β1) locates on the long end of this triangular core and connects via a loop–helix–loop–helix–loop structure to β2 at the opposite surface near the tip of this core. This β-strand is connected to β3 and β4 via loop structures that form the loop-dominated surface of the protein. β4 then connects to a loop-helix-loop structure that runs back to near the long end of the core to β5, which runs parallel to β1 (Fig. 1*A*). Both the SAP05–SPL5^ZnF^ and SAP05–Rpn10^vWA^ structures comprise a 1:1 complex (Fig. 1 *C* and *D*). The SAP05 residues binding ZnF and vWA are located at opposing surfaces of the effector with the ZnF binding surface comprising the loop-dominated surface (loop surface) and the vWA-binding surface the β-sheet-dominated long end of the triangular β-sheet core (sheet surface) (Fig. 1 *B* and *E*).

We were able to assemble the ternary complex containing ZnF, SAP05 and vWA by mixing equimolar amounts of the purified SAP05–Rpn10^vWA^ complex and purified SPL5^ZnF^ followed by gel filtration (Fig. 1*F* and *SI Appendix*, Fig. S1*B*), as shown previously (5). Therefore, SAP05 has the capacity to bind ZnF and vWA simultaneously to form a ternary complex (Fig. 1*G*). The SAP05 structures are essentially the same between the SAP05–SPL5^ZnF^ and SAP05–Rpn10^vWA^ complexes (rmsd = 0.257 Å) (Fig. 1 *C* and *D*). Moreover, there is no steric hindrance between the ZnF and vWA domains when bound to SAP05. These data provide evidence that SAP05 has a bimodular architecture with opposite loop and sheet surfaces that enable interactions with ZnF of TFs and vWA of Rpn10, thereby acting as a scaffold to link SPL and GATA TFs to Rpn10.

### The SAP05 “Loop Surface” Forms Electrostatic Interactions with the ZnF Domain.

**Fig. 2.**
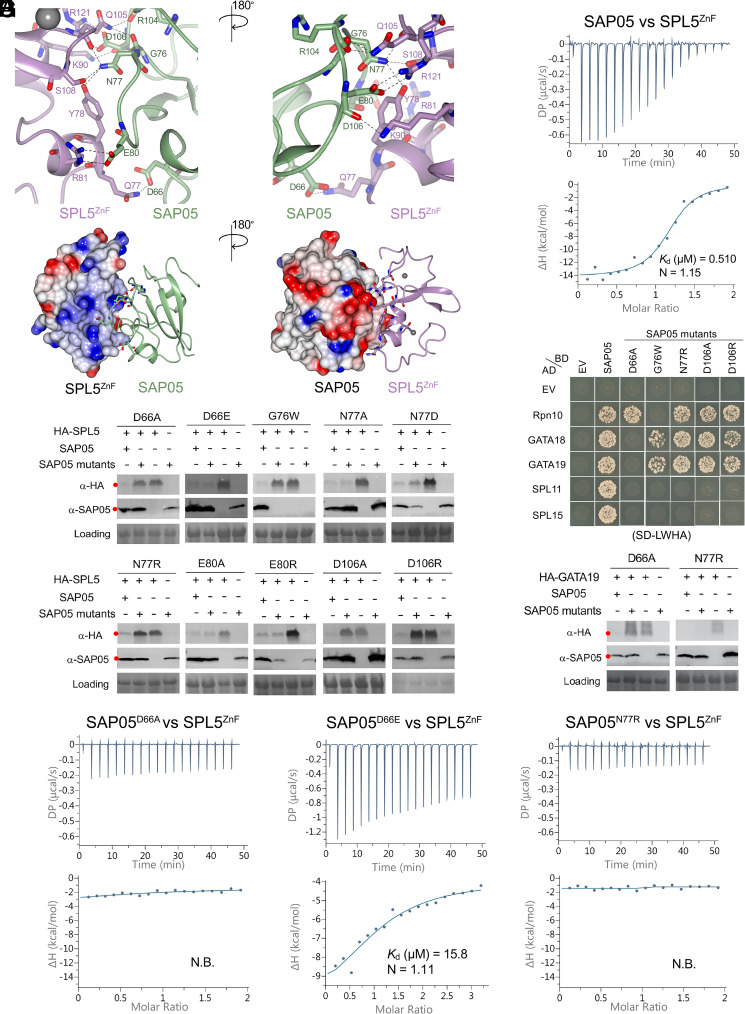
SAP05 loop surface interacts with SPL5^ZnF^. (*A*) Close-up views of SAP05–SPL5^ZnF^ interaction interface from front (*Left*) and back (*Right*). The amino acid residues mediating electrostatic interactions are labeled. The Zn^2+^ ions are displayed as gray spheres. (*B*) Electrostatic potential surface view of SAP05 and SPL5^ZnF^ during complex formation showing that the interacting interface is predominantly electronegative (red) in SAP05 and electropositive (blue) in SPL5^ZnF^. (*C*) ITC experiment showing direct physical binding of SAP05 and SPL5^ZnF^. (*D*) Western blot analysis of proteasomal degradation of SPL5 in the presence of wild-type or mutant SAP05 in *N. benthamiana* leaves. (*E*) Yeast two-hybrid (Y2H) assay to test interactions of SAP05 and its mutant versions with *A. thaliana* Rpn10 and GATA and SPL TFs. EV, empty vector control. AD, GAL4-activation domain. BD, GAL4-DNA binding domain. SD-LWHA, quadruple dropout medium lacking leucine, tryptophan, histidine, and adenine. Yeast growth on SD-LW medium is shown in *SI Appendix*, Fig. S6*A*. (*F*) Western blot analysis for GATA19 degradation in the presence of SAP05 D66A or N77R mutants in *N. benthamiana* leaves. In (*D*) and (*F*), red dots indicate the expected sizes of the transiently expressed proteins. HA, hemagglutinin. Protein loading was visualized using Ponceau S staining. (*G*) ITC titrations of three SAP05 mutants (D66A, D66E, and N77R) with SPL5^ZnF^. N.B., not binding. In (*C*) and (*G*), the top panels show heat differences upon interaction, and lower panels show integrated heats of injection (•) and the best fit to a single site binding model using MicroCal PEAQ-ITC analysis software. See *SI* *Appendix*, Fig. S5, for more ITC repeats and thermodynamic parameters.

We further investigated the SAP05 interaction with SPL5^ZnF^. The crystal structure contains eight copies of the 1:1 complex in the asymmetric unit (ASU), which are closely similar (*SI Appendix*, Fig. S2). In addition to the two structural Zn^2+^ ions within each SPL5^ZnF^ domain, a further four Zn^2+^ ions are found in the ASU forming crystal contacts (*SI Appendix*, Fig. S2). These involve E80 from four of the eight SAP05 molecules and H82 and E118 from separate neighbouring copies of SPL5^ZnF^. The SAP05 loop surface that interacts with ZnF comprises three distinct protruding loops that are separated by β-strands and involves six amino acids of which D66 locates in loop 4, G76, N77, and E80 in loop 5, and R104 and D106 in loop 7 of the SAP05 structure (Figs. 1 *A*, *B*, and *E* and 2*A*). SAP05 binds the two SPL5 ZnF sites, which are held together by Zn^2+^ ions (Figs. 1*C* and 2*A*) and the complex involve nine connections, with SAP05 amino acids N77 interacting with three amino acids, D106 with two and SAP05 D66, G76, E80, and R104 each with one amino acid of SPL5^ZnF^, and SAP05 E80 and D106 each forming two interactions with R81 and R121, respectively, of SPL5^ZnF^ (Fig. 2*A* and *SI Appendix*, Fig. S3*A* and S4 *A* and *B*). The majority of the interactions are mediated by hydrogen bonds with some salt bridges and are reinforced by nonbonded contacts (*SI Appendix*, Fig. S4*A* and Table S1). Distances between the interacting residues range from 2.63 to 3.53 Å (*SI Appendix*, Fig. S4 *B* and *C*). The SAP05–ZnF interaction is dominated by electrostatic interactions of charged and polar residues at the SAP05 loop surface and oppositely charged or polar residues located within α-helices of SPL5^ZnF^ (Fig. 2*B*). To explore the affinity of interaction between SAP05 and SPL5^ZnF^, we used isothermal titration calorimetry (ITC). Titration of SAP05 into a solution of SPL5^ZnF^ showed an exothermic binding isotherm with a fitted dissociation equilibrium constant (*K*_d_) of 0.45 ± 0.06 µM and stoichiometry of 1:1 (Fig. 2*C* and *SI Appendix*, Fig. S5), and titrating SPL5^ZnF^ into a solution of SAP05 showed a *K*_d_ of 0.52 ± 0.05 µM (*SI Appendix*, Fig. S5).

We generated 10 structure-guided SAP05 mutants by replacing each residue that forms contacts across the interface with a neutral, similarly charged, or oppositely charged residue (*SI Appendix*, Fig. S3*A*). The mutants were investigated for their ability to degrade TFs in *Nicotiana benthamiana* leaves using *Agrobacterium*-mediated transient expression assays. SAP05 D66A, G76W, N77R, D106A, and D106R failed to degrade SPL5, though G76W was not detected in leaves (Fig. 2*D*). Yeast two-hybrid (Y2H) assays confirmed that these mutants lost the ability to bind SPL TFs, and except for G76W, retained their affinity for Rpn10 (Fig. 2*E* and *SI Appendix*, Fig. S6). G76W, N77R, D106A, and D106R also retained the ability to bind GATA TFs unlike D66A that lost the ability to bind both SPLs and GATAs (Fig. 2*E* and *SI Appendix*, Fig. S6). Consistent with these binding activities in Y2H, D66A did not mediate degradation of GATA19 in leaves whereas N77R did (Fig. 2*F*). In ITC experiments, SAP05 D66A and N77R mutants did not interact with the ZnF domain of SPL5, whereas SAP05 D66E did, though with lower affinity of a *K*_d_ of 17.7 ± 2.7 µM than wild-type SAP05 (Fig. 2 *C* and *G* and *SI Appendix*, Fig. S5). Despite this lower affinity, SAP05 D66E mediates the degradation of SPL5 (Fig. 2*D*). Therefore, D66, N77, and D106 are required for the SAP05 interactions with the ZnF domain of SPLs. Moreover, D66 is also required for SAP05 interactions with GATA TFs. The finding that SAP05 G76W lost binding to both SPLs and Rpn10 suggests that this mutant has more profound structural changes, in agreement with its instability in *N. benthamiana* leaves (Fig. 2*D*). D66 is part of loop 4, N77 of loop 5, and D106 of loop 7 (Figs. 1 *A* and *B* and 2*A*), indicating that all three loop structures of the SAP05 loop surface are involved in binding ZnF of SPLs (Fig. 1 *C* and *E*), thereby validating the crystal structure.

Our finding that D66 on one of the loops (loop 4) is involved in binding GATA prompted us to use AlphaFold-Multimer modeling (35) to assess the SAP05–GATA19^ZnF^ complex structure. The AlphaFold model (AFM) of the SAP05 and SPL5^ZnF^complex and their interactions were consistent with the crystal structure (*SI Appendix*, Fig. S7*A*), suggesting a modeling approach could be insightful. We therefore proceeded to predict the SAP05–GATA19^ZnF^ structure using AFM and obtained a high prediction confidence score (*SI Appendix*, Fig. S7*B*). In the model, GATA19^ZnF^ interacts with the SAP05 loop surface (*SI Appendix*, Fig. S7*B*). Moreover, SAP05 D66, but not N77, is one of the residues involved in the interaction with GATA19 (*SI Appendix*, Fig. S7*C*), in agreement with the finding that SAP05 N77R degraded GATA19 in *N. benthamiana* leaves, unlike SAP05 D66A (Fig. 2*F*). SAP05 F65 of loop 4, E80 of loop 5, and D108 of loop 7 were also predicted to mediate interactions with GATA19 (*SI Appendix*, Fig. S7*C*).

Taken together, these data demonstrate that SAP05 interactions with SPL and GATA TFs involve structures of the SAP05 loop surface. Most SAP05 residues involved in binding SPLs and GATAs do not play a role in SAP05 binding of Rpn10 consistent with the bimodular architecture of SAP05.

### The SAP05 “Sheet Surface” Forms Polar Interactions with Rpn10^vWA^.

**Fig. 3.**
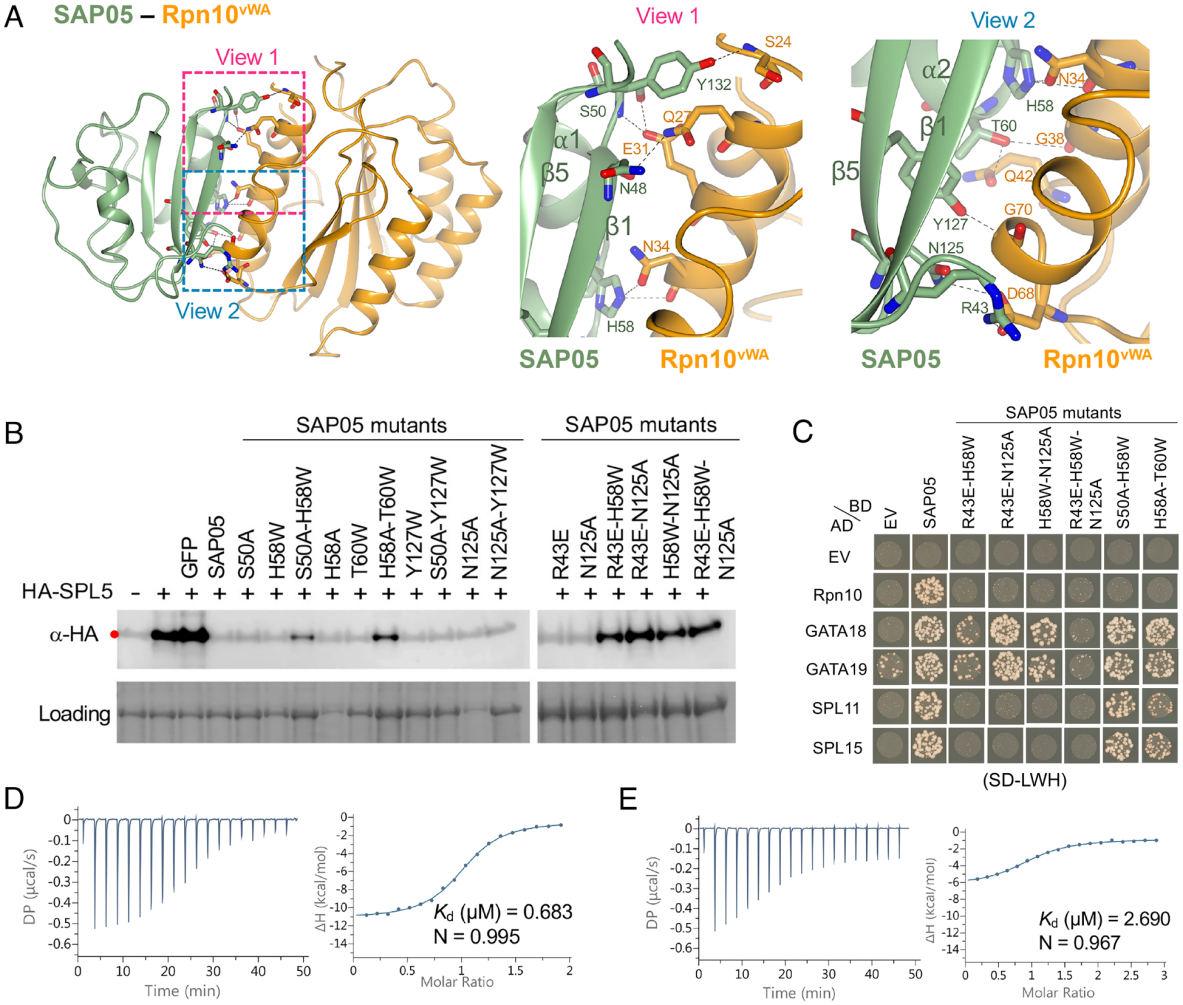
SAP05 β-sheet surface binds to Rpn10^vWA^. (*A*) Close-up views of SAP05–Rpn10^vWA^ interaction interface showing the residues involved in complex formation. *Left*, overview of the interacting interface with two dashed squares displaying the areas for enlarged view. *Middle*, the enlarged view 1 of the top part in the interface. *Right*, the enlarged view 2 of the lower part in the interface. (*B*) Western blot analysis for SPL5 degradation with SAP05 wild-type and mutants in *A. thaliana* protoplasts. GFP, green fluorescent protein control. HA, hemagglutinin. Protein loading was visualized using Amido Black staining. (*C*) Y2H assay to test interactions of SAP05 and its mutants with *A. thaliana* Rpn10 and GATA and SPL TFs. EV, empty vector control. AD, GAL4-activation domain. BD, GAL4-DNA binding domain. SD-LWH, triple dropout medium lacking leucine, tryptophan, and histidine. See *SI Appendix*, Fig. S6*B*, for yeast growth on SD-LW medium. (*D* and *E*) ITC titrations of the SAP05–Rpn10^vWA^ complex with SPL5^ZnF^ (*D*) and SAP05^H58A-T60W^ with SPL5^ZnF^ (*E*). Left panels show heat differences upon interaction and right panels show integrated heats of injection (•) and the best fit to a single site binding model using MicroCal PEAQ-ITC analysis software. See *SI* *Appendix*, Fig. S5, for more ITC repeats and thermodynamic parameters.

We then further investigated the role of specific residues involved in the SAP05–Rpn10^vWA^ interface. The SAP05 sheet surface comprises two parallel β-strands, β1 and β5, as well as two loops separated by an α-helix (Fig. 1 *A*, *B*, *D*, and *E*). The interaction is mediated by eight SAP05 amino acids, including R43 and N48 located on β1, S50 on loop 2, H58 on loop 3, T60 on α2, N125 and Y127 on β5, and Y132 on the loop following β5 of SAP05 (Fig. 3*A* and *SI Appendix*, Figs. S3*B* and S8). Each of these residues interacts with one residue of Rpn10^vWA^, except for T60 that interacts with two residues, and S50 and H58 form two interactions with E31 and N34 of Rpn10^vWA^, respectively (Fig. 3*A* and *SI Appendix*, Fig. S9 *A* and *B*). Four of the interactions involve double or single hydrogen bonds and one salt bridge, and other interactions comprise nonbonded contacts (*SI Appendix*, Fig. S9 *A*–*C* and Table S2). Distances between the interacting residues range from 2.48 to 3.76 Å (*SI Appendix*, Fig. S9 *B* and *C*). All Rpn10^vWA^ residues that interact with SAP05 locate on α-helices (Fig. 3*A*). The Rpn10^vWA^ interaction is largely mediated by polar forces. Thus, the SAP05 sheet surface contains rigid secondary β-sheets and α-helix structures that are held in place by hydrogen bonds within SAP05, as opposed to the ZnF-binding loop surface that involves protruding loop structures that may be more flexible.

We introduced single amino acid mutations in the SAP05 sheet surface by replacing each with neutral, nonpolar or oppositely charged residues generating nine single amino acid SAP05 mutants (*SI Appendix*, Fig. S3*B*). All retained the ability to degrade SPL5 in *N. benthamiana* leaves, though some of the SAP05 mutants were not detected in leaves and hence may have high turnover rates (*SI Appendix*, Fig. S10). However, several SAP05 double mutants and one triple mutant had reduced or no ability to mediate degradation of SPL5 (Fig. 3*B*), consistent with their loss of binding to Rpn10 in Y2H (Fig. 3*C*). SAP05 H58A T60W and S50A H58W retained affinity to SPL and GATA TFs (Fig. 3*C*), indicating that these double mutations have less impacts on the overall structure of SAP05, compared to the other double mutants that lost affinity to SPLs and the triple mutant that did not bind any of the targets. These data indicate that multiple SAP05 residues mediate interactions with vWA, thereby validating the crystal structure.

In ITC experiments, the interaction of the SAP05–Rpn10^vWA^ complex with SPL5^ZnF^ has a *K*_d_ of 0.65 ± 0.04 µM and stoichiometry of 1:1 (Fig. 3*D* and *SI Appendix*, Fig. S5), which is similar to that of SAP05 alone with SPL5^ZnF^ (Fig. 2*C* and *SI Appendix*, Fig. S5). This indicates that vWA-bound SAP05 does not undergo a major change in configuration compared to SAP05 alone, in agreement with crystal structure data (Fig. 1 *C* and *D*). However, the *K*_d_ of the SAP05 H58A T60W interaction with SPL5^ZnF^ is 3.60 ± 0.81 µM (Fig. 3*E* and *SI Appendix*, Fig. S5), indicating that the H58A T60W mutations impact the efficiency of SAP05 to bind SPL5^ZnF^. Nonetheless, SAP05 H58A T60W retains affinity to SPL and GATA TFs and mediates the degradation of SPL5 (Fig. 3 *B* and *C*), in line with the bimodular architecture of SAP05.

### Conservation Analyses of SAP05 Residues Involved in SAP05–ZnF and SAP05–vWA Interaction Reveal Dynamic Evolutionary Patterns.

We previously identified SAP05 homologs in phytoplasmas, including examples that bind both SPLs and GATAs and ones that bind only SPLs or only GATAs (5). SAP05 amino acids that interact with SPL5 and vWA are conserved among all or most SAP05 homologs, including D66 that mediates binding with SPL and GATA TFs (*SI Appendix*, Fig. S11*A*). The SAP05 homologs that exclusively bind SPLs (PnWBa, WBDLa, and P. mali) and that bind only GATAs (PnWBb and WBDLb) exhibited the greatest sequence variation in their interacting residues compared to other SAP05 homologs (*SI Appendix*, Fig. S11*A*). We noticed that regions corresponding to SAP05 residues 67 to 73 (FTLTGPR) that form loop 4 and connect to β2 in SAP05_AYWB (Fig. 1 *A* and *B*) were different in sequence among the SPL versus GATA-binding SAP05 homologs (*SI Appendix*, Fig. S11*A*). Loop 4 starts with the conserved F65 and D66 amino acids (Fig. 1 *A* and *B* and *SI Appendix*, Fig. S11*A*). Given our finding herein that D66 is essential for both SPL and GATA binding and degradation (Fig. 2), we investigated whether loop 4 is involved in determining SAP05-binding specificity for SPLs and GATAs. Swapping corresponding loop 4 sequences from SAP05 homologs PnWBa and WBDLa to PnWBb and WBDLb resulted in gain of SPL binding, and conversely, swapping these from PnWBb and WBDLb to PnWBa and WBDLa resulted in gain of GATA binding (*SI Appendix*, Fig. S11 *B* and *C*). Therefore, loop 4 contributes to SAP05 binding specificity to SPLs and GATAs.

Next, we determined in how far interacting residues are conserved among the ZnF domains of SPL TFs. The zinc-binding domain of SPL proteins contains two zinc-binding sites formed by eight conserved Cys or His residues (31). SAP05-binding residues locate in both ZnF domains and the majority of these are conserved among the SPL TFs (*SI Appendix*, Fig. S12). However, SAP05 D66 interacts with Q77 in SPL5, and this amino acid is not conserved (*SI Appendix*, Fig. S12). Given the importance of SAP05 D66 in mediating interactions with both SPL and GATAs, we examined the electrostatic surface in the position of SAP05 D66. In wild-type SAP05, the surface area around D66 is electronegative (*SI Appendix*, Fig. S13 *A* and *C*), while the electronegativity of this surface area is reduced in the SAP05 D66A mutant that lost interaction with SPL5 and GATA19 (*SI Appendix*, Fig. S13 *B* and *D*). Furthermore, the SAP05 D66E mutant, which is equally charged with negative residue glutamic acid, still binds and degrades SPL5 in planta (Fig. 2 *D* and *G*). Taken together, these results indicate that the electronegative surface potential contributed by D66 is an important factor for binding SPL and GATA TFs.

**Fig. 4.**
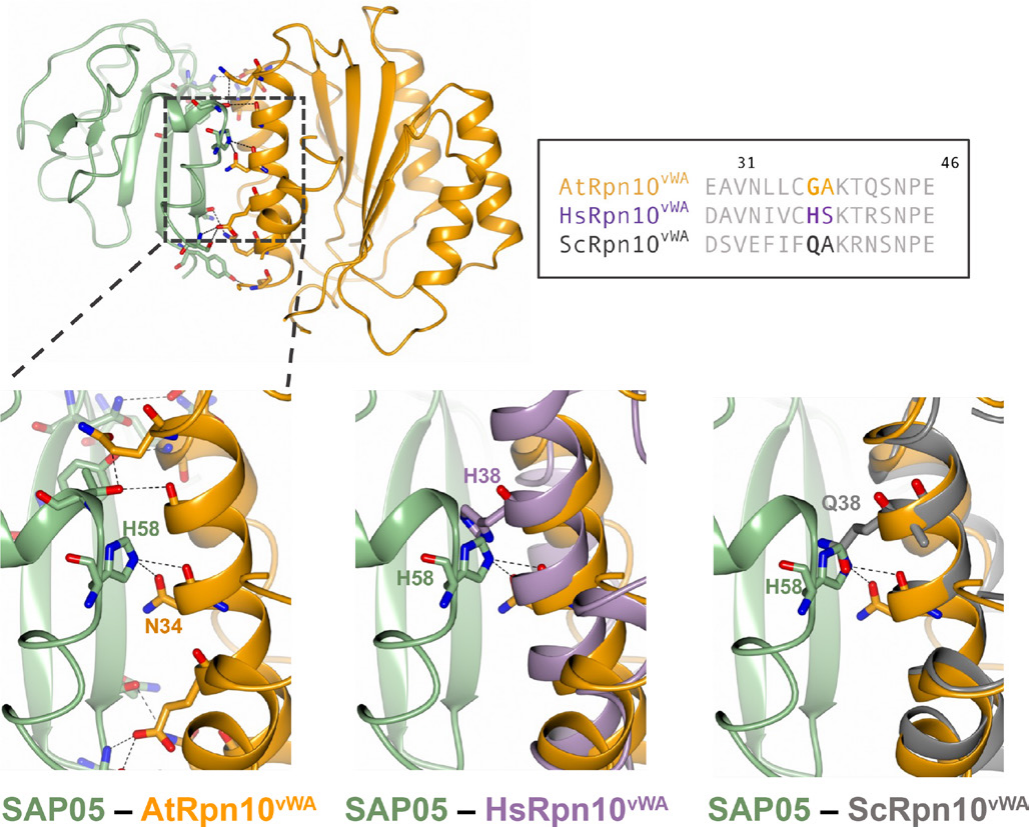
Steric clash at the SAP05 loop 3 region prevents SAP05 binding to human and yeast vWA domains. The SAP05–AtRpn10^vWA^ complex structure was aligned with the vWA domain structures of human Rpn10 (PSMD4, PDB 6MSD) and yeast (*S. cerevisiae*) Rpn10 (PDB5LN1) to compare their configurations. The dashed squared box on the top left shows the position of loop 3. Box on the top right, sequence alignment of vWA sequences from *A. thaliana* Rpn10 (Uniprot ID: P55034); HsRpn10, *H. sapiens* Rpn10 (Uniprot ID: Q5VWC4); ScRpn10, *S. cerevisiae* Rpn10 (Uniprot ID: P38886).

Among the SAP05 residues that bind vWA, particularly H58, in combination with S50 or T60, have essential roles (Fig. 3). These locate in loop 2 and loop 3 that connect α1 and α2 in the SAP05 structure (Fig. 1 *A* and *B*). SAP05 H58 is conserved among the majority of SAP05 homologs (*SI Appendix*, Fig. S7*A*) and interacts with N34 that is conserved in vWA domains of most Rpn10 homologs (*SI Appendix*, Fig. S14). SAP05 S50 interacts with *A. thaliana* vWA E31 that is also an acidic amino (D) in other vWA sequences, and SAP05 T60 with G38 and Q42 that are conserved among plant vWA (*SI Appendix*, Fig. S14). The four amino acids locate in the first α-helix (positions 25 to 43) at the N terminus of *A. thaliana* vWA (Fig. 4 and *SI Appendix*, Fig. S14).

We previously reported that SAP05 does not interact with insect and human Rpn10 (the latter is also known as PSMD4) and that replacing AtvWA G38 and A39 with human H38 and S39, respectively, prevented SAP05 binding (5). Multiple sequence alignment of plant and animal vWA domains showed high conservation with a few amino acid differences (*SI Appendix*, Fig. S14). Structural superimposition of SAP05 interactions with vWA domains of plant Rpn10 and PSMD4 revealed that SAP05 loop 3 clashes with H38 of PSMD4 precluding a SAP05-PSMD4 interaction (Fig. 4). Similarly, Q38 of vWA of yeast Rpn10 clashes with loop 3 of SAP05 precluding SAP05 from binding yeast Rpn10 (Fig. 4). This corroborates data presented herein that H58 of the SAP05 loop 3 region plays an essential role in the SAP05 interaction with *A. thaliana* vWA.

### Positioning of the SAP05 Complex on the 26S Proteasome Points to a TPD Mechanism.

**Fig. 5.**
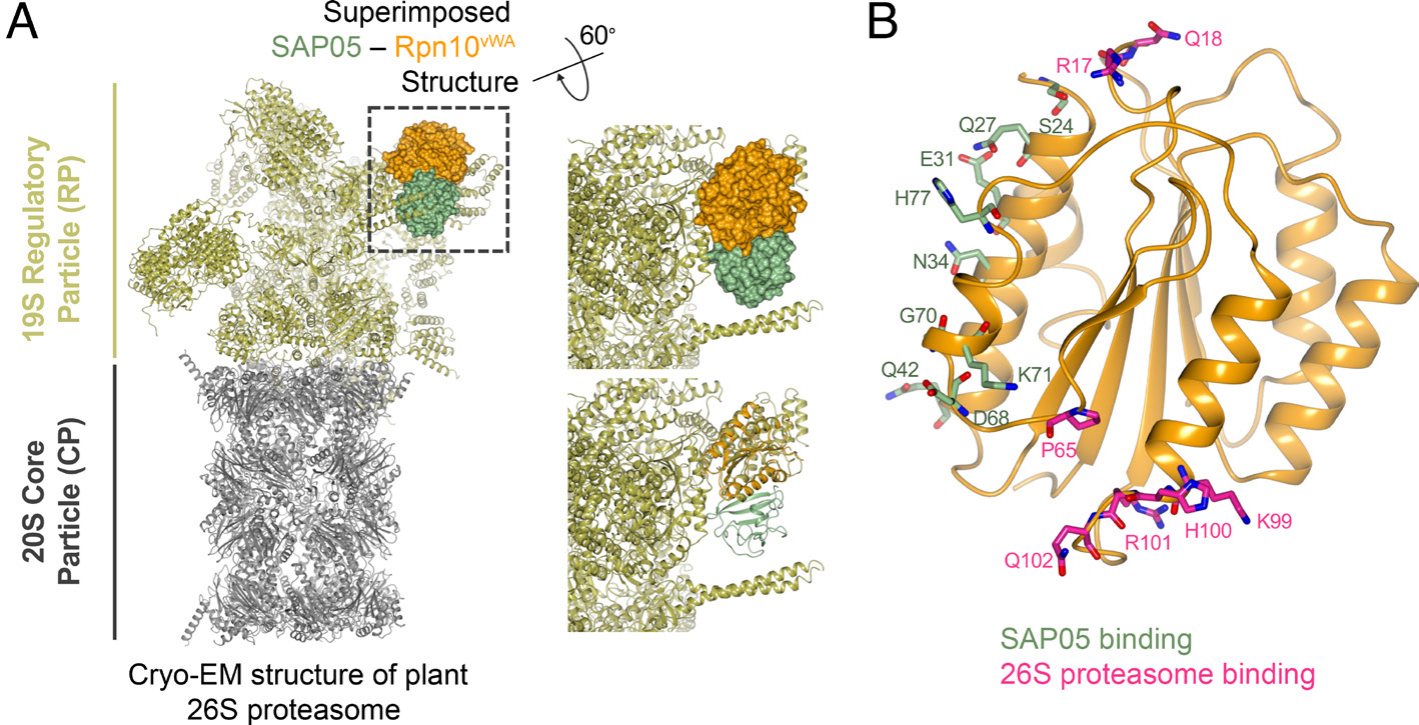
SAP05 interaction with the Rpn10^vWA^ domain has no hindrance on the plant 26S proteasome. (*A*) Structural superimposition of SAP05–Rpn10^vWA^ on the spinach 26S proteasome (PDB 7QVE and PDB 8AMZ). The dashed square box shows the parts with superimposition and is enlarged to show the surface (*Right Top*) and cartoon (*Right Bottom*) view of the SAP05–Rpn10^vWA^ complex. (*B*) Position of Rpn10^vWA^ residues interacting with SAP05 (green) and subunits of the 26S proteasome (pink).

Rpn10 is positioned in the 19S RP where it forms an important component in the interface of the lid and base structure (36). The cryo-EM structure of the spinach (*Spinacia oleracea*) 26S proteasome was recently resolved (32). The vWA residues that interact with 26S proteasome are conserved in *S. oleracea* and *A. thaliana* Rpn10 homologs (*SI Appendix*, Fig. S15). The structural superposition of SAP05–Rpn10^vWA^ complex onto the spinach 26S proteasome did not reveal obvious steric clashes with proteasome components (Fig. 5*A*). Notably, SAP05 interacts with two parallel α-helices that locate on a region of vWA that does not interact with the 19S subunit (Fig. 5*B*). This suggests that SAP05 has minimal disadvantageous effects on Rpn10 interactions with the 19S RP. However, the SPL5^ZnF^ of the ternary structure sterically clashes with two α-helices that protrude from the 26S proteasome (*SI Appendix*, Fig. S16*A*). We found that these helices are derived from the flexible N-terminal coiled-coil (CC) domains of the spinach homologs of *A. thaliana* Rpt4 and Rpt5 (*SI Appendix*, Fig. S16*A*), which are part of the 6 AAA+ ATPase (Rpt1-6) subunit ring that sits on top of the 20S CP. The CC domains of these Rpt proteins play an important role in physically connecting substrate recruitment and processing (37) and may therefore be involved in SPL5^ZnF^ binding and degradation. We propose that interaction of SPL5 with the CC dimer may induce the degradation of the transcription factor.

## Discussion

The crystal structure data reported herein demonstrate that the 12.3-kDa bacterial effector SAP05 has a globular structure with an internal β-sheet core and interconnecting loops and α-helices. One interaction surface is dominated by rigid β-strands (sheet surface) and the opposite surface by loop structures (loop surface) that may be more flexible in their configurations. The rigid sheet surface binds the vWA domain of plant Rpn10 and cannot bind vWA domains of Rpn10 homologs of several organisms other than plants, even though the vWA domains are highly conserved among Rpn10 homologs. The flexible loop surface is however capable of binding multiple transcription factors of the distinct SPL and GATA TF families via their zinc-finger domains. Therefore, SAP05 appears to have an optimal configuration to act as a molecular bridge capable of connecting a conserved proteasome component to multiple members of two TF families.

Rpn10^vWA^ has an important function as its deletion causes lethality or severe growth deficiencies in plants, vertebrates, and human cell lines (38–40). In *rpn10* null mutants, deficiencies may be restored upon addition of Rpn10^vWA^ and RAD23, and Rpn10 and RAD23 act redundantly (41, 42). Like Rpn10, RAD23 is a reversible component of the 26S proteasome and binds ubiquitinated substrates (41). Intriguingly, the phytoplasma SAP54/phyllogen family of effectors hijack RAD23 and mediates plant proteins, the MADS-box TFs for degradation, leading to plant developmental changes (29, 30). SAP54 bind the RAD23 ubiquitin-associated (UBA) that are shown to noncovalently bind ubiquitin moieties of ubiquitinated substrates (26, 43). Therefore, at least two distinct phytoplasma effector families mediate degradation of plant TFs in ubiquitin-independent manner using reversible components of the 19S RP.

The vWA domain locates at the N terminus of Rpn10, followed by a flexible region connecting single-helix ubiquitin interaction motifs (UIMs) that bind ubiquitin chains in the C-terminal half of Rpn10 (44, 45). Due to the flexibility of the C-terminal half of Rpn10, only the vWA domain is visible in cryo-EM structures of 26S proteasomes and locates in a central location of the 19S RP, at the interface of its base and lid (46). There is no evidence that SAP05 interferes with 26S proteasome activity (5) and structural information generated herein shows that SAP05 binds to a region of vWA that does not interact with components of the 19S RP. Therefore, SAP05 appears to have evolved to optimally bind plant Rpn10, which is positioned in a central location of the 19S RP.

Our findings indicate that the placement of the SAP05 complex on the 26S proteasome suggests a TPD mechanism. While the SAP05–Rpn10^vWA^ complex does not appear to cause significant steric hindrance, the SPL5^ZnF^ within the ternary structure appears to clash sterically with the α-helices of Rpt4 and Rpt5, as evidenced in the structural model of the spinach 26S proteasome (32). The coiled-coil (CC) domains of these two Rpt proteins, along with the domains of four others, dimerize to create three CCs (Rpt1/2, Rpt6/3, and Rpt4/5 CCs). These CCs play a vital role in 26S proteasome activity by physically linking substrate recruitment and processing to the unfolding machinery, ultimately leading to substrate degradation (47). Furthermore, conformational changes within the CCs are essential for transitioning the Rpt1-6 ATPase ring between resting and active states (37), the latter of which involves a widening of the central pore to allow substrate entry into the core of the 20S CP (47). The Rpt4/5 CC also binds to Rpn10 upon substrate binding (47). As such, when the ternary complex is accommodated on the 26S proteasome, the interaction of SPL5 with the CC dimer could potentially instigate the switch of the proteasome to the active state, leading to the degradation of the transcription factor.

The ternary structure was modeled on the spinach 26S proteasome recognition state structure. However, the 26S proteosome changes configuration during the processing of substrates. To investigate how the SAP05 ternary complex may be positioned on the different configurations of the 26S proteasome, we used the structures of the human 26S proteasome substrate recognition (PDB 6MSD), deubiquitination (PDB 6MSE) and translocation initiation (PDB 6MSH) states that have been resolved (48) for modeling. The CC domains of Rpt4 and Rpt5 also clash with SPL5^ZnF^ of the superimposed SAP05 ternary complex on the substrate recognition and deubiquitination states, whereas they clash with SAP05 in the translocation initiation state of the human 26S proteasome (*SI Appendix*, Fig. S16*B*). However, the Rpt subunits are known to undergo conformational changes to hold the substrate in place (37, 49). Therefore, it remains to be determined where the CC domains locate in the presence of the SAP05 ternary complex. SAP05 may also disengage once SPL5 is starting its translocation into the central pore for degradation. Another consideration is that full-length SPL5 is likely to cause more clashes with proteasome components than SPL5^ZnF^. However, AlphaFold2 predictions show that the regions that directly flank the ZnF domain at both ends of the SPL5 protein are highly unstructured (*SI Appendix*, Fig. S17), indicating that these regions might be inherently flexible thereby enabling SPL5 accommodation on the proteasome.

We investigated the importance of residues mediating the SAP05–SPL5^ZnF^ and SAP05–Rpn10^vWA^ interactions. We found that mutations of single amino acids in SAP05 disrupted the SAP05–SPL5^ZnF^ interaction. Some mutations in SAP05 had little impact on SPL5 stability. These include N77A and N77D. However, another mutation involving the same amino acid, such as N77R, abolished the ability of SAP05 to bind SPL5^ZnF^ and mediate the degradation of SPL5. An explanation is that neutral (A) and negatively charged (D) amino acids at the N77 location do not affect SPL5 binding, whereas a positively charged amino acid (R) does. This agrees with our observation that an electronegative surface of SAP05 is primarily responsible for binding the TFs. SAP05 mutant D66A is impaired in the degradation of both SPL5 and GATA19, showing a potential to be further engineered for a useful tool to degrade any protein without inducing plant developmental changes.

In contrast, multiple amino acid mutations are needed to impair SAP05 interaction with Rpn10^vWA^. Even in the SAP05 H58A T60W and S50A H58W double mutants, the ability to mediate degradation has not fully disappeared. This suggests that the SAP05–Rpn10^vWA^ interaction is robust. It is striking that SAP05 itself is not degraded, and is highly stable in plant cells, despite its association with the 26S proteasome and its function in degrading substrates that it directly interacts with. However, the study herein revealed that SAP05 derivatives with mutations in the SAP05–Rpn10^vWA^ interaction surface are often unstable in plant cells. A possible explanation of these results is that, compared to wild-type SAP05, the SAP05 mutants have weaker interactions with vWA and this leads to the SAP05 mutant being dragged down along with ZnF when the latter is pulled into the core of the 20S CP. We were unable to test this with ITC experiments because purified vWA on its own (without SAP05) forms aggregates. Nonetheless, results so far suggest that SAP05 stability is linked to its association with Rpn10. During the revision of this manuscript, other SAP05 crystal structures were resolved, including that of the SAP05 homologue of Onion Yellow phytoplasma in complex with Rpn10^vWA^ (50), revealing similar structures as reported herein.

In summary, SAP05 displayed no notable structural similarity to any experimentally confirmed structures in the Protein Data Bank (PDB). We suggest that this effector protein possesses a molecular architecture that enables it to connect host proteins to the Ubiquitin Proteasome System (UPS), thereby circumventing the canonical targeted protein degradation pathway. Impaired functioning of the UPS is linked with a multitude of diseases. However, given the pivotal role of the UPS in orchestrating an array of cellular processes, the SAP05 molecular system presents opportunities for the development of innovative therapeutics. Notably, Rpn10 has been recognized as a potential therapeutic target (51). Furthermore, the 26S proteasome has been leveraged to create therapeutics such as PROTACs (PROteolysis-TArgeting Chimeras), which are small molecules that recruit E3 ligases for ubiquitination of substrates marked for degradation (52). Several PROTACs and similar systems have shown promising results and are currently advancing through clinical trials. However, their reliance on recruiting E3 ligases has led to challenges associated with side effects and resistance. Since SAP05 does not hamper the ability of the 26S proteasome to degrade substrates, this effector emerges as a prime candidate for engineering a type of degraders that operate independently of E3 ligases. The structural work presented in this study provides a springboard for bioengineering a class of degraders that do not depend on E3 ligases.

## Materials and Methods

### Gene Cloning for Crystallization.

For the purpose of crystallization of the SAP05–SPL5^ZnF^ and SAP05–Rpn10^vWA^ complexes, gene fragments corresponding to SAP05 (Ala33-Lys135) and ZnF domain of SPL5 (Ser60-Leu127) were separately subcloned to various pOPINF vectors, and SAP05 (Ala33-Lys135) and Rpn10^vWA^ (Val2-GLy193) were co-subcloned to pOPINA vectors using In-fusion cloning strategy (53, 54) as described in *SI Appendix*, *Materials and Methods*.

### SAP05 Mutation Generation.

SAP05 mutants were generated by overlap PCR (55) or directly synthesized using gBlock from Integrated DNA Technologies company (IDT). Mutations used for different assays were codon optimized and cloned to different vectors.

### Protein Expression and Purification.

Plasmids expressing the SAP05, SPL5^ZnF^, SAP05, and Rpn10^vWA^ were expressed in *E. coli* BL21 (DE3) competent cells. Proteins were purified via IMAC followed by gel filtration. Purified proteins were pooled and concentrated to 15 mg/mL for crystallization studies. Detailed procedures are provided in *SI Appendix*, *Materials and Methods*.

### Protein Crystallization, Structure Determination, and Refinement.

Crystallization screens were performed in sitting-drop vapor diffusion format in MRC2 96-well crystallization plates. The SAP05–SPL5^Zn^ complex crystallized from 0.1 M MES pH 6.5, 25% (w/v) PEG 6000 in space group *P*2_1_. The SAP05–Rpn10^vWA^ complex crystallized from 0.1 M Sodium HEPES pH 7.5, 10.7 % (w/v) PEG 4000 in space group *P*2_1_. X-ray data were recorded on beamline I04 at the Diamond Light Source using an Eiger2 XE 16 M hybrid photon counting detector (Dectris), with crystals maintained at 100 K by a Cryojet cryocooler (Oxford Instruments). Data collection statistics are summarized in Table 1. Detailed procedures are described in *SI Appendix*, *Materials and Methods*.

### ITC.

ITC experiments were performed with MicroCal PEAQ-ITC instrument. The data were recorded at 25 °C using 20 mM HEPES, pH 7.5, and 150 mM NaCl buffer. To test the interaction of wild-type SAP05 and SPL5^ZnF^, the titration was done in both ways, with 20 μM SPL5^ZnF^ filled in calorimetric cell and 200 μM SAP05 from the syringe or 20 μM SAP05 filled in calorimetric cell and 250 μM SPL5^ZnF^ from the syringe. To test the interaction of SAP05–Rpn10^vWA^ complex or structure-guided SAP05 mutants and SPL5^ZnF^, the calorimetric cell was filled with 20 μM SAP05–Rpn10^vWA^ complex or SAP05 mutants and titrated with 200 μM SPL5^ZnF^ from the syringe. A single injection of 0.4 μL was followed by 19 injections of 2 μL each. Injections were made at 150-s interval with a stirring speed of 750 rpm. Each experiment was repeated two or three times. The raw data were integrated and fitted to a one-site binding model using the built-in software of MicroCal PEAQ ITC.

### Yeast Two-Hybrid Assay (Y2H).

The coding sequences of SAP05 or SAP05 mutants excluding signal peptides were amplified and ligated into Gateway vector pDEST-GBKT7 (BD). Constructs used to test protein–protein interactions were cotransformed into the yeast *Saccharomyces cerevisiae* strain AH109. Yeast growth was assessed on solid double dropout medium lacking leucine and tryptophan (SD-LW). Interactions were screened on selective triple or quadruple dropout medium. Details are provided in *SI Appendix*, *Materials and Methods*.

### Degradation Assay in *N. benthamiana* Leaves or in *A. thaliana* Protoplast.

Degradation assay was performed in *N. benthamiana* using *Agrobacterium*-mediated transient expression or in *A. thaliana* (Col-0) mesophyll protoplast and assessed by western blots. Details are described in *SI Appendix*, *Materials and Methods*.

### Homology Analysis.

Sequences of SAP05 homologs, *A. thaliana* SPLs, and Rpn10 from different organisms were aligned using MUSCLE algorithm on Phylogeny.fr web server (56) (https://www.phylogeny.fr/index.cgi). Graphical representation and editing were performed with MEGA11 software (57).

### Structural Analysis.

Structural predictions for protein complexes were conducted with AlphaFold2 (58) and AlphaFold-Multimer v3 (35). Analysis of predicted structure was performed with CCP4mg (59) and PDBsum tools (60).

## Supplementary Material

Appendix 01 (PDF)

## Data Availability

Two PDB X-ray structures data have been deposited in SAP05-ZnF and SAP05-vWA. Crystal structure data are available in the Protein Data Bank [PDB ID code 8PFC (61) and 8PFD (62)]. All other data are included in the manuscript and/or *SI Appendix*.

## References

[1] S. A. Hogenhout, R. A. Van der Hoorn, R. Terauchi, S. Kamoun, Emerging concepts in effector biology of plant-associated organisms. Mol. Plant Microbe Interact. **22**, 115–122 (2009). 19132864 10.1094/MPMI-22-2-0115

[2] A. J. Bogdanove, D. F. Voytas, TAL effectors: Customizable proteins for DNA targeting. Science **333**, 1843–1846 (2011). 21960622 10.1126/science.1204094

[3] P. Wei , Bacterial virulence proteins as tools to rewire kinase pathways in yeast and immune cells. Nature **488**, 384–388 (2012). 22820255 10.1038/nature11259 PMC3422413

[4] F. J. Mojica, F. Rodriguez-Valera, The discovery of CRISPR in archaea and bacteria. FEBS J. **283**, 3162–3169 (2016). 27234458 10.1111/febs.13766

[5] W. Huang , Parasitic modulation of host development by ubiquitin-independent protein degradation. Cell **184**, 5201–5214.e12 (2021). 34536345 10.1016/j.cell.2021.08.029 PMC8525514

[6] A. J. Barrett, Proteases. Curr. Protoc. Protein Sci. **21**, 21.1.1–21.1.12 (2000). 10.1002/0471140864.ps2101s21 18429162

[7] A. Rousseau, A. Bertolotti, Regulation of proteasome assembly and activity in health and disease. Nat. Rev. Mol. Cell Biol. **19**, 697–712 (2018). 30065390 10.1038/s41580-018-0040-z

[8] J. A. M. Bard , Structure and function of the 26S proteasome. Annu. Rev. Biochem. **87**, 697–724 (2018). 29652515 10.1146/annurev-biochem-062917-011931 PMC6422034

[9] S. Bhattacharyya, H. Yu, C. Mim, A. Matouschek, Regulated protein turnover: Snapshots of the proteasome in action. Nat. Rev. Mol. Cell Biol. **15**, 122–133 (2014). 24452470 10.1038/nrm3741 PMC4384331

[10] A. Ciechanover, Intracellular protein degradation: From a vague idea thru the lysosome and the ubiquitin-proteasome system and onto human diseases and drug targeting. Best Pract. Res. Clin. Haematol. **30**, 341–355 (2017). 29156207 10.1016/j.beha.2017.09.001

[11] G. A. Collins, A. L. Goldberg, The logic of the 26S proteasome. Cell **169**, 792–806 (2017). 28525752 10.1016/j.cell.2017.04.023 PMC5609836

[12] D. Finley, Recognition and processing of ubiquitin-protein conjugates by the proteasome. Annu. Rev. Biochem. **78**, 477–513 (2009). 19489727 10.1146/annurev.biochem.78.081507.101607 PMC3431160

[13] S. Murata, H. Yashiroda, K. Tanaka, Molecular mechanisms of proteasome assembly. Nat. Rev. Mol. Cell Biol. **10**, 104–115 (2009). 19165213 10.1038/nrm2630

[14] W. Baumeister, J. Walz, F. Zühl, E. Seemüller, The proteasome: Paradigm of a self-compartmentalizing protease. Cell **92**, 367–380 (1998). 9476896 10.1016/s0092-8674(00)80929-0

[15] C. Davis, B. L. Spaller, A. Matouschek, Mechanisms of substrate recognition by the 26S proteasome. Curr. Opin. Struct. Biol. **67**, 161–169 (2021). 33296738 10.1016/j.sbi.2020.10.010 PMC8096638

[16] T. Inobe, A. Matouschek, Paradigms of protein degradation by the proteasome. Curr. Opin. Struct. Biol. **24**, 156–164 (2014). 24632559 10.1016/j.sbi.2014.02.002 PMC4010099

[17] K. Tanaka, The proteasome: Overview of structure and functions. Proc. Jpn. Acad. Ser. B Phys. Biol. Sci. **85**, 12–36 (2009). 10.2183/pjab.85.12 PMC3524306 19145068

[18] K. H. Darwin, Prokaryotic ubiquitin-like protein (Pup), proteasomes and pathogenesis. Nat. Rev. Microbiol. **7**, 485–491 (2009). 19483713 10.1038/nrmicro2148 PMC3662484

[19] A. U. Müller, E. Weber-Ban, The bacterial proteasome at the core of diverse degradation pathways. Front. Mol. Biosci. **6**, 23 (2019). 31024929 10.3389/fmolb.2019.00023 PMC6466877

[20] O. A. Buneeva, A. E. Medvedev, Ubiquitin-independent protein degradation in proteasomes. Biomed. Khim. **64**, 134–148 (2018). 29723144 10.18097/PBMC20186402134

[21] Y. Mao, Structure, dynamics and function of the 26S proteasome. Subcell. Biochem. **96**, 1–151 (2021). 33252727 10.1007/978-3-030-58971-4_1

[22] G. Langin, S. Üstün, A pipeline to monitor proteasome homeostasis in plants. Methods Mol. Biol. **2581**, 351–363 (2023). 36413330 10.1007/978-1-0716-2784-6_25

[23] F. Q. Xu, H. W. Xue, The ubiquitin-proteasome system in plant responses to environments. Plant Cell Environ. **42**, 2931–2944 (2019). 31364170 10.1111/pce.13633

[24] D. Komander, M. Rape, The ubiquitin code. Annu. Rev. Biochem. **81**, 203–229 (2012). 22524316 10.1146/annurev-biochem-060310-170328

[25] Y. Leestemaker, H. Ovaa, Tools to investigate the ubiquitin proteasome system. Drug Discov. Today Technol. **26**, 25–31 (2017). 29249239 10.1016/j.ddtec.2017.11.006

[26] Y. Saeki, Ubiquitin recognition by the proteasome. J. Biochem. **161**, 113–124 (2017). 28069863 10.1093/jb/mvw091

[27] H. Yu, A. Matouschek, Recognition of client proteins by the proteasome. Annu. Rev. Biophys. **46**, 149–173 (2017). 28301771 10.1146/annurev-biophys-070816-033719

[28] W. Huang, S. A. Hogenhout, Interfering with plant developmental timing promotes susceptibility to insect vectors of a bacterial parasite. bioRxiv [Preprint] (2022). 10.1101/2022.03.30.486463 (Accessed 20 April 2023).

[29] A. M. MacLean , Phytoplasma effector SAP54 hijacks plant reproduction by degrading MADS-box proteins and promotes insect colonization in a RAD23-dependent manner. PLoS Biol. **12**, e1001835 (2014). 24714165 10.1371/journal.pbio.1001835 PMC3979655

[30] Y. Kitazawa , A phytoplasma effector acts as a ubiquitin-like mediator between floral MADS-box proteins and proteasome shuttle proteins. Plant Cell **34**, 1709–1723 (2022). 35234248 10.1093/plcell/koac062 PMC9048881

[31] K. Yamasaki , A novel zinc-binding motif revealed by solution structures of DNA-binding domains of Arabidopsis SBP-family transcription factors. J. Mol. Biol. **337**, 49–63 (2004). 15001351 10.1016/j.jmb.2004.01.015

[32] S. Kandolf , Cryo-EM structure of the plant 26S proteasome. Plant Commun. **3**, 100310 (2022). 35576154 10.1016/j.xplc.2022.100310 PMC9251434

[33] L. Holm, A. Laiho, P. Törönen, M. Salgado, DALI shines a light on remote homologs: One hundred discoveries. Protein Sci. **23**, e4519 (2023). 10.1002/pro.4519 PMC9793968 36419248

[34] E. Krissinel, K. Henrick, Secondary-structure matching (SSM), a new tool for fast protein structure alignment in three dimensions. Acta Crystallogr. D Biol. Crystallogr. **60**, 2256–2268 (2004). 15572779 10.1107/S0907444904026460

[35] E. Richard , Protein complex prediction with AlphaFold-Multimer. bioRxiv [Preprint] (2022). 10.1101/2021.10.04.463034 (Accessed 20 April 2023).

[36] M. H. Glickman , A subcomplex of the proteasome regulatory particle required for ubiquitin-conjugate degradation and related to the COP9-signalosome and eIF3. Cell **94**, 615–623 (1998). 9741626 10.1016/s0092-8674(00)81603-7

[37] A. Snoberger, E. J. Brettrager, D. M. Smith, Conformational switching in the coiled-coil domains of a proteasomal ATPase regulates substrate processing. Nat. Commun. **9**, 2374 (2018). 29915197 10.1038/s41467-018-04731-6 PMC6006169

[38] A. S. Fatimababy , Cross-species divergence of the major recognition pathways of ubiquitylated substrates for ubiquitin/26S proteasome-mediated proteolysis. FEBS J. **277**, 796–816 (2010). 20059542 10.1111/j.1742-4658.2009.07531.x

[39] J. Hamazaki , Rpn10-mediated degradation of ubiquitinated proteins is essential for mouse development. Mol. Cell Biol. **27**, 6629–6638 (2007). 17646385 10.1128/MCB.00509-07 PMC2099239

[40] N. Rani, A. Aichem, G. Schmidtke, S. G. Kreft, M. Groettrup, FAT10 and NUB1L bind to the VWA domain of Rpn10 and Rpn1 to enable proteasome-mediated proteolysis. Nat. Commun. **3**, 749 (2012). 22434192 10.1038/ncomms1752

[41] S. Elsasser, D. Chandler-Militello, B. Müller, J. Hanna, D. Finley, Rad23 and Rpn10 serve as alternative ubiquitin receptors for the proteasome. J. Biol. Chem. **279**, 26817–26822 (2004). 15117949 10.1074/jbc.M404020200

[42] R. Verma, R. Oania, J. Graumann, R. J. Deshaies, Multiubiquitin chain receptors define a layer of substrate selectivity in the ubiquitin-proteasome system. Cell **118**, 99–110 (2004). 15242647 10.1016/j.cell.2004.06.014

[43] L. Chen, U. Shinde, T. G. Ortolan, K. Madura, Ubiquitin-associated (UBA) domains in Rad23 bind ubiquitin and promote inhibition of multi-ubiquitin chain assembly. EMBO Rep. **2**, 933–938 (2001). 11571271 10.1093/embo-reports/kve203 PMC1084081

[44] E. Sakata , Localization of the proteasomal ubiquitin receptors Rpn10 and Rpn13 by electron cryomicroscopy. Proc. Natl. Acad. Sci. U.S.A. **109**, 1479–1484 (2012). 22215586 10.1073/pnas.1119394109 PMC3277190

[45] Q. Wang, P. Young, K. J. Walters, Structure of S5a bound to monoubiquitin provides a model for polyubiquitin recognition. J. Mol. Biol. **348**, 727–739 (2005). 15826667 10.1016/j.jmb.2005.03.007

[46] X. Chen , Cryo-EM reveals unanchored M1-ubiquitin chain binding at hRpn11 of the 26S proteasome. Structure **28**, 1206–1217.e1204 (2020). 32783951 10.1016/j.str.2020.07.011 PMC7642156

[47] M. E. Matyskiela, G. C. Lander, A. Martin, Conformational switching of the 26S proteasome enables substrate degradation. Nat. Struct. Mol. Biol. **20**, 781–788 (2013). 23770819 10.1038/nsmb.2616 PMC3712289

[48] Y. Dong , Cryo-EM structures and dynamics of substrate-engaged human 26S proteasome. Nature **565**, 49–55 (2019). 30479383 10.1038/s41586-018-0736-4 PMC6370054

[49] P. Śledź , Structure of the 26S proteasome with ATP-γS bound provides insights into the mechanism of nucleotide-dependent substrate translocation. Proc. Natl. Acad. Sci. U.S.A. **110**, 7264–7269 (2013). 23589842 10.1073/pnas.1305782110 PMC3645540

[50] L. Zhang, Y. Du, Q. Zheng, Structure basis of the phytoplasma effector SAP05 recognition specificities to plant Rpn10 in ubiquitin-independent protein degradation. bioRxiv [Preprint] (2023). 10.1101/2023.08.24.554544 (Accessed 13 September 2023).

[51] T. Du , Ubiquitin receptor PSMD4/Rpn10 is a novel therapeutic target in multiple myeloma. Blood **141**, 2599–2614 (2023). 36630605 10.1182/blood.2022017897 PMC10273170

[52] X. Lu , Structure-guided bifunctional molecules hit a DEUBAD-lacking hRpn13 species upregulated in multiple myeloma. Nat. Commun. **12**, 7318 (2021). 34916494 10.1038/s41467-021-27570-4 PMC8677766

[53] R. M. Benoit, R. N. Wilhelm, D. Scherer-Becker, C. Ostermeier, An improved method for fast, robust, and seamless integration of DNA fragments into multiple plasmids. Protein Expr. Purif. **45**, 66–71 (2006). 16289702 10.1016/j.pep.2005.09.022

[54] L. E. Bird , Application of in-fusion™ cloning for the parallel construction of *E. coli* expression vectors. Methods Mol. Biol. **1116**, 209–234 (2014). 24395367 10.1007/978-1-62703-764-8_15

[55] M. D. Nelson, D. H. Fitch, Overlap extension PCR: An efficient method for transgene construction. Methods Mol. Biol. **772**, 459–470 (2011). 22065455 10.1007/978-1-61779-228-1_27

[56] A. Dereeper , Phylogeny.fr: Robust phylogenetic analysis for the non-specialist. Nucleic Acids Res. **36**, W465–W469 (2008). 18424797 10.1093/nar/gkn180 PMC2447785

[57] K. Tamura, G. Stecher, S. Kumar, MEGA11: Molecular evolutionary genetics analysis version 11. Mol. Biol. Evol. **38**, 3022–3027 (2021). 33892491 10.1093/molbev/msab120 PMC8233496

[58] J. Jumper , Highly accurate protein structure prediction with AlphaFold. Nature **596**, 583–589 (2021). 34265844 10.1038/s41586-021-03819-2 PMC8371605

[59] S. McNicholas, E. Potterton, K. S. Wilson, M. E. Noble, Presenting your structures: The CCP4mg molecular-graphics software. Acta Crystallogr. D Biol. Crystallogr. **67**, 386–394 (2011). 21460457 10.1107/S0907444911007281 PMC3069754

[60] R. A. Laskowski , PDBsum: Structural summaries of PDB entries. Protein Sci. **27**, 129–134 (2018). 28875543 10.1002/pro.3289 PMC5734310

[61] W. Huang , Crystal structure of binary complex between Aster yellows witches’-broom phytoplasma effector SAP05 and the zinc finger domain of SPL5 from Arabidopsis thaliana. Protein Data Bank. 10.2210/pdb8pfc/pdb. Deposited 15 June 2023.

[62] W. Huang , Crystal structure of binary complex between Aster yellows witches’-broom phytoplasma effector SAP05 and the von Willebrand Factor Type A domain of the proteasomal ubiquitin receptor Rpn10 from Arabidopsis thaliana. Protein Data Bank. 10.2210/pdb8pfd/pdb. Deposited 15 June 2023.

