# Supplementary material for: Bimodular architecture of bacterial effector SAP05 that drives ubiquitin-independent targeted protein degradation
Source: Proc Natl Acad Sci U S A. 2023 Dec 1;120(49):e2310664120. doi: 10.1073/pnas.2310664120 (PMC10710061; doi:10.1073/pnas.2310664120)

PNAS

# Supporting Information for

# Bimodular Architecture of Bacterial Effector SAP05 that Drives Ubiquitin-Independent Targeted Protein Degradation

Qun Liu[a,1], Abbas Maqbool[b,c,1], Federico G. Mirkin[a], Yeshveer Singh[a], Clare E. M. Stevenson[b], David M. Lawson[b], Sophien Kamoun[c], Weijie Huang[d,*] and Saskia A. Hogenhout[a,*]

[a] Department of Crop Genetics, John Innes Centre, Norwich Research Park, Norwich NR4 7UH, United Kingdom; [b] Department of Biochemistry and Metabolism, John Innes Centre, Norwich Research Park, Norwich NR4 7UH, United Kingdom; [c] The Sainsbury Laboratory, University of East Anglia, Norwich NR4 7UH, United Kingdom; [d] National Key Laboratory of Plant Molecular Genetics, Shanghai Centre for Plant Stress Biology, Centre for Excellence in Molecular Plant Sciences, Chinese Academy of Sciences, Shanghai, China.

## This SI Appendix includes:


- Supplementary Material and Methods, pages 2-6.
- Supplementary Figures S1-S17, pages 7-28.
- Supplementary Tables S1-S2, pages 29-32.
- References, pages 33-34.
- Uncropped Gels and Western Blots, pages 35-46.


[1]These authors contributed equally to the work.

*To whom correspondence may be addressed. Email: wjhuang@cemps.ac.cn or saskia.hogenhout@jic.ac.uk.

## Materials and Methods

### Gene cloning, expression, and protein purification for crystallization and *in vitro* studies

For crystallization of SAP05 – SPL5$^{ZnF}$ complex, DNA encoding mature SAP05 excluding signal peptide (Ala33-Lys135) and DNA encoding the ZnF domain of SPL5 (Ser60-Leu127) were separately subcloned to pOPINF vector for N-terminal 6×His tags (1) using In-fusion cloning method (2, 3). The constructs were transformed individually into *E. coli* BL21 (DE3) competent cells. Bacterial cultures were grown in LB media (containing 50 μg/mL ampicillin) at 37 °C to an $OD_{600}$ around 0.5 followed by induction with 1 mM Isopropyl-b-D-thiogalactoside (IPTG) at 16 °C overnight with shaking at a speed of 220 rpm. Cell pellets were resuspended in buffer A1 (50 mM Tris-HCl, 50 mM glycine, 0.5 M NaCl, 20 mM imidazole, 5% glycerol, pH 8.0) followed by sonication lysis at 40% amplitude, 5 sec on / 10 sec off pulse for 30 min on ice. Cell debris was removed by centrifuge at 20000 g for 20 min. Purification of the proteins was performed using an ÄKTA Xpress purification system comprising initial capture with metal affinity chromatography (IMAC) using Ni-NTA column, which elutes proteins with buffer B1 (50 mM Tris-HCl, 50 mM glycine, 0.5 M NaCl, 0.5 M imidazole, 5% glycerol, pH 8.0), followed by gel filtration with the column Superdex 75 26/600 in buffer A4 (20 mM HEPES, 0.15 M NaCl, pH 7.5). Fractions with the elution peaks were assessed by SDS-PAGE gels, pooled and treated with HRV-3C protease overnight at 4°C. Afterwards, digested protein samples were passed through Ni-NTA column to remove cleaved 6×His tags. Untagged samples were assessed with SDS-PAGE gels, pooled and concentrated with 3 kDa cut-off vivaspin concentrators, followed by a second round of gel filtration with Superdex 75 26/600 in buffer A4. Eluted samples were assessed with SDS-PAGE gels. Afterwards, purified proteins were mixed together in equimolar ratio, followed by gel filtration chromatography. Eluted fractions were assessed by SDS-PAGE, pooled and concentrated to a final concentration of 15 mg/mL, for crystallization studies.

For crystallization of SAP05 – Rpn10$^{vWA}$ complex, the mature coding sequence of SAP05 (Ala33-Lys135) was cloned to pOPINF vector, Rpn10$^{vWA}$ (Val2-GLy193) was initially

cloned to pOPINM to make MBP-vWA fusion cassette. MBP-vWA was then amplified and ligated to pOPINA to remove the 6×His tag. The final constructs were co-transformed into *E. coli* BL21(DE3) competent cells. Proteins were co-expressed and co-purified using the IMAC and gel filtration as mentioned above. Eluted fractions from the complex were assessed from gel filtration peaks and SDS-PAGE. Afterwards, the eluted complex was subjected to HRV-3C protease cleavage overnight at 4°C followed by Ni-NTA column to remove tags. After a second round of gel filtration followed by assessment with SDS-PAGE, the purified complexes were pooled and concentrated to 15 mg/mL for crystallization studies.

For *in vitro* experiments, purified proteins were concentrated to ~10 mg/mL via 3 kDa cut-off vivaspin concentrators for subsequent analysis. Detection of proteins on SDS-PAGE gels was performed using Instant Blue staining solution (Abcam). Data of gel filtration traces were processed and plotted using ggplot2 in R (4).

**Protein crystallization, structure determination, and refinement**

Crystallization screens were set up in sitting-drop vapor diffusion format in MRC2 96-well crystallization plates with drops comprised of 0.3 µL precipitant solution and 0.3 µL of protein and incubated at 293 K. All crystals were cryoprotected in the crystallization solution supplemented with 25% (v/v) glycerol and mounted in Litholoops (Molecular Dimensions) before flash-cooling by plunging into liquid nitrogen. X-ray data were recorded on beamline I04 at the Diamond Light Source (Oxfordshire, UK) using an Eiger2 XE 16M hybrid photon counting detector (Dectris), with crystals maintained at 100 K by a Cryojet cryocooler (Oxford Instruments). Diffraction data were integrated and scaled using DIALS (5) via the XIA2 expert system (6), then merged using AIMLESS (7). The majority of the downstream analysis was performed through the CCP4i2 graphical user interface (8). Data collection statistics are summarized in Table 1.

The SAP05 – $SPL5^{ZnF}$ complex crystallized from 0.1 M MES pH 6.5, 25% (w/v) PEG 6000 in space group $P2_1$, with approximate cell parameters of a = 78.7, b = 165.0, c = 80.9 Å, $\beta$ = 109.7°. All X-ray data were collected from a single crystal, initially recorded at

a wavelength of 0.9795 Å (2 x 360° passes) and processed to 2.2 Å resolution, and then at a wavelength of 1.2770 Å (1 x 360° pass), this being close to the *K* X-ray absorption edge for zinc. The latter data set was processed to 2.4 Å resolution and enabled structure solution via the single-wavelength anomalous diffraction method using the CRANK2 pipeline (9), due to the presence of $Zn^{2+}$ ions bound to $SPL5^{ZnF}$. This produced a partial model corresponding to eight copies of a 1:1 complex of SAP05 – $SPL5^{ZnF}$ in the crystallographic asymmetric unit, giving an estimated solvent content of 59%, with each copy of $SPL5^{ZnF}$ containing two $Zn^{2+}$ ions. The model was completed through several iterations of model building in COOT (10) and restrained refinement in REFMAC5 (11) against the higher resolution data set at 2.2 Å resolution. Refinement and validation statistics are summarized in Table 1.

The SAP05 – $Rpn10^{vWA}$ complex crystallized from 0.1 M Sodium HEPES pH 7.5, 10.7 % (w/v) PEG 4000 in space group $P2_1$, with approximate cell parameters of a = 42.4, b = 68.6, c = 49.9 Å, $\beta$ = 92.8°. X-ray data were recorded from a single crystal at a wavelength of 0.9796 Å (2 x 360° passes) and processed to 2.17 Å resolution. Analysis of the likely composition of the asymmetric unit suggested that it contained one copy of a 1:1 complex of SAP05 – $Rpn10^{vWA}$, giving an estimated solvent content of 44%. The structure was solved via molecular replacement using PHASER (12). One copy of SAP05 was taken from the above SAP05 – $SPL5^{ZnF}$ complex as the first template, and the second template was derived from a homology model of $Rpn10^{vWA}$ produced by Swiss-Model (13) and based on PDB entry 5VFT (14). The model was completed through several iterations of model building in COOT and restrained refinement in REFMAC5. Refinement and validation statistics are summarized in Table 1. All structural figures were prepared using CCP4mg (15). Distances between residues were measured with COOT (10).

**Yeast two-hybrid assay (Y2H)**

The coding sequences of SAP05 or SAP05 mutants excluding signal peptides were amplified and ligated into Gateway vector pDEST-GBKT7 (BD). Full length sequences of *A. thaliana* Rpn10, two SPLs (SPL11 and SPL15), and three GATAs (GATA18, GATA19 and GATA25) were amplified and cloned into vector pDEST-GADKT7 (AD) using

Gateway cloning methods (16, 17). Constructs used to test protein-protein interactions were co-transformed into the yeast *Saccharomyces cerevisiae* strain AH109 using the Matchmaker Gold yeast two-hybrid system (Clontech). Empty pDEST-GBKT7 and pDEST-GADKT7 vectors were used as negative control. Yeast growth was assessed on solid double dropout medium lacking leucine and tryptophan (SD-LW) which indicates the presence of both AD and BD constructs. Interactions between AD and BD fusion proteins were screened on selective dropout medium; triple dropout medium lacking leucine, tryptophan and histidine (SD-LWH) or with the addition of 3-amino-1,2,4-triazole (3-AT) at a final concentration of 10 mM when self-activation was observed or to improve selection stringency, and quadruple dropout medium lacking leucine, tryptophan, histidine and adenine (SD-LWHA). Yeast plates were kept in 28°C incubators for 5 days before imaging.

**Degradation assay in *N. benthamiana***

The coding sequences of SAP05 or SAP05 mutants (excluding signal peptides) were amplified and ligated into Gateway vector pB7WG2. Full length coding sequence of AtSPL5 were amplified and tagged with 3×HA at N-terminal, then ligated into Gateway vector pB7WG2. After sequencing, the constructs were separately transformed to *Agrobacterium tumefaciens* strain GV3101 and plated on LB solid medium containing rifampicin, gentamicin and spectinomycin and grown at 28 °C for 24-48 h. Then colonies were picked and checked by PCR using plasmids extracted from overnight liquid culture and gene-specific primers. Positive colonies were grown at 28 °C overnight, harvested, and resuspended in infiltration buffer (10 mM $MgCl_2$, 10 mM MES, pH 5.6), supplemented with 100 µM acetosyringone. Appropriate combinations of above constructs together with pCB301-P19 were mixed at an $OD_{600}$ of 0.5 per construct and infiltrated to the abaxial surface of 4-week-old *N. benthamiana* leaves using 1 ml needleless syringe. Infiltrations were conducted on randomly selected leaves, with triplicates consisting of three leaves each. To minimize variation, each single leaf was infiltrated with four combinations: SAP05 only, one SAP05 mutant only, SAP05 plus HA-SPL5, and SAP05 mutant plus HA-SPL5. After 3 days post-infiltration, the infiltrated leaves were detached, and the infiltrated areas were harvested for total protein extraction.

To detect SPL5 and SAP05, western blots were performed as follows. Total protein extracts were separated on 4-20% gradient gels (Bio-Rad) and transferred to 0.22 µm PVDF membranes using the Bio-Rad mini-PROTEAN Electrophoresis system. Protein loading was visualized with Ponceau S staining solution (Thermo Scientific) and washed off with water. Membranes were then blocked by 5% (w/v) milk powder in Tris-buffered saline (TBS) and 0.1% (v/v) Tween-20 for 2 h at room temperature. Membranes were then overnight incubated at 4°C with the anti-HA primary antibody (OptimAb HA. 11, Eurogentec), which was raised from mouse serum, at a ratio of 1:2000 dilution. Afterwards, the membrane was probed with Alkaline-Phosphatase-conjugated anti-mouse secondary antibody (Thermo Scientific) for 1 h at room temperature and imaged after incubation with NBT/BCIP substrate solution (Thermo Scientific). Membranes were washed after detection, blocked in the same conditions as previously described and incubated overnight at 4°C with rabbit raised anti-SAP05 antibody at a 1:5000 ratio (18). The membrane was then probed with HRP conjugated anti-rabbit secondary antibody (Sigma) and imaged with Immobilon Western Chemiluminescent HRP Substrate (Sigma).

**Degradation assay in *A. thaliana* protoplast**

*A. thaliana* (Col-0) mesophyll protoplast isolation and transformation were carried out as previously reported (19). Briefly, protoplasts from the mesophyll cells were isolated from leaves of 3-4 week-old plants which were grown under short-day (10 h light/14 h dark) conditions at 22°C. For transfection, 300 µL of fresh protoplast solution (400,000/mL) was co-transformed with 12 µg high quality plasmids for each construct using PEG-calcium method. Transfected protoplasts were incubated at 22°C for 16 h in dark. Following which, total protein was extracted and examined via western blots using HRP-conjugated anti-mouse secondary antibody (Sigma). Protein loading was visualized with Amido Black Staining Solution (Sigma).

A

**SAP05 (12.3 kDa):**

MFKIKNNLLKSKIFVFILLGLFVIINNHQAMAAPNEEFVGDMRIVNVNLSNIDILKKHETFKKYFDFTLTGPRYNGNIAEFAMI
WKIKNPPLNLLGVFFDDGTRDDEDDKYILEELKQIGNGAKNMYIFWQYEQK

**SPL5$^{ZnF}$ (8.1 kDa):**

MEGQRTQRRGYLKDKATVSNLVEEEMENGMDGEEEDGGDEDKRKKVMERVRGPSTDRVPSRLCQVDRCTVNLTEAK
QYYRRHRVCEVHAKASAATVAGVRQRFCQQCSRFHELPEFDEAKRSCRRRLAGHNERRRKISGDSFGEGSGRRGFS
GQLIQTQERNRVDRKLPMTNSSFKRPQIR

**Rpn10$^{vWA}$ (20.7 kDa):**

MVLEATMICIDNSEWMRNGDYSPSRLQAQTEAVNLLCGAKTQSNPENTVGILTMAGKGVRVLTTPTSDLGKILACMHGL
DVGGEINLTAAIQIAQLALKHRQNKNQRQRIIVFAGSPIKYEKKALEIVGKRLKKNSVSLDIVNFGEDDDEEKPQKLEALLTA
VNNNDGSHIVHVPSGANALSDVLLSTPVFTGDEGASGYVSAAAAAAAAGGDFDFGVDPNIDPELALALRVSMEEERARQ
EAAAKKAADEAGQKDKDGDTASASQETVARTTDKNAEPMDEDSALLDQAIAMSVGDVNMSEAADEDQDLALALQMSM
SGEESSEATGAGNNLLGNQAFISSVLSSLPGVDPNDPAVKELLASLPDESKRTEEEESSSKKGEDEKK

B

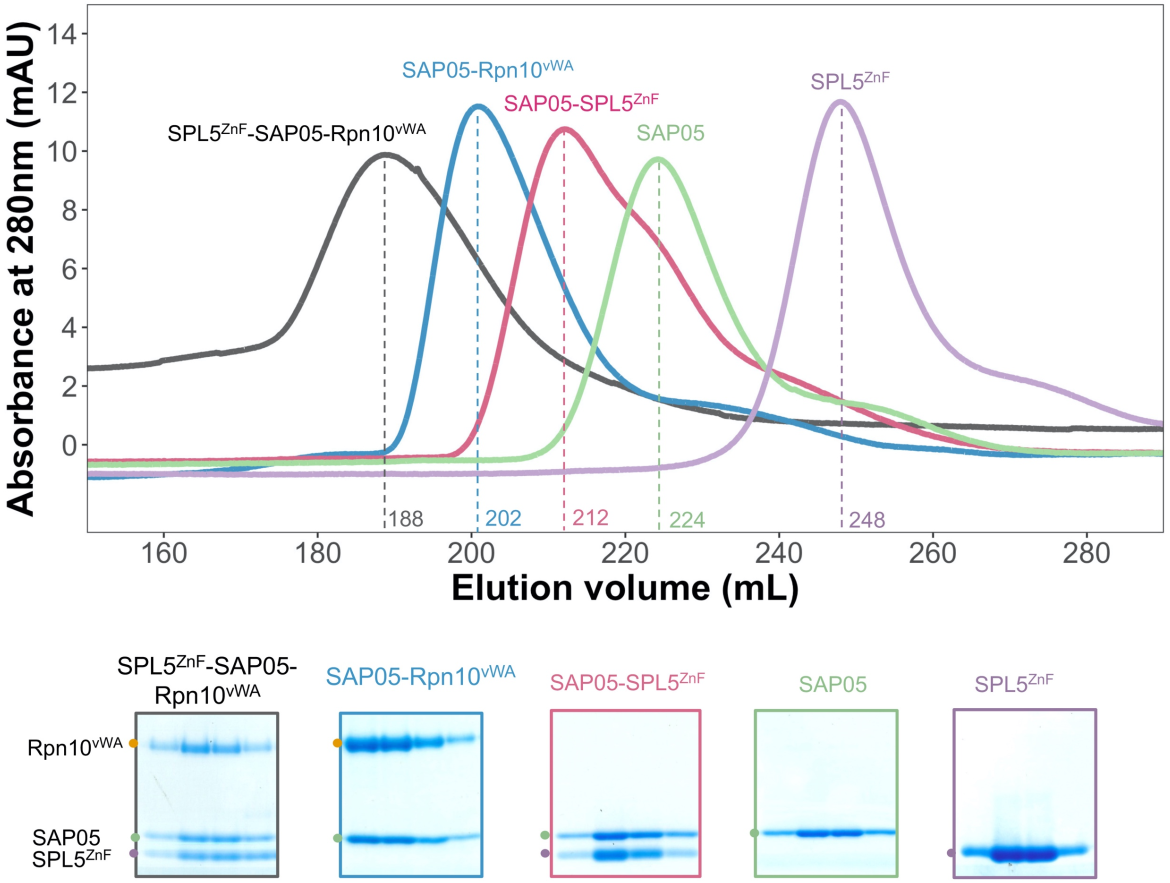


**Fig. S1. Expression and purification of SAP05 – SPL5$^{ZnF}$ and SAP05 – Rpn10$^{vWA}$ complexes for crystallization.** (A) The amino acid sequences of mature SAP05, SPL5$^{ZnF}$, and Rpn10$^{vWA}$ utilized for protein expression are highlighted in yellow on full-length proteins and their molecular weights are indicated in brackets. (B) Gel filtration chromatogram (top) and SDS-PAGE (bottom) analyses of peak fractions of purified

SAP05, SPL5$^{ZnF}$, SAP05 – SPL5$^{ZnF}$, SAP05 – Rpn10$^{vWA}$ and SPL5$^{ZnF}$ – SAP05 – Rpn10$^{vWA}$ complexes. SDS-PAGE gels are framed with the same color as the corresponding elution peak in gel filtration chromatogram. Yellow, green and purple dots indicate the expected size of Rpn10$^{vWA}$, SAP05 and SPL5$^{ZnF}$, respectively.

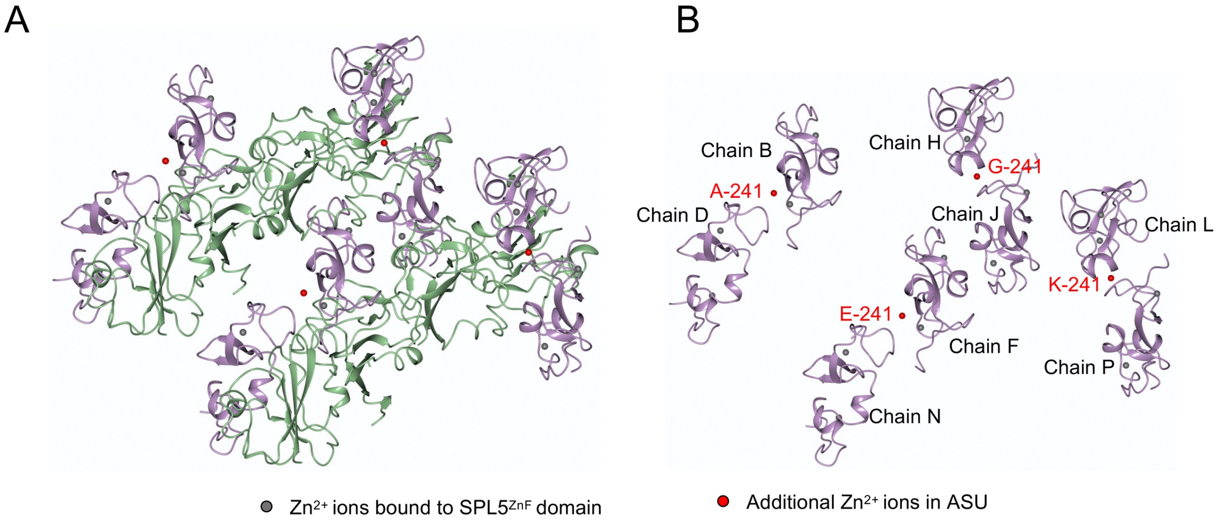


**Fig. S2. Position of $Zn^{2+}$ ions in asymmetric unit (ASU) in the SAP05 – $SPL5^{ZnF}$ crystal.** (A) Eight copies of SAP05 – $SPL5^{ZnF}$ complex in ASU are shown as cartoon (SAP05 colored green and $SPL5^{ZnF}$ colored purple). (B) Eight copies of $SPL5^{ZnF}$ in ASU are shown as cartoon (purple). The chain IDs are labelled. Structural $Zn^{2+}$ ions within each $SPL5^{ZnF}$ domain are shown as grey spheres, and four additional $Zn^{2+}$ ions are shown as red spheres.

A

SAP05 and $SPL5^{ZnF}$ interface

| Residues on SAP05 | Residues on $SPL5^{ZnF}$ | Mutated residues on SAP05 |
|---|---|---|
| D66 | Q77 | D66A, D66E |
| G76 | Q105 | G76W |
| N77 | Y78, Q105, S108 | N77A, N77D, N77R |
| E80 | R81 | E80A, E80R |
| R104 | Q105 | - |
| D106 | K90, R121 | D106A, D106R |

B

SAP05 and $Rpn10^{vWA}$ interface

| Residues on SAP05 | Residues on $Rpn10^{vWA}$ | Mutated residues on SAP05 |
|---|---|---|
| R43 | D68 | R43A, R43E |
| N48 | Q27 | - |
| S50 | E31 | S50A |
| H58 | N34 | H58A, H58W |
| T60 | G38, Q42 | T60W |
| N125 | D68 | N125A, N125D |
| Y127 | G70 | Y127W |
| Y132* | S24* | - |

**Fig. S3. Residues on SAP05 – $SPL5^{ZnF}$ and SAP05 – $Rpn10^{vWA}$ interfaces and the mutations generated on SAP05.** Interacting residues and generated SAP05 mutations on SAP05 – $SPL5^{ZnF}$ (A) and SAP05 – $Rpn10^{vWA}$ (B) interaction interface. Interactions were analyzed with CCP4mg. *The electron density for $Rpn10^{vWA}$ S24 is suboptimal, implying uncertainty regarding its precise placement and proximity for its potential interaction with SAP05 Y132.

A

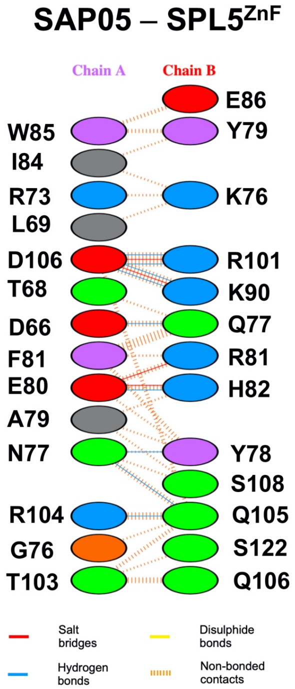


B

Distance of some interacting residues between SAP05 and SPL5$^{ZnF}$

| Residues on SAP05 | Residues on SPL5$^{ZnF}$ | Distance (Å) |
|---|---|---|
| D66 | Q77 | 2.67 |
| G76 | Q105 | 3.53 |
| N77 | Y78 | 3.07 |
| N77 | Q105 | 2.76 |
| N77 | S108 | 3.13 |
| E80 | R81 | 2.86, 3.23 |
| R104 | Q105 | 2.63 |
| D106 | K90 | 2.73 |
| D106 | R121 | 2.80, 2.83 |

C

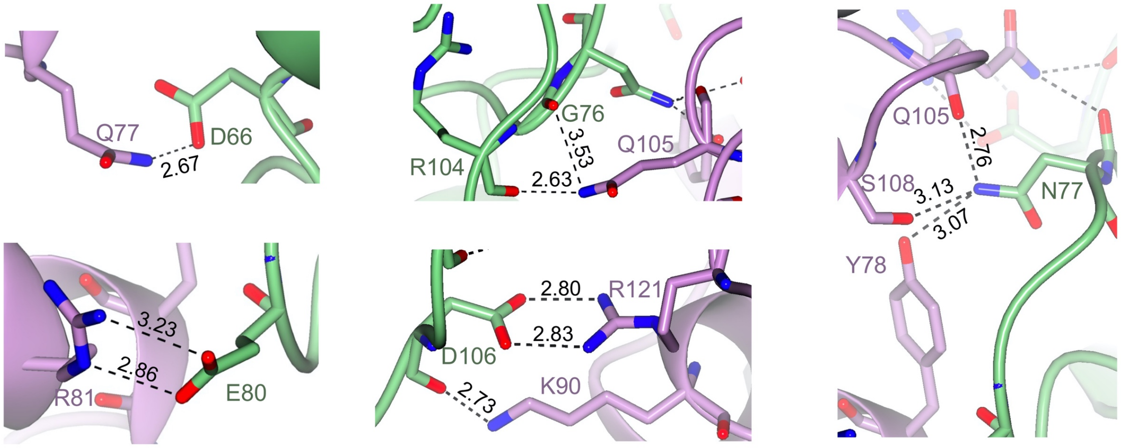


**Fig. S4. Analysis of interacting residues on SAP05 – SPL5$^{ZnF}$ interface.** (A) Different types of interaction between residues on SAP05 and SPL5$^{ZnF}$ by PDBsum (https://www.ebi.ac.uk/thornton-srv/databases/cgi-bin/pdbsum/GetPage.pl?pdbcode=8pfc&template=interfaces.html&o=RESIDUE&l=1). (B) Distances of important interacting residues measured with Coot. (C) Distances between of interacting residues shown in SAP05 – SPL5$^{ZnF}$ structure.

| Cell | | Syringe | | T (°C ) | N | $K_d$ ($M^{-6}$) | ΔH ($kcal·mol^{-1}$) | ΔG ($kcal·mol^{-1}$) |
|---|---|---|---|---|---|---|---|---|
| Contents | Conc. (μM) | Contents | Conc. (μM) | | | | | |
| $SPL5^{ZnF}$ | 20 | SAP05 | 200 | 25 | 1.15 | 0.510 | -14.4 ± 0.919 | -8.59 |
| $SPL5^{ZnF}$ | 20 | SAP05 | 200 | 25 | 1.05 | 0.388 | -11.9 ± 1.29 | -8.57 |
| $SPL5^{ZnF}$ | 20 | SAP05 | 200 | 25 | 1.01 | 0.445 | -18.1 ± 1.45 | -8.67 |
| SAP05 | 20 | $SPL5^{ZnF}$ | 250 | 25 | 0.942 | 0.458 | -14.9 ± 0.998 | -8.65 |
| SAP05 | 20 | $SPL5^{ZnF}$ | 250 | 25 | 0.835 | 0.560 | -14.3 ± 0.435 | -8.53 |
| SAP05 | 20 | $SPL5^{ZnF}$ | 250 | 25 | 0.914 | 0.541 | -14.3 ± 0.425 | -8.55 |
| SAP05-$Rpn10^{vWA}$ | 20 | $SPL5^{ZnF}$ | 200 | 25 | 0.995 | 0.683 | -10.8 ± 0.164 | -8.41 |
| SAP05-$Rpn10^{vWA}$ | 20 | $SPL5^{ZnF}$ | 200 | 25 | 1.06 | 0.620 | -7.87 ± 0.754 | -8.47 |
| $SAP05^{D66A}$ | 20 | $SPL5^{ZnF}$ | 200 | 25 | n.b. | n.b. | n.b. | n.b. |
| $SAP05^{D66A}$ | 20 | $SPL5^{ZnF}$ | 200 | 25 | n.b. | n.b. | n.b. | n.b. |
| $SAP05^{D66A}$ | 20 | $SPL5^{ZnF}$ | 200 | 25 | n.b. | n.b. | n.b. | n.b. |
| $SAP05^{D66E}$ | 30 | $SPL5^{ZnF}$ | 500 | 25 | 1.11 | 15.8 | -7.32 ± 3.62 | -6.55 |
| $SAP05^{D66E}$ | 30 | $SPL5^{ZnF}$ | 500 | 25 | 1.22 | 19.6 | -7.30 ± 1.74 | -6.42 |
| $SAP05^{N77R}$ | 20 | $SPL5^{ZnF}$ | 200 | 25 | n.b. | n.b. | n.b. | n.b. |
| $SAP05^{N77R}$ | 20 | $SPL5^{ZnF}$ | 200 | 25 | n.b. | n.b. | n.b. | n.b. |
| $SAP05^{N77R}$ | 20 | $SPL5^{ZnF}$ | 200 | 25 | n.b. | n.b. | n.b. | n.b. |
| $SAP05^{H58A-T60W}$ | 20 | $SPL5^{ZnF}$ | 300 | 25 | 0.967 | 2.69 | -5.63 ± 0.173 | -7.60 |
| $SAP05^{H58A-T60W}$ | 20 | $SPL5^{ZnF}$ | 300 | 25 | 1.00 | 4.247 | -6.18 ± 0.338 | -7.33 |
| $SAP05^{H58A-T60W}$ | 20 | $SPL5^{ZnF}$ | 300 | 25 | 1.08 | 3.85 | -5.76 ± 0.201 | -7.39 |

**Fig. S5. Thermodynamic parameters obtained from ITC binding experiments.** Two or three repeats of ITC tests by titrating SAP05 to $SPL5^{ZnF}$ or $SPL5^{ZnF}$ to SAP05-$Rpn10^{vWA}$ complex or different SAP05 mutants. Conc., concentration. n.b., not binding.

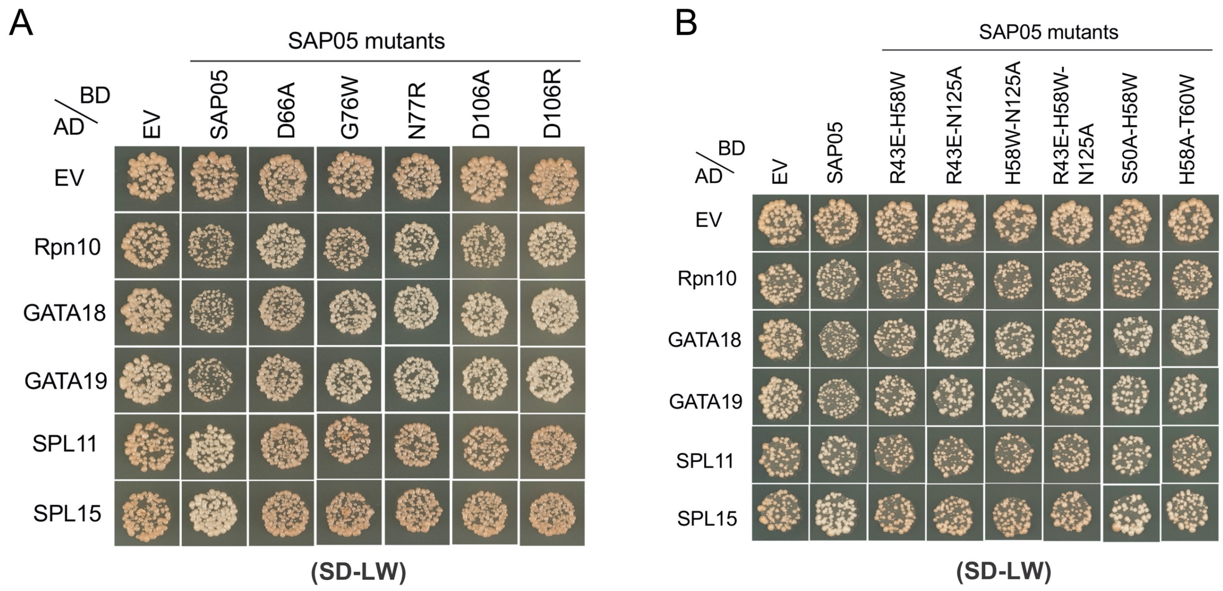


**Fig. S6. Yeast transformation controls for Y2H assays in the study, related to Figures 2 and 3.** (A) Yeast growth on double dropout medium lacking leucine and tryptophan indicating the expression of AD and BD constructs in Y2H assays for Figure 2E (A) and Figure 3C (B) in the main text.

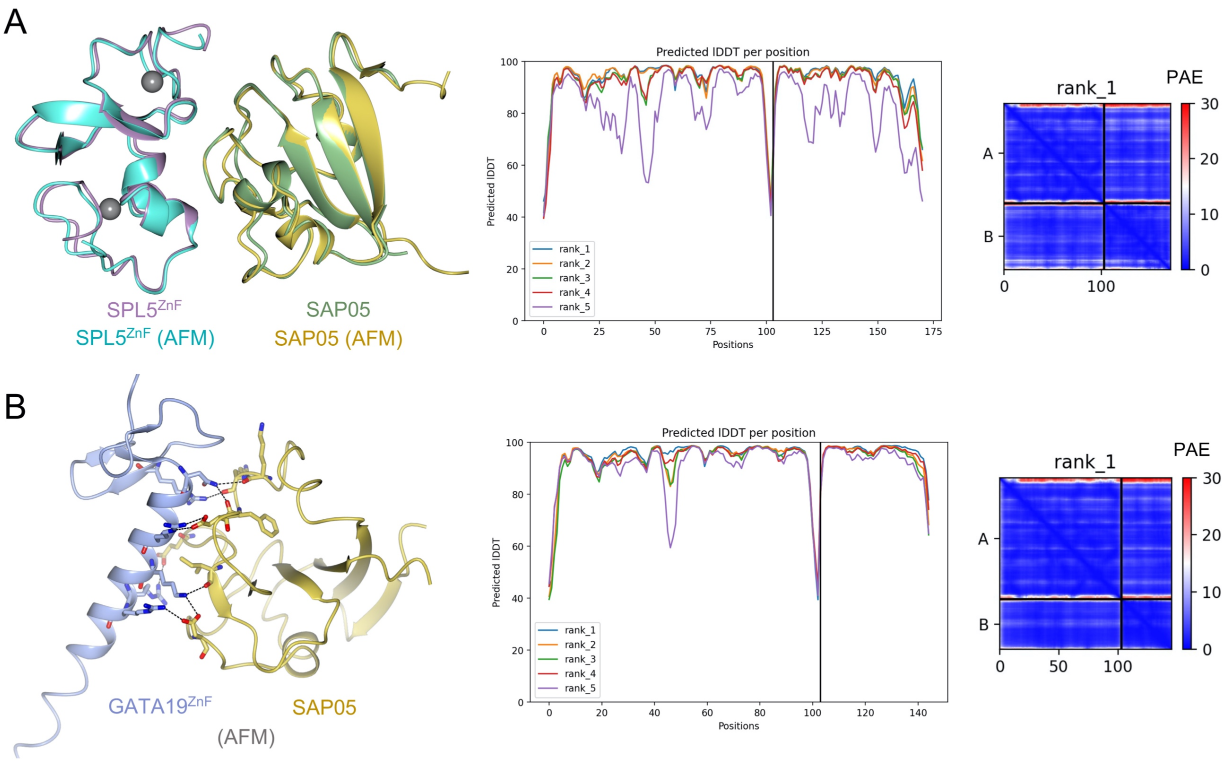


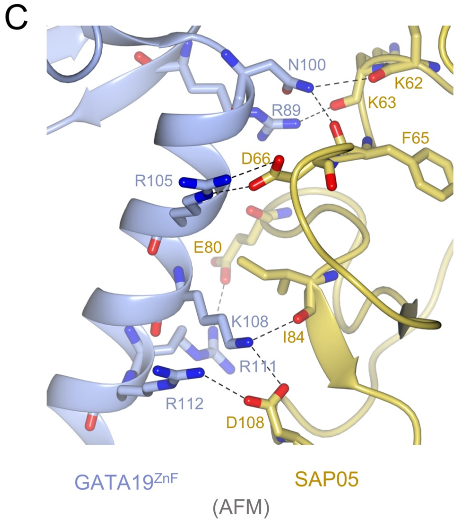


Interacting residues between SAP05 and GATA19$^{ZnF}$

| Residues on SAP05 | Residues on GATA19$^{ZnF}$ |
|---|---|
| K62 | N100 |
| K63 | R89 |
| F65 | N100 |
| D66 | R105 |
| E80 | R111 |
| I84 | K108 |
| D108 | K108, R112 |

**Fig. S7. Structural prediction of SAP05 – SPL5$^{ZnF}$ and SAP05 – GATA19$^{ZnF}$ complexes.** (A) Left, AlphaFold model (AFM) for SAP05 – SPL5$^{ZnF}$ complex (yellow – cyan) superimposed on the crystal structure of SAP05 – SPL5$^{ZnF}$ complex (green – purple). Middle, the predicted local Distance Difference Test (lDDT) value of SAP05 – SPL5$^{ZnF}$ model, showing the quality of predicted models by evaluating local distance differences to the reference. Best model (rank_1) was used for superimposition. Right, predicted aligned error (PAE) of the rank_1 model, showing the estimate of position error

between predicted and true structures. Blue means lower error scores, red means higher error scores. (B) Left, AFM model (rank_1) of SAP05 – GATA19$^{ZnF}$ complex (yellow – light blue). Middle, predicted lDDT value of SAP05 – GATA19$^{ZnF}$ complex. Right, predicted aligned error (PAE) of the rank_1 model. (C) Left, enlarged view for predicted SAP05 – GATA19$^{ZnF}$ interaction interface. SAP05 residues and GATA19$^{ZnF}$ residues that involved for interaction with GATA19$^{ZnF}$ were labelled in yellow and light blue, respectively. Dashed line shows predicted interactions between SAP05 and GATA19$^{ZnF}$ from AFM model. Right, interacting residues between SAP05 and GATA19$^{ZnF}$ of predicted structure.

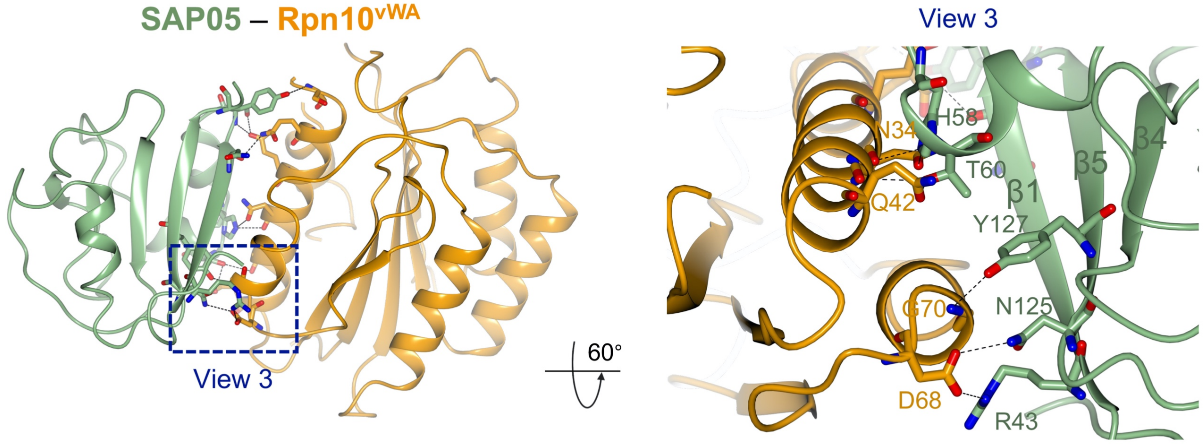


**Fig. S8. Another view of SAP05 – Rpn10$^{vWA}$ interaction.** Left, the overview of the interacting interface with the dashed square displaying the areas for enlarged view. Right, the enlarged and rotated view 3 of the lower part of the interface.

A

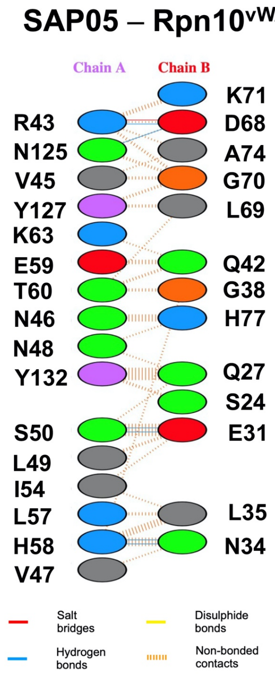


B

Distance of some interacting residues between SAP05 and Rpn10$^{vWA}$

| Residues on SAP05 | Residues on Rpn10$^{vWA}$ | Distance (Å) |
|---|---|---|
| R43 | D68 | 3.10 |
| N48 | Q27 | 3.54 |
| S50 | E31 | 2.48, 2.57 |
| H58 | N34 | 2.86, 3.08 |
| T60 | G38 | 3.76 |
| T60 | Q42 | 3.67 |
| N125 | D68 | 3.23 |
| Y127 | G70 | 3.26 |
| Y132* | S24* | 3.58* |

C

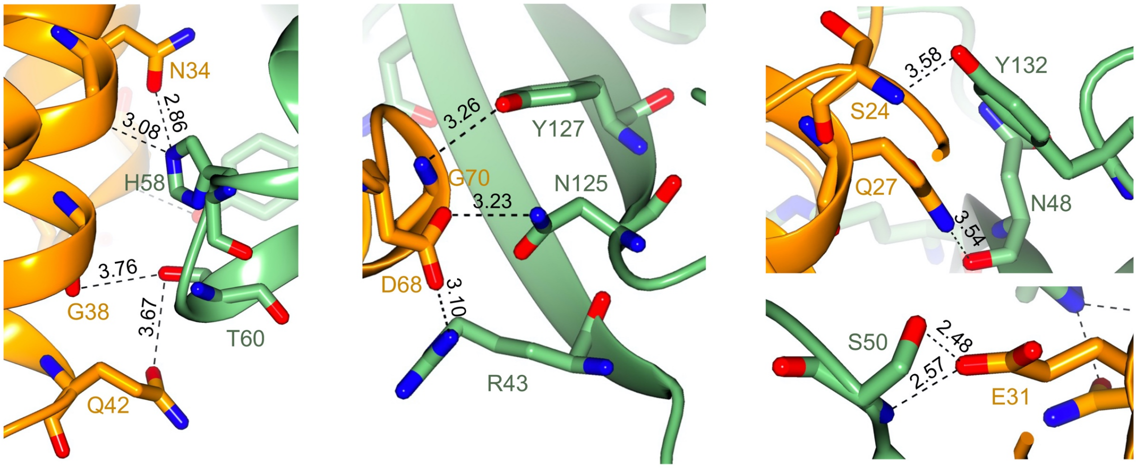


**Fig. S9. Analysis of interacting residues on SAP05 – Rpn10$^{vWA}$ interface.** (A) Different types of interaction between residues on SAP05 and Rpn10$^{vWA}$ by PDBsum (https://www.ebi.ac.uk/thornton-srv/databases/cgi-bin/pdbsum/GetPage.pl?pdbcode=8pfd&template=interfaces.html&o=RESIDUE&l=1). (B) Distances of important interacting residues measured with Coot. (C) Distances between interacting residues shown on SAP05-Rpn10$^{vWA}$ structure. *The electron density for Rpn10$^{vWA}$ S24 is suboptimal, implying uncertainty regarding its precise placement and proximity for its potential interaction with SAP05 Y132..

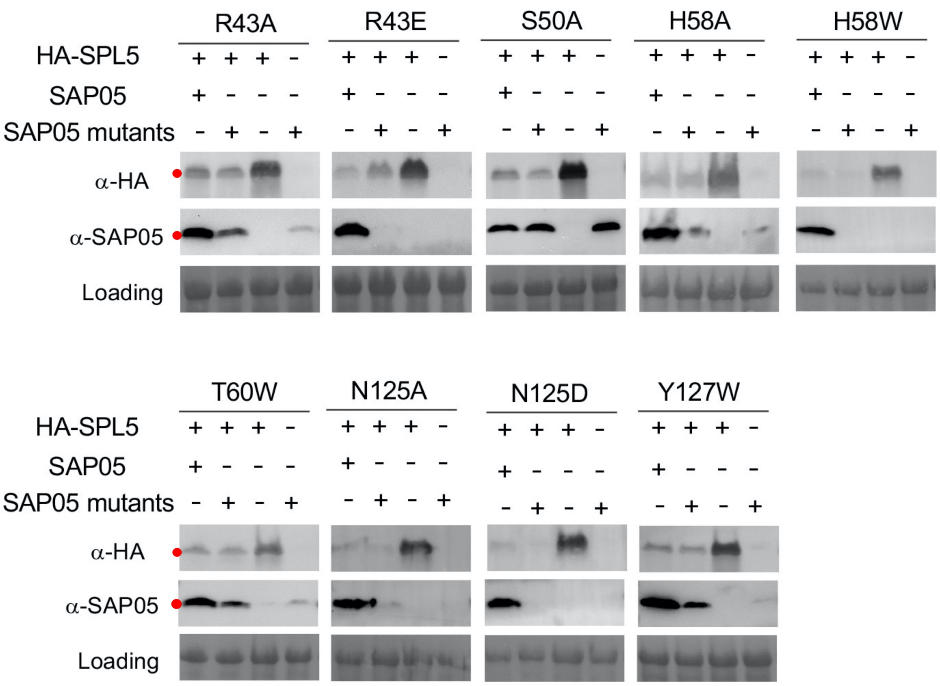


**Fig. S10. Western blot analysis of proteasomal degradation of SPL5 in presence of SAP05 wild-type or single mutants on sheet surface.** Red dots on the blots indicate the expected sizes of the transiently expressed proteins in *N. benthamiana* leaves. Protein loading was visualized using Ponceau S staining. HA, Hemagglutinin.

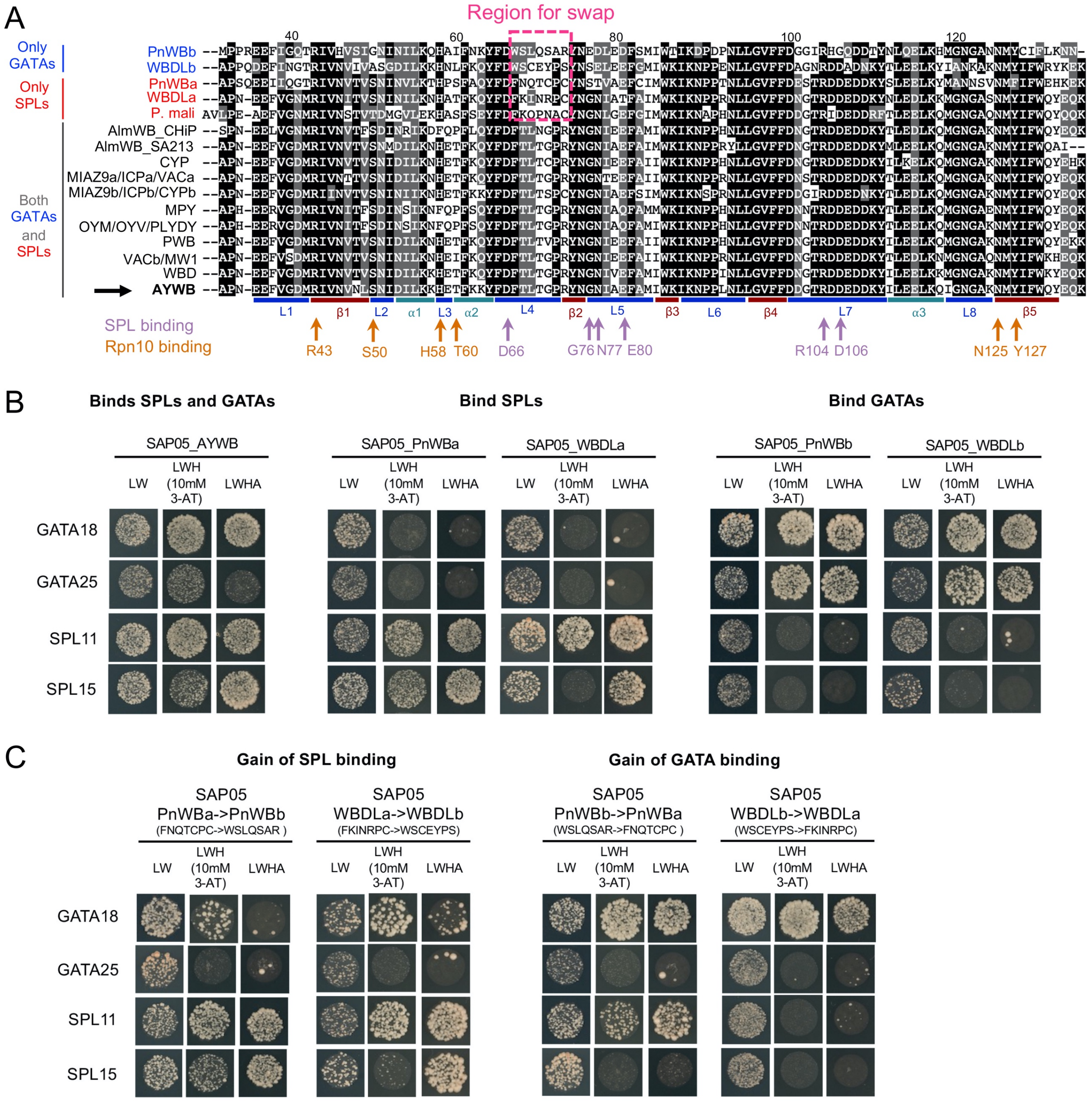


**Fig. S11. Conservation of SPL, GATA and Rpn10-interacting residues in SAP05 homologs.** (A) Multiple sequence alignment of SAP05 homologs from different phytoplasma species showing that the Rpn10-binding or SPL binding residues are well conserved. Identical and conserved amino acid residues are denoted by black and gray backgrounds, respectively. Homologs binding only GATAs, only SPLs, and both GATAs and SPLs are displayed in blue, red and black, respectively. The secondary structure of AYWB SAP05 is highlighted at the bottom. SAP05 residues involved in SPL binding (purple) or Rpn10 binding (orange) are pointed with arrows. The region used for swap in

(C) is marked with pink dashed rectangle. (B) Y2H assay showing SAP05 homologs that specifically bind SPLs or GATAs. (C) Y2H assay showing that swapping loop 4 sequences from GATA-binding SAP05 homologs contributes to SPL binding, and swapping loop 4 sequences from SPL-binding SAP05 homologs leads to GATA binding.

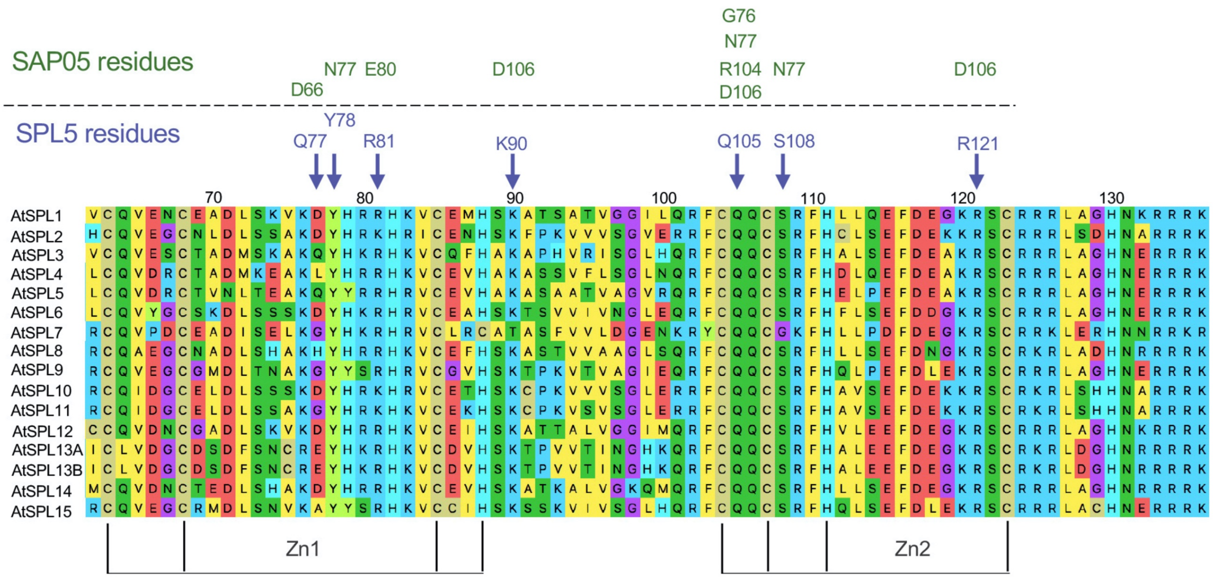


**Fig. S12. Multiple sequence alignment of ZnF domains of *A. thaliana* SPLs using MUSCLE.** The conserved SPL residues involved in the interaction with SAP05 are indicated with purple arrows and the corresponding SAP05 residues are shown in green. Accession numbers for AtSPLs are: SPL1, AT2G47070; SPL2, AT5G43270; SPL3, AT2G33810; SPL4, AT1G53160; SPL5, AT3G15270; SPL6, AT1G69170; SPL7, AT5G18830; SPL8, AT1G02065; SPL9, AT2G42200; SPL10, AT1G27370; SPL11, AT1G27360; SPL12, AT3G60030; SPL13A, AT5G50570; SPL13B, AT5G50670; SPL14, AT1G20980; SPL15, AT3G57920.

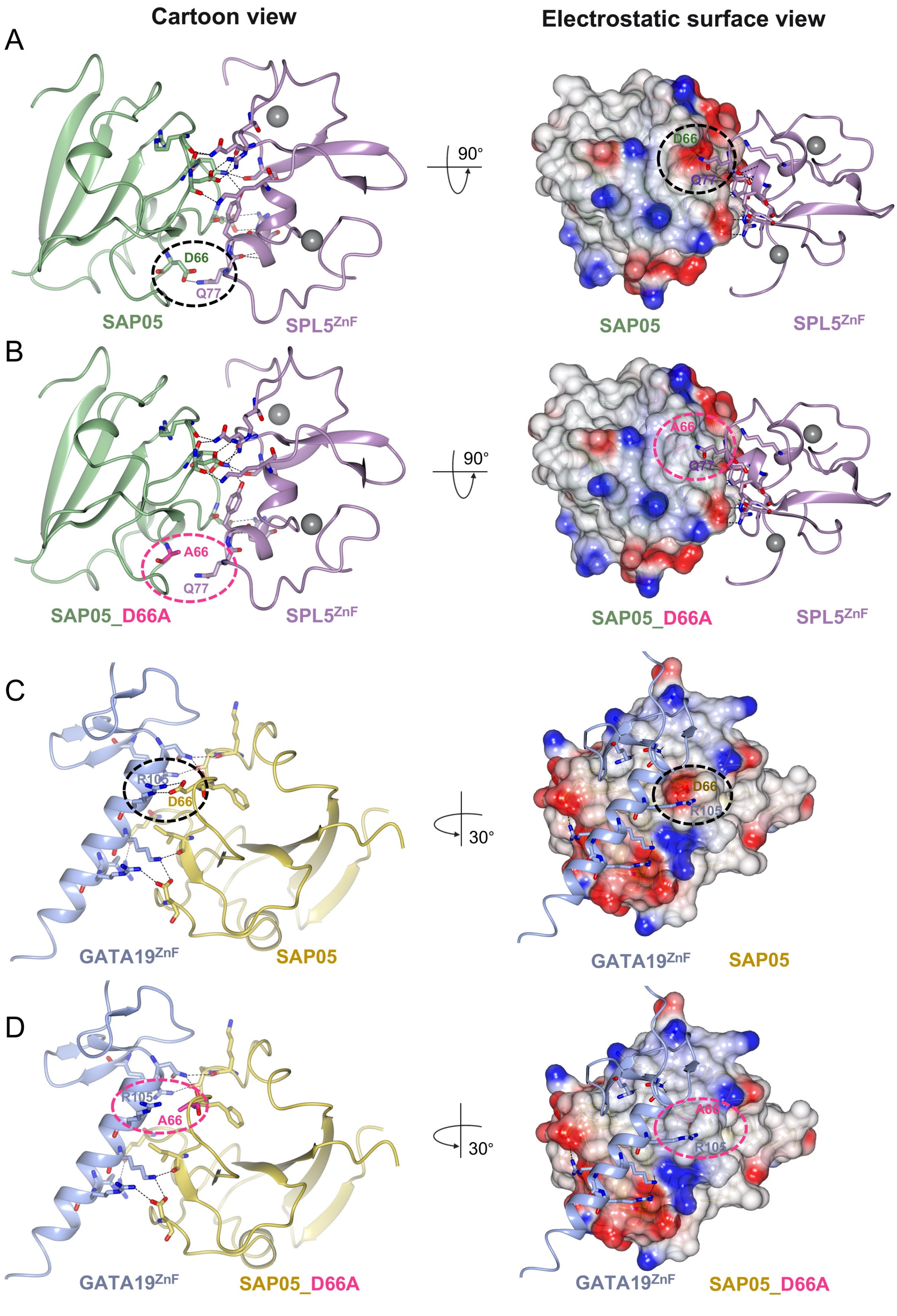

Cartoon view
Electrostatic surface view
A
D66
Q77
SAP05
SPL5ZnF
90°
D66
Q77
SAP05
SPL5ZnF
B
A66
Q77
SAP05_D66A
SPL5ZnF
90°
A66
Q77
SAP05_D66A
SPL5ZnF
C
R105
D66
GATA19ZnF
SAP05
30°
D66
R105
GATA19ZnF
SAP05
D
R105
A66
GATA19ZnF
SAP05_D66A
30°
A66
R105
GATA19ZnF
SAP05_D66A

**Fig. S13. SAP05 D66 creates an electronegative surface at the binding interface with zinc-fingers of SPL and GATA.** (A) SAP05 D66 interacts with Q77 on $SPL5^{ZnF}$ (left) and present negative surface on the binding interface (right). (B) SAP05 D66A lost the interaction with SPL5 Q77 (left), and the electronegativity on the interaction surface is reduced (right). (C) SAP05 D66 interacts with $GATA19^{ZnF}$ R105 based on AlphaFold predicted top-rank structure (left) and present negative surface at the interface (right). (D) SAP05 D66A lost the interaction with GATA19 R105 (left), and the electronegativity on the interaction surface is reduced (right).

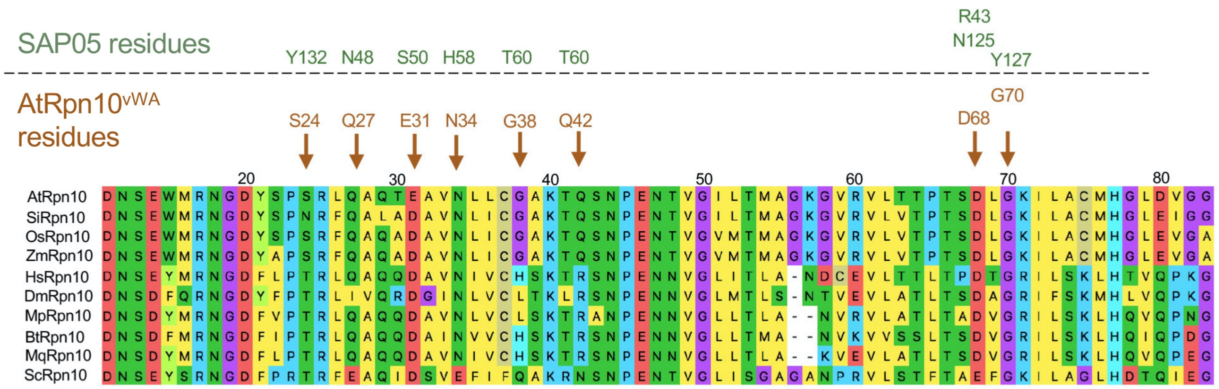


**Fig. S14. Multiple sequence alignment of Rpn10$^{vWA}$ from different species.** The conserved Rpn10$^{vWA}$ residues involved in interaction with SAP05 are indicated with orange arrows, and the corresponding SAP05 residues are shown on top in green. Sequence alignment was conducted with MUSCLE in MEGA11 software. AtRpn10, *Arabidopsis thaliana* Rpn10 (Uniprot ID: P55034); SlRpn10, *Solanum lycopersicum* Rpn10 (Uniprot ID: A0A3Q7F6N7); OsRpn10, *Oryza sativa* Rpn10 (Uniprot ID: O82143); ZmRpn10, *Zea mays* Rpn10 (Uniprot ID: B6TK61); HsRpn10, *Homo sapiens* Rpn10 (Uniprot ID: Q5VWC4); DmRpn10, *Drosophila melanogaster* Rpn10 (Uniprot ID: P55035); MpRpn10, *Myzus persicae* Rpn 10 (GenBank: XP_022181722.1); BtRpn10, *Bemisia tabaci* Rpn10 (GenBank: XP_018915695); MqRpn10, *Macrosteles quadrilineatus* Rpn10; ScRpn10, *Saccharomyces cerevisiae* Rpn10 (Uniprot ID: P38886).

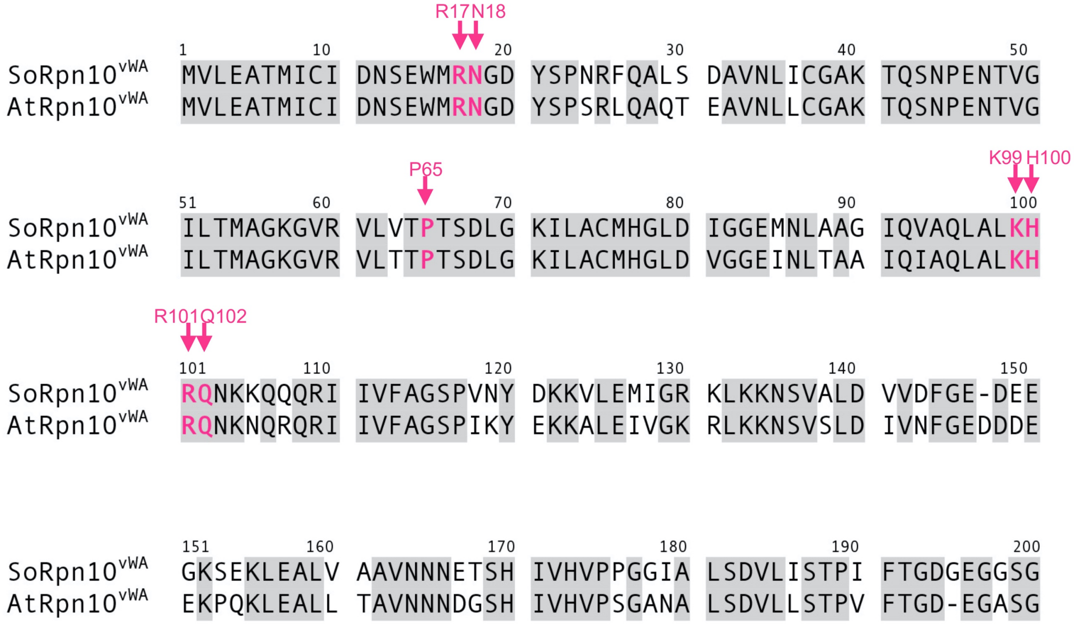


**Fig. S15. Sequence alignment of Rpn10 vWA domains from *Spinacia oleracea* and *A. thaliana.*** The conserved vWA residues involved in interaction with SAP05 are marked with grey background. Residues interacting with 26S proteasome are marked pink with pink arrows. SoRpn10$^{vWA}$ sequence was extracted from the structure of spinach 19S proteasome (PDB 8AMZ). Sequence alignment was conducted with MUSCLE in MEGA11 software and residues interacting with 26S proteasome were analyzed with CCP4mg.

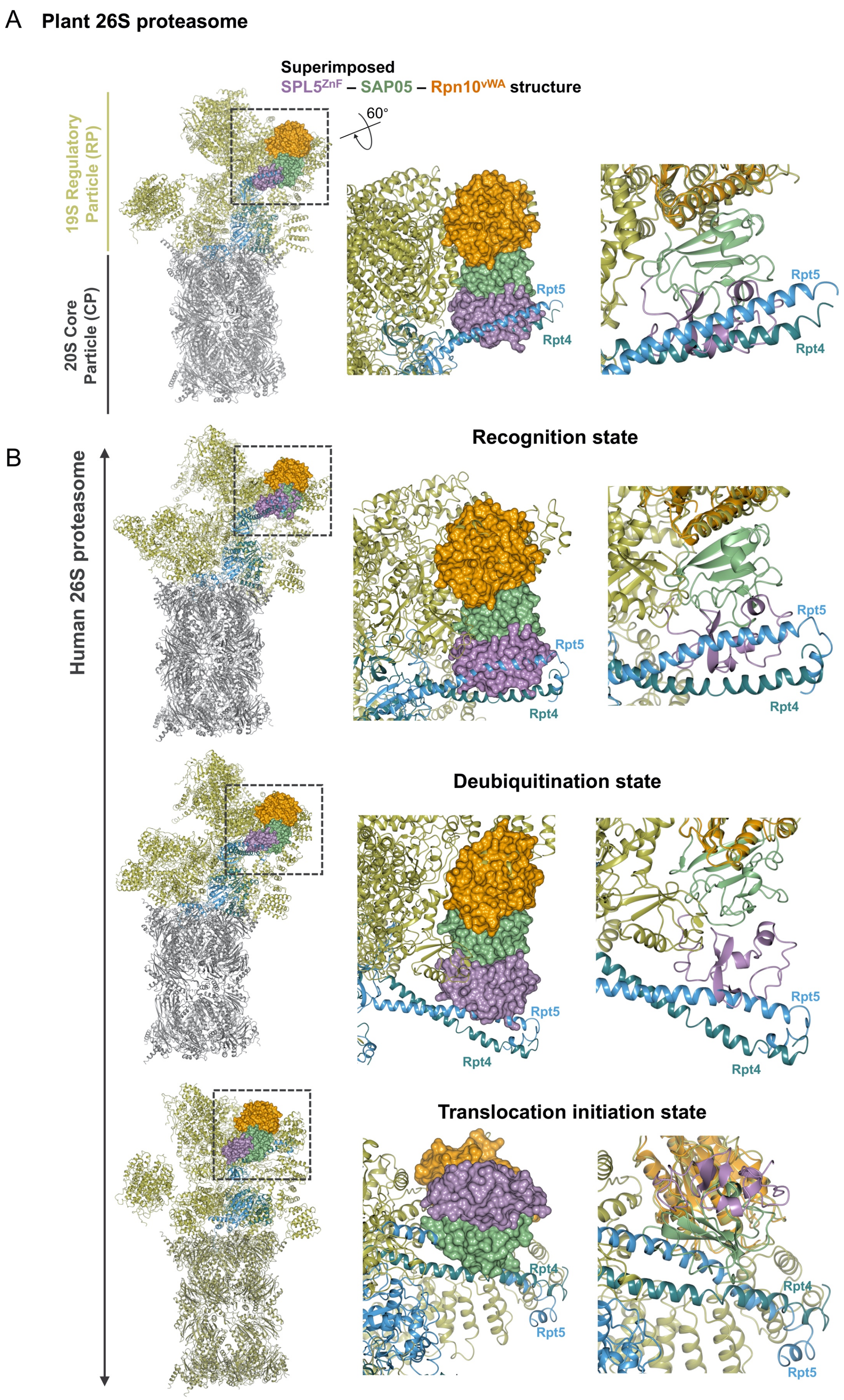
A
Plant 26S proteasome
Superimposed
SPL5ZnF – SAP05 – Rpn10vWA structure
60°
19S Regulatory Particle (RP)
20S Core Particle (CP)
Rpt5
Rpt4
Rpt5
Rpt4
B
Human 26S proteasome
Recognition state
Rpt5
Rpt4
Rpt5
Rpt4
Deubiquitination state
Rpt5
Rpt4
Rpt5
Rpt4
Translocation initiation state
Rpt4
Rpt5
Rpt4
Rpt5

**Fig. S16. Modelling of the Rpn10$^{vWA}$ – SAP05 – SPL5$^{ZnF}$ ternary structure with plant and human 26S proteasome components show potential clashes.** (A) Structural superimposition of the ternary structure on the spinach 26S proteasome (PDB 7QVE and PDB 8AMZ). Left, overview of the superimposition. Middle, enlarged surface view of the superimposed part within the dashed box. Right, enlarged cartoon view of the superimposed part. (B) Structural superimposition of the ternary structure on substrate recognition state (PDB 6MSD), deubiquitination state (PDB 6MSE) translocation initiation state (PDB 6MSH) of human 26S proteasome. Left, overview of the superimposition. Middle, enlarged surface view of the superimposed part within the dashed box. Right, enlarged cartoon view of the superimposed part. The superimposed structures highlight the clashes between SPL5$^{ZnF}$ and two $\alpha$-helices of 19S RP subunits, which are homologs of *A. thaliana* Rpt4 (turquoise) and Rpt5 (blue).

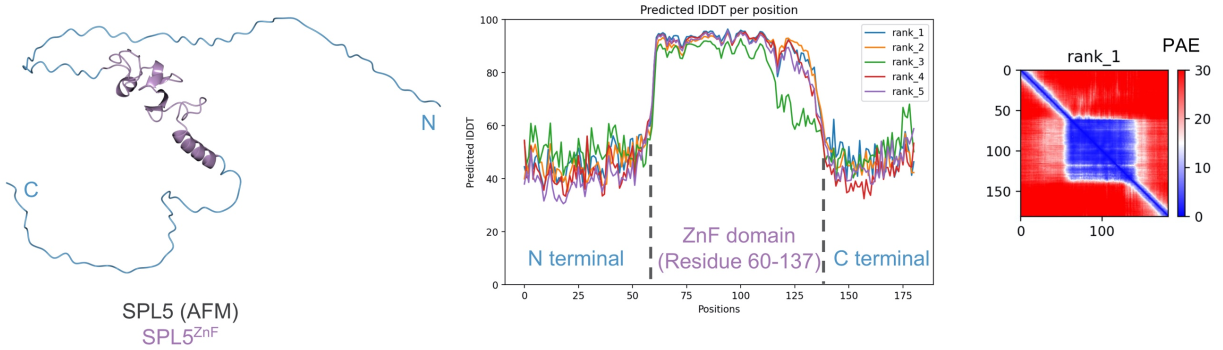


**Fig. S17. Structural prediction of full-length SPL5.** Left, AlphaFold model (AFM) for full-length SPL5 protein. N- and C- terminal parts are colored blue. $SPL5^{ZnF}$ domain is shown in purple. Middle, the predicted local Distance Difference Test (lDDT) value of SPL5 model, showing the quality of predicted models. Best model (rank_1) was shown in left panel. N- and C- terminal, and ZnF domain are labelled inside, with the same color as left. Right, predicted aligned error (PAE) of the rank_1 model. Blue means lower error scores, red means higher error scores.

## Table S1. Analysis of residues on SAP05 – SPL5$^{ZnF}$ interface from PDBsum

PDB code: 8pfc

Hydrogen bonds

| | Atom no. | Atom name | Res name | Res no. | Chain | | Atom no. | Atom name | Res name | Res no. | Chain | Distance |
|---|---|---|---|---|---|---|---|---|---|---|---|---|
| 1 | 262 | OD1 | ASP | 66 | A | <--> | 931 | NE2 | GLN | 77 | B | 3.10 |
| 2 | 350 | ND2 | ASN | 77 | A | <--> | 943 | OH | TYR | 78 | B | 3.16 |
| 3 | 350 | ND2 | ASN | 77 | A | <--> | 1154 | O | GLN | 105 | B | 2.84 |
| 4 | 371 | OE1 | GLU | 80 | A | <--> | 987 | NE2 | HIS | 82 | B | 3.11 |
| 5 | 559 | O | ARG | 104 | A | <--> | 1159 | NE2 | GLN | 105 | B | 2.64 |
| 6 | 578 | O | ASP | 106 | A | <--> | 1051 | NZ | LYS | 90 | B | 3.23 |
| 7 | 581 | OD1 | ASP | 106 | A | <--> | 1051 | NZ | LYS | 90 | B | 3.11 |
| 8 | 581 | OD1 | ASP | 106 | A | <--> | 1297 | NH1 | ARG | 121 | B | 3.06 |
| 9 | 582 | OD2 | ASP | 106 | A | <--> | 1298 | NH2 | ARG | 121 | B | 2.97 |

Non-bonded contacts

| | Atom no. | Atom name | Res name | Res no. | Chain | | Atom no. | Atom name | Res name | Res no. | Chain | Distance |
|---|---|---|---|---|---|---|---|---|---|---|---|---|
| 1 | 261 | CG | ASP | 66 | A | <--> | 931 | NE2 | GLN | 77 | B | 3.77 |
| 2 | 262 | OD1 | ASP | 66 | A | <--> | 931 | NE2 | GLN | 77 | B | 3.10 |
| 3 | 263 | OD2 | ASP | 66 | A | <--> | 923 | N | GLN | 77 | B | 3.85 |
| 4 | 281 | CG2 | THR | 68 | A | <--> | 931 | NE2 | GLN | 77 | B | 3.81 |
| 5 | 289 | CD2 | LEU | 69 | A | <--> | 921 | CE | LYS | 76 | B | 3.76 |
| 6 | 317 | NH1 | ARG | 73 | A | <--> | 921 | CE | LYS | 76 | B | 3.54 |
| 7 | 342 | O | GLY | 76 | A | <--> | 1159 | NE2 | GLN | 105 | B | 3.58 |
| 8 | 347 | CB | ASN | 77 | A | <--> | 1154 | O | GLN | 105 | B | 3.32 |
| 9 | 348 | CG | ASN | 77 | A | <--> | 1154 | O | GLN | 105 | B | 3.55 |
| 10 | 350 | ND2 | ASN | 77 | A | <--> | 943 | OH | TYR | 78 | B | 3.16 |
| 11 | 350 | ND2 | ASN | 77 | A | <--> | 1154 | O | GLN | 105 | B | 2.84 |
| 12 | 350 | ND2 | ASN | 77 | A | <--> | 1180 | OG | SER | 108 | B | 3.20 |
| 13 | 363 | CB | ALA | 79 | A | <--> | 940 | CE1 | TYR | 78 | B | 3.80 |
| 14 | 363 | CB | ALA | 79 | A | <--> | 987 | NE2 | HIS | 82 | B | 3.89 |
| 15 | 363 | CB | ALA | 79 | A | <--> | 1180 | OG | SER | 108 | B | 3.67 |
| 16 | 367 | O | GLU | 80 | A | <--> | 938 | CD1 | TYR | 78 | B | 3.41 |
| 17 | 367 | O | GLU | 80 | A | <--> | 986 | CE1 | HIS | 82 | B | 3.71 |
| 18 | 369 | CG | GLU | 80 | A | <--> | 976 | NH1 | ARG | 81 | B | 3.42 |
| 19 | 370 | CD | GLU | 80 | A | <--> | 976 | NH1 | ARG | 81 | B | 3.52 |
| 20 | 371 | OE1 | GLU | 80 | A | <--> | 986 | CE1 | HIS | 82 | B | 3.42 |
| 21 | 371 | OE1 | GLU | 80 | A | <--> | 987 | NE2 | HIS | 82 | B | 3.11 |
| 22 | 372 | OE2 | GLU | 80 | A | <--> | 976 | NH1 | ARG | 81 | B | 3.53 |
| 23 | 376 | O | PHE | 81 | A | <--> | 936 | CB | TYR | 78 | B | 3.51 |
| 24 | 377 | CB | PHE | 81 | A | <--> | 932 | N | TYR | 78 | B | 3.73 |
| 25 | 378 | CG | PHE | 81 | A | <--> | 927 | CB | GLN | 77 | B | 3.66 |
| 26 | 380 | CD2 | PHE | 81 | A | <--> | 927 | CB | GLN | 77 | B | 3.65 |
| 27 | 381 | CE1 | PHE | 81 | A | <--> | 929 | CD | GLN | 77 | B | 3.82 |
| 28 | 381 | CE1 | PHE | 81 | A | <--> | 930 | OE1 | GLN | 77 | B | 3.39 |
| 29 | 382 | CE2 | PHE | 81 | A | <--> | 977 | NH2 | ARG | 81 | B | 3.82 |
| 30 | 383 | CZ | PHE | 81 | A | <--> | 930 | OE1 | GLN | 77 | B | 3.40 |
| 31 | 401 | CB | ILE | 84 | A | <--> | 920 | CD | LYS | 76 | B | 3.87 |
| 32 | 404 | CD1 | ILE | 84 | A | <--> | 955 | OH | TYR | 79 | B | 3.81 |

| | | | | | | | | | | | | |
|---|---|---|---|---|---|---|---|---|---|---|---|---|
| 33 | 416 | CZ2 | TRP | 85 | A | <--> | 955 | OH | TYR | 79 | B | 3.45 |
| 34 | 417 | CZ3 | TRP | 85 | A | <--> | 1020 | OE2 | GLU | 86 | B | 3.81 |
| 35 | 418 | CH2 | TRP | 85 | A | <--> | 955 | OH | TYR | 79 | B | 3.35 |
| 36 | 418 | CH2 | TRP | 85 | A | <--> | 1020 | OE2 | GLU | 86 | B | 3.90 |
| 37 | 553 | CB | THR | 103 | A | <--> | 1157 | CD | GLN | 105 | B | 3.83 |
| 38 | 553 | CB | THR | 103 | A | <--> | 1158 | OE1 | GLN | 105 | B | 3.84 |
| 39 | 554 | OG1 | THR | 103 | A | <--> | 1304 | OG | SER | 122 | B | 3.89 |
| 40 | 555 | CG2 | THR | 103 | A | <--> | 1160 | N | GLN | 106 | B | 3.78 |
| 41 | 555 | CG2 | THR | 103 | A | <--> | 1161 | CA | GLN | 106 | B | 3.54 |
| 42 | 555 | CG2 | THR | 103 | A | <--> | 1164 | CB | GLN | 106 | B | 3.50 |
| 43 | 556 | N | ARG | 104 | A | <--> | 1159 | NE2 | GLN | 105 | B | 3.84 |
| 44 | 558 | C | ARG | 104 | A | <--> | 1159 | NE2 | GLN | 105 | B | 3.67 |
| 45 | 559 | O | ARG | 104 | A | <--> | 1157 | CD | GLN | 105 | B | 3.76 |
| 46 | 559 | O | ARG | 104 | A | <--> | 1159 | NE2 | GLN | 105 | B | 2.64 |
| 47 | 578 | O | ASP | 106 | A | <--> | 1050 | CE | LYS | 90 | B | 3.09 |
| 48 | 578 | O | ASP | 106 | A | <--> | 1051 | NZ | LYS | 90 | B | 3.23 |
| 49 | 580 | CG | ASP | 106 | A | <--> | 1050 | CE | LYS | 90 | B | 3.63 |
| 50 | 580 | CG | ASP | 106 | A | <--> | 1051 | NZ | LYS | 90 | B | 3.77 |
| 51 | 580 | CG | ASP | 106 | A | <--> | 1159 | NE2 | GLN | 105 | B | 3.71 |
| 52 | 580 | CG | ASP | 106 | A | <--> | 1297 | NH1 | ARG | 121 | B | 3.80 |
| 53 | 580 | CG | ASP | 106 | A | <--> | 1298 | NH2 | ARG | 121 | B | 3.66 |
| 54 | 581 | OD1 | ASP | 106 | A | <--> | 1050 | CE | LYS | 90 | B | 3.45 |
| 55 | 581 | OD1 | ASP | 106 | A | <--> | 1051 | NZ | LYS | 90 | B | 3.11 |
| 56 | 581 | OD1 | ASP | 106 | A | <--> | 1296 | CZ | ARG | 121 | B | 3.82 |
| 57 | 581 | OD1 | ASP | 106 | A | <--> | 1297 | NH1 | ARG | 121 | B | 3.06 |
| 58 | 581 | OD1 | ASP | 106 | A | <--> | 1298 | NH2 | ARG | 121 | B | 3.66 |
| 59 | 582 | OD2 | ASP | 106 | A | <--> | 1050 | CE | LYS | 90 | B | 3.61 |
| 60 | 582 | OD2 | ASP | 106 | A | <--> | 1159 | NE2 | GLN | 105 | B | 3.77 |
| 61 | 582 | OD2 | ASP | 106 | A | <--> | 1296 | CZ | ARG | 121 | B | 3.86 |
| 62 | 582 | OD2 | ASP | 106 | A | <--> | 1297 | NH1 | ARG | 121 | B | 3.84 |
| 63 | 582 | OD2 | ASP | 106 | A | <--> | 1298 | NH2 | ARG | 121 | B | 2.97 |

Salt bridges

| | Atom no. | Atom name | Res name | Res no. | Chain | | Atom no. | Atom name | Res name | Res no. | Chain | Distance |
|---|---|---|---|---|---|---|---|---|---|---|---|---|
| 1 | 372 | OE2 | GLU | 80 | A | <--> | 976 | NH1 | ARG | 81 | B | 3.53 |
| 2 | 371 | OE1 | GLU | 80 | A | <--> | 987 | NE2 | HIS | 82 | B | 3.11 |
| 3 | 581 | OD1 | ASP | 106 | A | <--> | 1051 | NZ | LYS | 90 | B | 3.11 |
| 4 | 582 | OD2 | ASP | 106 | A | <--> | 1298 | NH2 | ARG | 121 | B | 2.97 |

## Table S2. Analysis of residues on SAP05 – Rpn10$^{vWA}$ interface from PDBsum

| PDB code: 8pfd | | | | | | | | | | | | |
|---|---|---|---|---|---|---|---|---|---|---|---|---|
| Hydrogen bonds | | | | | | | | | | | | |
| | Atom no. | Atom name | Res name | Res no. | Chain | | Atom no. | Atom name | Res name | Res no. | Chain | Distance |
| 1 | 62 | NH1 | ARG | 43 | A | <--> | 1289 | OD2 | ASP | 68 | B | 3.10 |
| 2 | 110 | N | SER | 50 | A | <--> | 1031 | OE2 | GLU | 31 | B | 2.57 |
| 3 | 115 | OG | SER | 50 | A | <--> | 1031 | OE2 | GLU | 31 | B | 2.48 |
| 4 | 181 | NE2 | HIS | 58 | A | <--> | 1047 | O | ASN | 34 | B | 3.08 |
| 5 | 181 | NE2 | HIS | 58 | A | <--> | 1050 | OD1 | ASN | 34 | B | 2.86 |
| 6 | 731 | ND2 | ASN | 125 | A | <--> | 1288 | OD1 | ASP | 68 | B | 3.23 |
| Non-bonded contacts | | | | | | | | | | | | |
| | Atom no. | Atom name | Res name | Res no. | Chain | | Atom no. | Atom name | Res name | Res no. | Chain | Distance |
| 1 | 59 | CD | ARG | 43 | A | <--> | 1300 | C | GLY | 70 | B | 3.80 |
| 2 | 59 | CD | ARG | 43 | A | <--> | 1301 | O | GLY | 70 | B | 3.68 |
| 3 | 59 | CD | ARG | 43 | A | <--> | 1331 | CB | ALA | 74 | B | 3.75 |
| 4 | 61 | CZ | ARG | 43 | A | <--> | 1303 | CA | LYS | 71 | B | 3.84 |
| 5 | 62 | NH1 | ARG | 43 | A | <--> | 1289 | OD2 | ASP | 68 | B | 3.10 |
| 6 | 62 | NH1 | ARG | 43 | A | <--> | 1302 | N | LYS | 71 | B | 3.78 |
| 7 | 63 | NH2 | ARG | 43 | A | <--> | 1307 | CG | LYS | 71 | B | 3.89 |
| 8 | 77 | CG1 | VAL | 45 | A | <--> | 1301 | O | GLY | 70 | B | 3.67 |
| 9 | 77 | CG1 | VAL | 45 | A | <--> | 1327 | N | ALA | 74 | B | 3.78 |
| 10 | 78 | CG2 | VAL | 45 | A | <--> | 1301 | O | GLY | 70 | B | 3.85 |
| 11 | 82 | O | ASN | 46 | A | <--> | 1350 | CB | HIS | 77 | B | 3.87 |
| 12 | 82 | O | ASN | 46 | A | <--> | 1353 | CD2 | HIS | 77 | B | 3.58 |
| 13 | 83 | CB | ASN | 46 | A | <--> | 1351 | CG | HIS | 77 | B | 3.89 |
| 14 | 83 | CB | ASN | 46 | A | <--> | 1353 | CD2 | HIS | 77 | B | 3.36 |
| 15 | 83 | CB | ASN | 46 | A | <--> | 1355 | NE2 | HIS | 77 | B | 3.48 |
| 16 | 92 | CG1 | VAL | 47 | A | <--> | 1051 | ND2 | ASN | 34 | B | 3.25 |
| 17 | 97 | O | ASN | 48 | A | <--> | 1001 | NE2 | GLN | 27 | B | 3.54 |
| 18 | 101 | ND2 | ASN | 48 | A | <--> | 1353 | CD2 | HIS | 77 | B | 3.89 |
| 19 | 103 | CA | LEU | 49 | A | <--> | 1031 | OE2 | GLU | 31 | B | 3.55 |
| 20 | 104 | C | LEU | 49 | A | <--> | 1031 | OE2 | GLU | 31 | B | 3.49 |
| 21 | 110 | N | SER | 50 | A | <--> | 1001 | NE2 | GLN | 27 | B | 3.78 |
| 22 | 110 | N | SER | 50 | A | <--> | 1029 | CD | GLU | 31 | B | 3.69 |
| 23 | 110 | N | SER | 50 | A | <--> | 1031 | OE2 | GLU | 31 | B | 2.57 |
| 24 | 111 | CA | SER | 50 | A | <--> | 1031 | OE2 | GLU | 31 | B | 3.39 |
| 25 | 114 | CB | SER | 50 | A | <--> | 1029 | CD | GLU | 31 | B | 3.86 |
| 26 | 114 | CB | SER | 50 | A | <--> | 1030 | OE1 | GLU | 31 | B | 3.81 |
| 27 | 114 | CB | SER | 50 | A | <--> | 1031 | OE2 | GLU | 31 | B | 3.13 |
| 28 | 115 | OG | SER | 50 | A | <--> | 1029 | CD | GLU | 31 | B | 3.20 |
| 29 | 115 | OG | SER | 50 | A | <--> | 1030 | OE1 | GLU | 31 | B | 3.21 |
| 30 | 115 | OG | SER | 50 | A | <--> | 1031 | OE2 | GLU | 31 | B | 2.48 |
| 31 | 143 | O | ILE | 54 | A | <--> | 1059 | CD2 | LEU | 35 | B | 3.55 |
| 32 | 145 | CG1 | ILE | 54 | A | <--> | 1031 | OE2 | GLU | 31 | B | 3.78 |
| 33 | 165 | C | LYS | 57 | A | <--> | 1059 | CD2 | LEU | 35 | B | 3.65 |
| 34 | 166 | O | LYS | 57 | A | <--> | 1059 | CD2 | LEU | 35 | B | 3.70 |
| 35 | 172 | N | HIS | 58 | A | <--> | 1059 | CD2 | LEU | 35 | B | 3.50 |
| 36 | 173 | CA | HIS | 58 | A | <--> | 1059 | CD2 | LEU | 35 | B | 3.39 |
| 37 | 176 | CB | HIS | 58 | A | <--> | 1059 | CD2 | LEU | 35 | B | 3.55 |

| | | | | | | | | | | | | |
|---|---|---|---|---|---|---|---|---|---|---|---|---|
| 38 | 177 | CG | HIS | 58 | A | <--> | 1059 | CD2 | LEU | 35 | B | 3.85 |
| 39 | 179 | CD2 | HIS | 58 | A | <--> | 1047 | O | ASN | 34 | B | 3.72 |
| 40 | 179 | CD2 | HIS | 58 | A | <--> | 1050 | OD1 | ASN | 34 | B | 3.37 |
| 41 | 179 | CD2 | HIS | 58 | A | <--> | 1059 | CD2 | LEU | 35 | B | 3.75 |
| 42 | 180 | CE1 | HIS | 58 | A | <--> | 1075 | CA | GLY | 38 | B | 3.46 |
| 43 | 181 | NE2 | HIS | 58 | A | <--> | 1046 | C | ASN | 34 | B | 3.81 |
| 44 | 181 | NE2 | HIS | 58 | A | <--> | 1047 | O | ASN | 34 | B | 3.08 |
| 45 | 181 | NE2 | HIS | 58 | A | <--> | 1049 | CG | ASN | 34 | B | 3.85 |
| 46 | 181 | NE2 | HIS | 58 | A | <--> | 1050 | OD1 | ASN | 34 | B | 2.86 |
| 47 | 184 | C | GLU | 59 | A | <--> | 1104 | CG | GLN | 42 | B | 3.83 |
| 48 | 185 | O | GLU | 59 | A | <--> | 1104 | CG | GLN | 42 | B | 3.40 |
| 49 | 185 | O | GLU | 59 | A | <--> | 1105 | CD | GLN | 42 | B | 3.69 |
| 50 | 192 | CA | THR | 60 | A | <--> | 1106 | OE1 | GLN | 42 | B | 3.88 |
| 51 | 196 | OG1 | THR | 60 | A | <--> | 1075 | CA | GLY | 38 | B | 3.55 |
| 52 | 196 | OG1 | THR | 60 | A | <--> | 1077 | O | GLY | 38 | B | 3.76 |
| 53 | 196 | OG1 | THR | 60 | A | <--> | 1106 | OE1 | GLN | 42 | B | 3.67 |
| 54 | 196 | OG1 | THR | 60 | A | <--> | 1297 | CD2 | LEU | 69 | B | 3.53 |
| 55 | 224 | CD | LYS | 63 | A | <--> | 1107 | NE2 | GLN | 42 | B | 3.74 |
| 56 | 729 | CG | ASN | 125 | A | <--> | 1299 | CA | GLY | 70 | B | 3.80 |
| 57 | 730 | OD1 | ASN | 125 | A | <--> | 1299 | CA | GLY | 70 | B | 3.38 |
| 58 | 731 | ND2 | ASN | 125 | A | <--> | 1288 | OD1 | ASP | 68 | B | 3.23 |
| 59 | 751 | OH | TYR | 127 | A | <--> | 1292 | C | LEU | 69 | B | 3.82 |
| 60 | 751 | OH | TYR | 127 | A | <--> | 1294 | CB | LEU | 69 | B | 3.59 |
| 61 | 751 | OH | TYR | 127 | A | <--> | 1298 | N | GLY | 70 | B | 3.26 |
| 62 | 751 | OH | TYR | 127 | A | <--> | 1299 | CA | GLY | 70 | B | 3.61 |
| 63 | 800 | CD1 | TYR | 132 | A | <--> | 998 | CG | GLN | 27 | B | 3.71 |
| 64 | 800 | CD1 | TYR | 132 | A | <--> | 999 | CD | GLN | 27 | B | 3.56 |
| 65 | 800 | CD1 | TYR | 132 | A | <--> | 1000 | OE1 | GLN | 27 | B | 3.74 |
| 66 | 802 | CE1 | TYR | 132 | A | <--> | 997 | CB | GLN | 27 | B | 3.69 |
| 67 | 802 | CE1 | TYR | 132 | A | <--> | 998 | CG | GLN | 27 | B | 3.60 |
| 68 | 802 | CE1 | TYR | 132 | A | <--> | 999 | CD | GLN | 27 | B | 3.82 |
| 69 | 802 | CE1 | TYR | 132 | A | <--> | 1000 | OE1 | GLN | 27 | B | 3.87 |
| 70 | 804 | CZ | TYR | 132 | A | <--> | 997 | CB | GLN | 27 | B | 3.81 |
| 71 | 804 | CZ | TYR | 132 | A | <--> | 998 | CG | GLN | 27 | B | 3.85 |
| 72 | 805 | OH | TYR | 132 | A | <--> | 968 | N | SER | 24 | B | 3.58 |
| 73 | 805 | OH | TYR | 132 | A | <--> | 972 | CB | SER | 24 | B | 3.15 |
| Salt bridges | | | | | | | | | | | | |
| | Atom no. | Atom name | Res name | Res no. | Chain | | Atom no. | Atom name | Res name | Res no. | Chain | Distance |
| 1 | 62 | NH1 | ARG | 43 | A | <--> | 1289 | OD2 | ASP | 68 | B | 3.1 |

## References


1. N. S. Berrow *et al.*, A versatile ligation-independent cloning method suitable for high-throughput expression screening applications. *Nucleic Acids Res.* **35**, e45 (2007).
2. R. M. Benoit, R. N. Wilhelm, D. Scherer-Becker, C. Ostermeier, An improved method for fast, robust, and seamless integration of DNA fragments into multiple plasmids. *Protein Expr. Purif.* **45**, 66-71 (2006).
3. L. E. Bird *et al.*, Application of In-Fusion™ cloning for the parallel construction of E. coli expression vectors. *Methods Mol. Biol.* **1116**, 209-234 (2014).
4. H. Wickham, ggplot2. *Wiley Interdisciplinary Reviews: Computational Statistics* **3**, 180-185 (2011).
5. G. Winter *et al.*, DIALS: implementation and evaluation of a new integration package. *Acta Crystallogr. D Struct. Biol.* **74**, 85-97 (2018).
6. G. Winter, XIA2: an expert system for macromolecular crystallography data reduction. *J. Appl. Cryst.* **43**, 186-190 (2010).
7. P. R. Evans, G. N. Murshudov, How good are my data and what is the resolution? *Acta Crystallogr. D Biol. Crystallogr.* **69**, 1204-1214 (2013).
8. M. D. Winn *et al.*, Overview of the CCP4 suite and current developments. *Acta Crystallogr. D Biol. Crystallogr.* **67**, 235-242 (2011).
9. P. Skubák *et al.*, A new MR-SAD algorithm for the automatic building of protein models from low-resolution X-ray data and a poor starting model. *IUCrJ* **5**, 166-171 (2018).
10. P. Emsley, B. Lohkamp, W. G. Scott, K. Cowtan, Features and development of Coot. *Acta Crystallogr. D Biol. Crystallogr.* **66**, 486-501 (2010).
11. G. N. Murshudov *et al.*, REFMAC5 for the refinement of macromolecular crystal structures. *Acta Crystallogr. D Biol. Crystallogr.* **67**, 355-367 (2011).
12. A. J. McCoy *et al.*, Phaser crystallographic software. *J. Appl. Crystallogr.* **40**, 658-674 (2007).
13. A. Waterhouse *et al.*, SWISS-MODEL: homology modelling of protein structures and complexes. *Nucleic Acids Res.* **46**, W296-w303 (2018).

14. Y. Zhu *et al.*, Structural mechanism for nucleotide-driven remodeling of the AAA-ATPase unfoldase in the activated human 26S proteasome. *Nat. Commun.* **9**, 1360 (2018).
15. S. McNicholas, E. Potterton, K. S. Wilson, M. E. Noble, Presenting your structures: the CCP4mg molecular-graphics software. *Acta Crystallogr. D Biol. Crystallogr.* **67**, 386-394 (2011).
16. J. L. Hartley, G. F. Temple, M. A. Brasch, DNA cloning using in vitro site-specific recombination. *Genome Res.* **10**, 1788-1795 (2000).
17. X. Liang, L. Peng, C. H. Baek, F. Katzen, Single step BP/LR combined Gateway reactions. *Biotechniques* **55**, 265-268 (2013).
18. W. Huang *et al.*, Parasitic modulation of host development by ubiquitin-independent protein degradation. *Cell* **184**, 5201-5214.e12 (2021).
19. S. D. Yoo, Y. H. Cho, J. Sheen, Arabidopsis mesophyll protoplasts: a versatile cell system for transient gene expression analysis. *Nat. Protoc.* **2**, 1565-1572 (2007).

# Uncropped Gels and Western Blots

## Fig. 1F

Coomassie stained gels showing the proteins from different gel filtration fractions. Red dashed rectangles indicate the cropped image shown in the main and supplementary figures.

### GF of SPL5$^{ZnF}$-SAP05-Rpn10$^{vWA}$ ternary complex

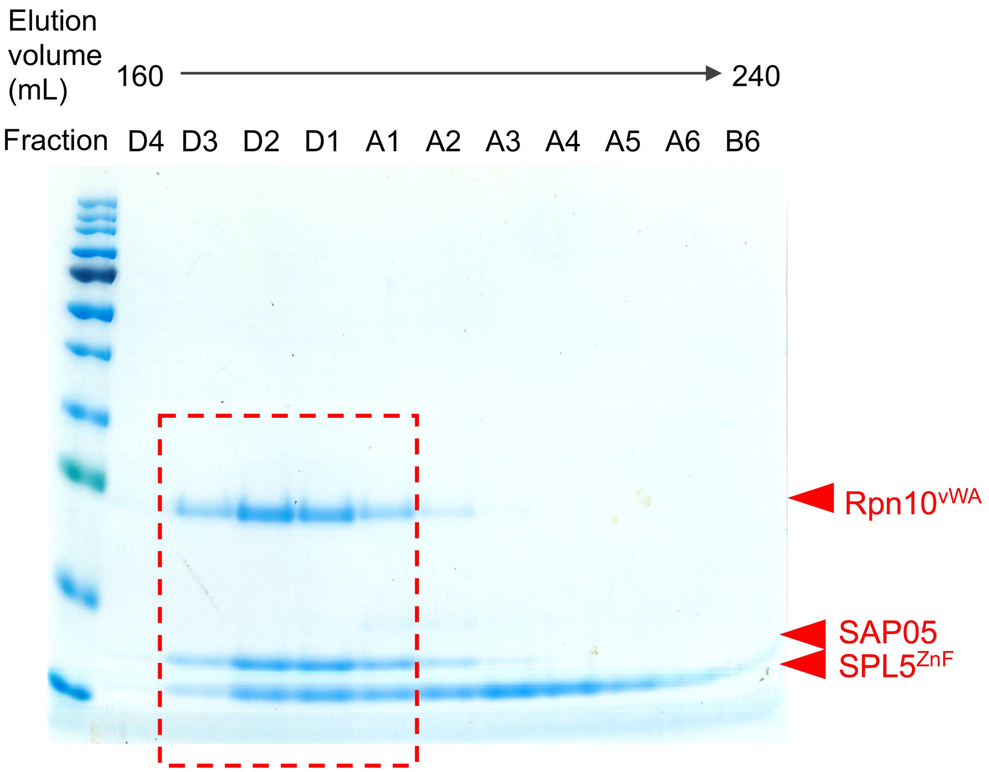

## *SI Appendix* Fig. S1B

### GF of SAP05-Rpn10$^{vWA}$ complex

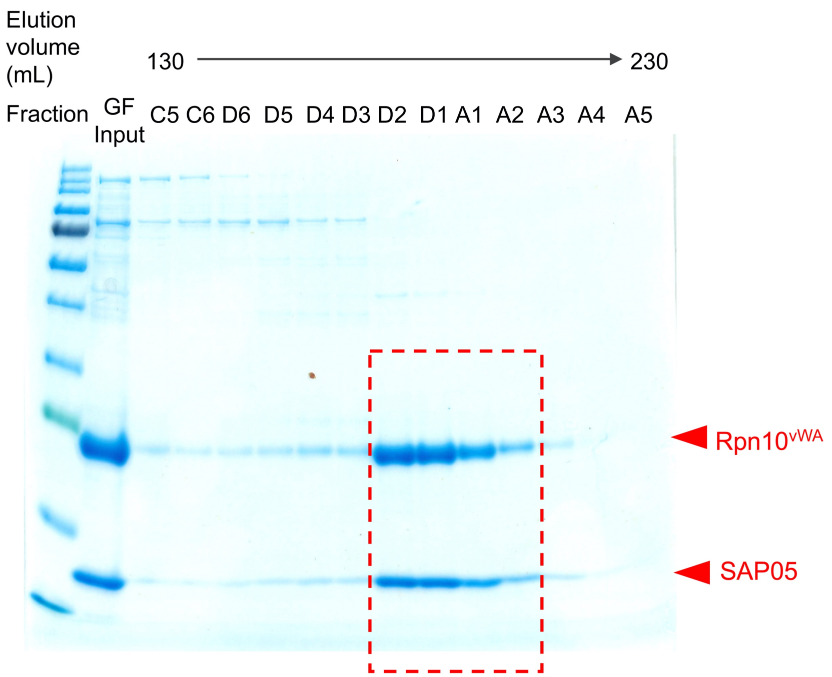


### GF of SAP05-SPL5$^{ZnF}$ complex

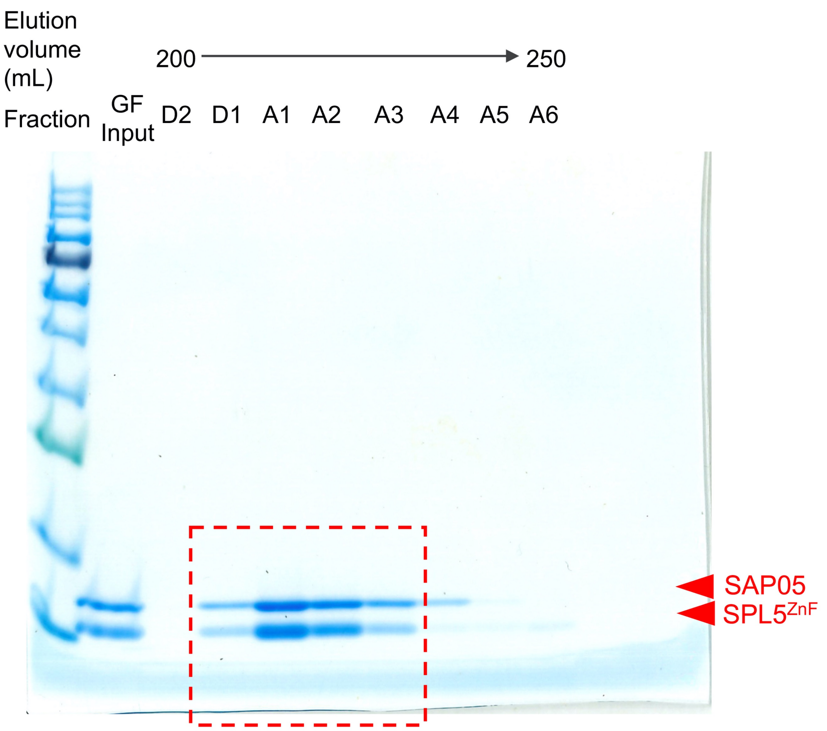

## GF of SAP05

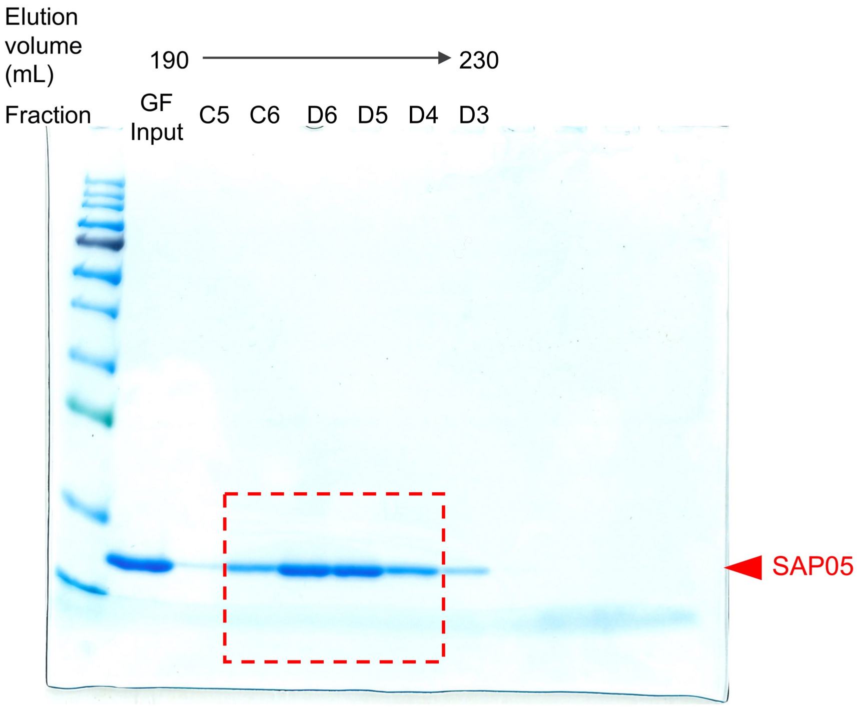


## GF of SPL5$^{ZnF}$

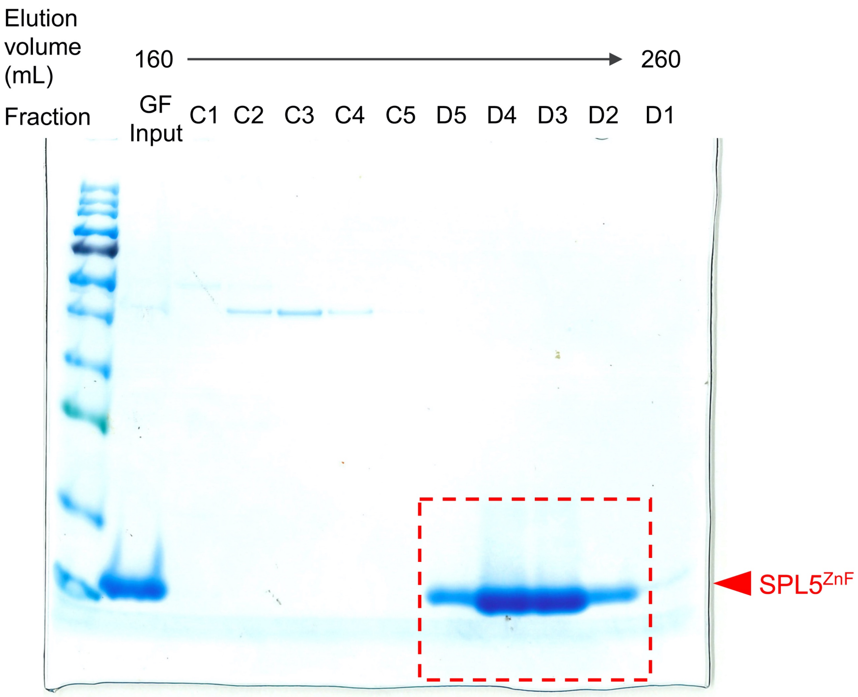

# Fig. 2D

The degradation efficiency (Rel HA/rbcL) was calculated as the HA intensity divided by the RuBisCo large subunit (rbcL) intensity from the same sample, normalized to the intensity of the sample with the highest ratio from the same leaf. The expected size for SAP05, SPL5, and GATA19 is indicated. Experiments included in the main figures are indicated with red dashed rectangles. *Failed experiments due to errors in controls.

| | SAP05 D66A | | | | | | | | | | | |
|---|---|---|---|---|---|---|---|---|---|---|---|---|
| Leaf | Repeat 1* | | | | Repeat 2 | | | | Repeat 3 | | | |
| HA-SPL5 | + | + | + | - | + | + | + | - | + | + | + | - |
| SAP05 (WT) | + | - | - | - | + | - | - | - | + | - | - | - |
| SAP05 (mut) | - | + | - | + | - | + | - | + | - | + | - | + |
| Lane | A | B | C | D | E | F | G | H | I | J | K | L |
| Rel HA/rbcL | 0.24 | 1.00 | 0.03 | 0.00 | 0.10 | 0.88 | 1.00 | 0.00 | 0.12 | 0.74 | 1.00 | 0.00 |

| | SAP05 D66E | | | | | | | | | | | |
|---|---|---|---|---|---|---|---|---|---|---|---|---|
| Leaf | Repeat 1 | | | | Repeat 2* | | | | Repeat 3 | | | |
| HA-SPL5 | + | + | + | - | + | + | + | - | + | + | + | - |
| SAP05 (WT) | + | - | - | - | + | - | - | - | + | - | - | - |
| SAP05 (mut) | - | + | - | + | - | + | - | + | - | + | - | + |
| Lane | 1 | 2 | 3 | 4 | 5 | 6 | 7 | 8 | 9 | 10 | 11 | 12 |
| Rel HA/rbcL | 0.08 | 0.09 | 1.00 | 0.00 | 0.88 | 1.00 | 0.37 | 0.00 | 0.20 | 0.19 | 1.00 | 0.00 |

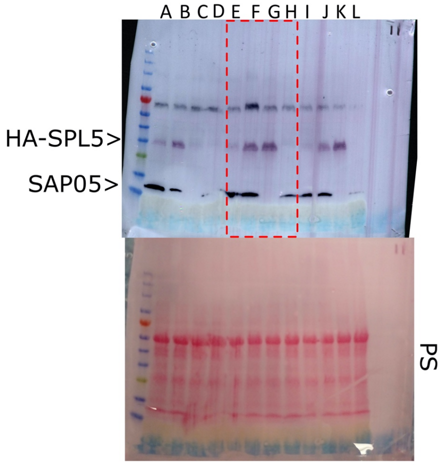


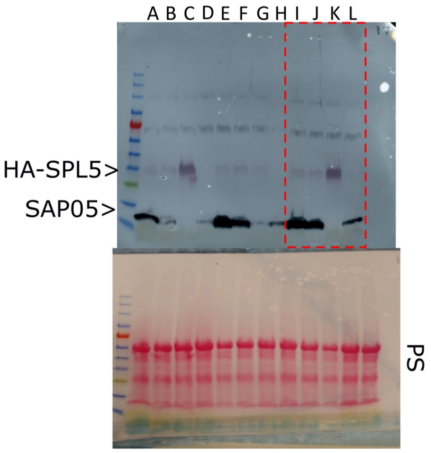

| Leaf | SAP05 G76W | | | | | | | | | | | |
|---|---|---|---|---|---|---|---|---|---|---|---|---|
| | Repeat 1 | | | | Repeat 2 | | | | Repeat 3 | | | |
| HA-SPL5 | + | + | + | - | + | + | + | - | + | + | + | - |
| SAP05 (WT) | + | - | - | - | + | - | - | - | + | - | - | - |
| SAP05 (mut) | - | + | - | + | - | + | - | + | - | + | - | + |
| Lane | A | B | C | D | E | F | G | H | I | J | K | L |
| Rel HA/rbcL | 0.04 | 0.63 | 1.00 | 0.01 | 0.10 | 0.60 | 1.00 | 0.00 | 0.09 | 0.77 | 1.00 | 0.00 |

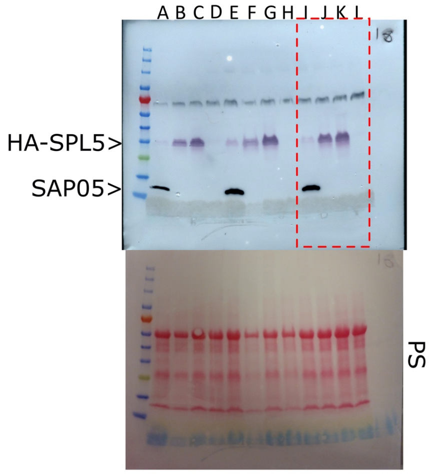


| Leaf | SAP05 N77D | | | | | | | | | | | |
|---|---|---|---|---|---|---|---|---|---|---|---|---|
| | Repeat 1 | | | | Repeat 2 | | | | Repeat 3 | | | |
| HA-SPL5 | + | + | + | - | + | + | + | - | + | + | + | - |
| SAP05 (WT) | + | - | - | - | + | - | - | - | + | - | - | - |
| SAP05 (mut) | - | + | - | + | - | + | - | + | - | + | - | + |
| Lane | A | B | C | D | E | F | G | H | I | J | K | L |
| Rel HA/rbcL | 0.11 | 0.21 | 1.00 | 0.01 | 0.19 | 0.64 | 1.00 | 0.01 | 0.15 | 0.57 | 1.00 | 0.03 |

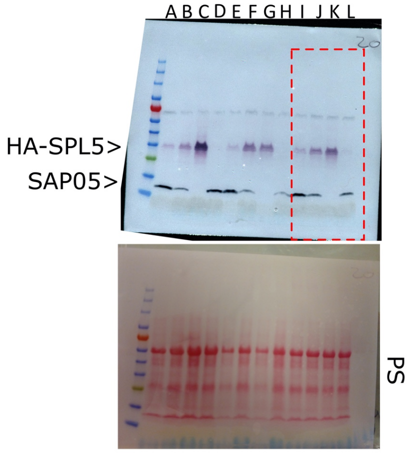


| Leaf | SAP05 N77A | | | | | | | | | | | |
|---|---|---|---|---|---|---|---|---|---|---|---|---|
| | Repeat 1 | | | | Repeat 2* | | | | Repeat 3 | | | |
| HA-SPL5 | + | + | + | - | + | + | + | - | + | + | + | - |
| SAP05 (WT) | + | - | - | - | + | - | - | - | + | - | - | - |
| SAP05 (mut) | - | + | - | + | - | + | - | + | - | + | - | + |
| Lane | A | B | C | D | E | F | G | H | I | J | K | L |
| Rel HA/rbcL | 0.06 | 0.09 | 1.00 | 0.02 | 1.00 | 0.82 | 0.62 | 0.00 | 0.19 | 0.08 | 1.00 | 0.00 |

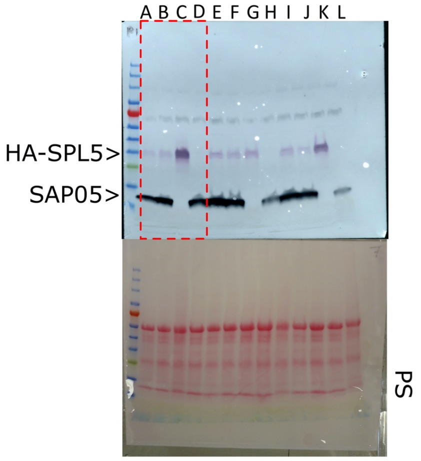


| Leaf | SAP05 N77R | | | | | | | | | | | |
|---|---|---|---|---|---|---|---|---|---|---|---|---|
| | Repeat 1 | | | | Repeat 2 | | | | Repeat 3 | | | |
| HA-SPL5 | + | + | + | - | + | + | + | - | + | + | + | - |
| SAP05 (WT) | + | - | - | - | + | - | - | - | + | - | - | - |
| SAP05 (mut) | - | + | - | + | - | + | - | + | - | + | - | + |
| Lane | A | B | C | D | E | F | G | H | I | J | K | L |
| Rel HA/rbcL | 0.19 | 1.00 | 0.59 | 0.02 | 0.14 | 1.00 | 0.70 | 0.00 | 0.10 | 1.00 | 0.87 | 0.01 |

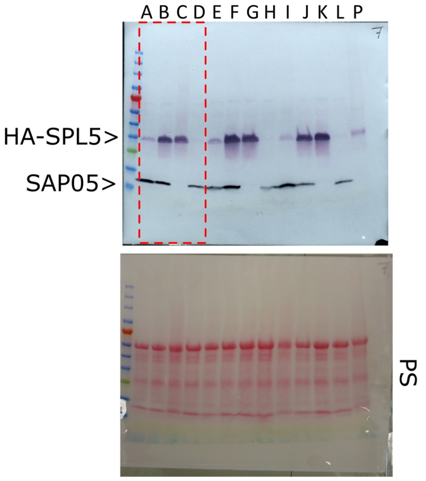

| | SAP05 E80A | | | | | | | | | | | |
|---|---|---|---|---|---|---|---|---|---|---|---|---|
| Leaf | Repeat 1 | | | | Repeat 2 | | | | Repeat 3 | | | |
| HA-SPL5 | + | + | + | - | + | + | + | - | + | + | + | - |
| SAP05 (WT) | + | - | - | - | + | - | - | - | + | - | - | - |
| SAP05 (mut) | - | + | - | + | - | + | - | + | - | + | - | + |
| Lane | A | B | C | D | E | F | G | H | I | J | K | L |
| Rel HA/rbcL | 0.12 | 0.18 | 1.00 | 0.01 | 0.08 | 0.06 | 1.00 | 0.02 | 0.30 | 0.18 | 1.00 | 0.02 |

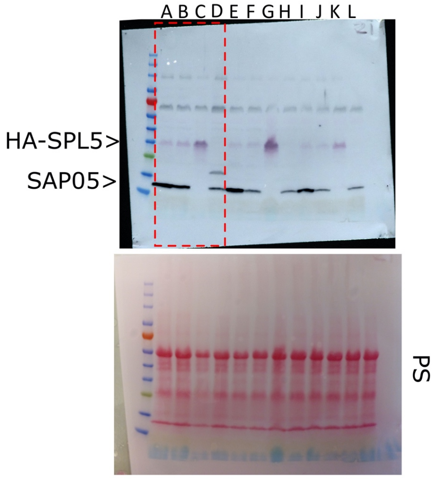


| | SAP05 E80R | | | | | | | | | | | |
|---|---|---|---|---|---|---|---|---|---|---|---|---|
| Leaf | Repeat 1 | | | | Repeat 2 | | | | Repeat 3 | | | |
| HA-SPL5 | + | + | + | - | + | + | + | - | + | + | + | - |
| SAP05 (WT) | + | - | - | - | + | - | - | - | + | - | - | - |
| SAP05 (mut) | - | + | - | + | - | + | - | + | - | + | - | + |
| Lane | A | B | C | D | E | F | G | H | I | J | K | L |
| Rel HA/rbcL | 0.08 | 0.09 | 1.00 | 0.00 | 0.22 | 0.17 | 1.00 | 0.11 | 0.12 | 0.12 | 1.00 | 0.01 |

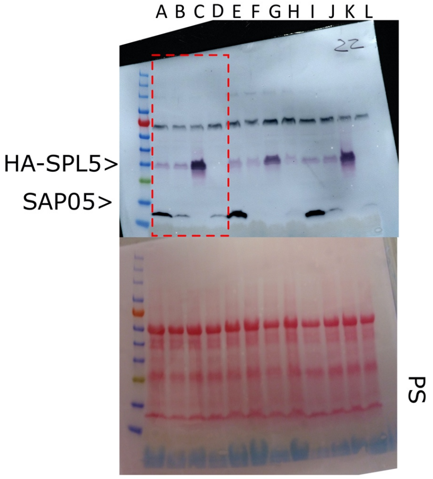


| | SAP05 D106A | | | | | | | | | | | |
|---|---|---|---|---|---|---|---|---|---|---|---|---|
| Leaf | Repeat 1 | | | | Repeat 2* | | | | Repeat 3 | | | |
| HA-SPL5 | + | + | + | - | + | + | + | - | + | + | + | - |
| SAP05 (WT) | + | - | - | - | + | - | - | - | + | - | - | - |
| SAP05 (mut) | - | + | - | + | - | + | - | + | - | + | - | + |
| Lane | A | B | C | D | E | F | G | H | I | J | K | L |
| Rel HA/rbcL | 0.10 | 1.00 | 0.85 | 0.00 | 0.43 | 1.00 | 0.61 | 0.04 | 0.11 | 1.00 | 01.02 | 0.01 |

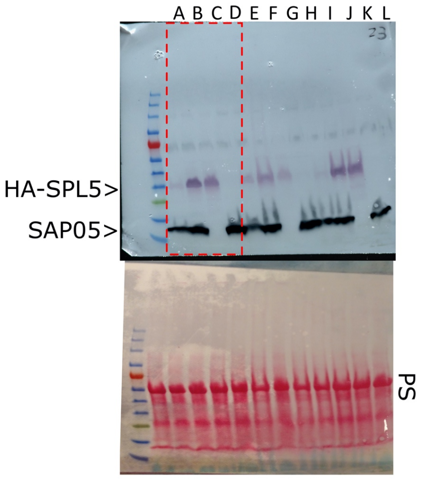


| | SAP05 D106R | | | | | | | | | | | |
|---|---|---|---|---|---|---|---|---|---|---|---|---|
| Leaf | Repeat 1 | | | | Repeat 2* | | | | Repeat 3 | | | |
| HA-SPL5 | + | + | + | - | + | + | + | - | + | + | + | - |
| SAP05 (WT) | + | - | - | - | + | - | - | - | + | - | - | - |
| SAP05 (mut) | - | + | - | + | - | + | - | + | - | + | - | + |
| Lane | A | B | C | D | E | F | G | H | I | J | K | L |
| Rel HA/rbcL | 0.03 | 1.00 | 0.51 | 0.01 | 0.10 | 1.00 | 0.07 | 0.05 | 0.04 | 1.00 | 0.61 | 0.07 |

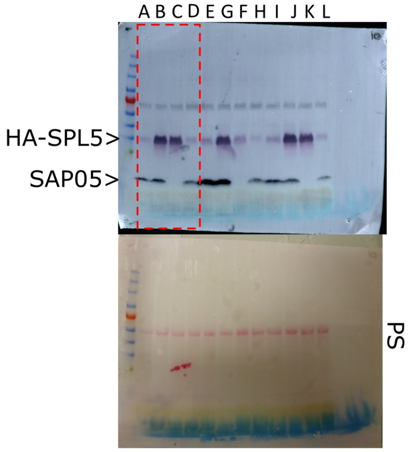

# Fig. 2F

| Leaf | SAP05 D66A | | | | | | | | | | | |
|---|---|---|---|---|---|---|---|---|---|---|---|---|
| | Repeat 1 | | | | Repeat 2 | | | | Repeat 3 | | | |
| HA-Gata19 | + | + | + | - | + | + | + | - | + | + | + | - |
| SAP05 (WT) | + | - | - | - | + | - | - | - | + | - | - | - |
| SAP05 (mut) | - | + | - | + | - | + | - | + | - | + | - | + |
| Lane | A | B | C | D | E | F | G | H | I | J | K | L |
| Rel HA/rbcL | 0.04 | 0.89 | 1.00 | 0.01 | 0.01 | 0.47 | 1.00 | 0.00 | 0.07 | 0.90 | 1.00 | 0.00 |

| Leaf | SAP05 N77R | | | | | | | | | | | |
|---|---|---|---|---|---|---|---|---|---|---|---|---|
| | Repeat 1 | | | | Repeat 2 | | | | Repeat 3 | | | |
| HA-Gata19 | + | + | + | - | + | + | + | - | + | + | + | - |
| SAP05 (WT) | + | - | - | - | + | - | - | - | + | - | - | - |
| SAP05 (mut) | - | + | - | + | - | + | - | + | - | + | - | + |
| Lane | A | B | C | D | E | F | G | H | I | J | K | L |
| Rel HA/rbcL | 0.05 | 0.02 | 1.00 | 0.01 | 0.12 | 0.03 | 1.00 | 0.04 | 0.01 | 0.00 | 1.00 | 0.01 |

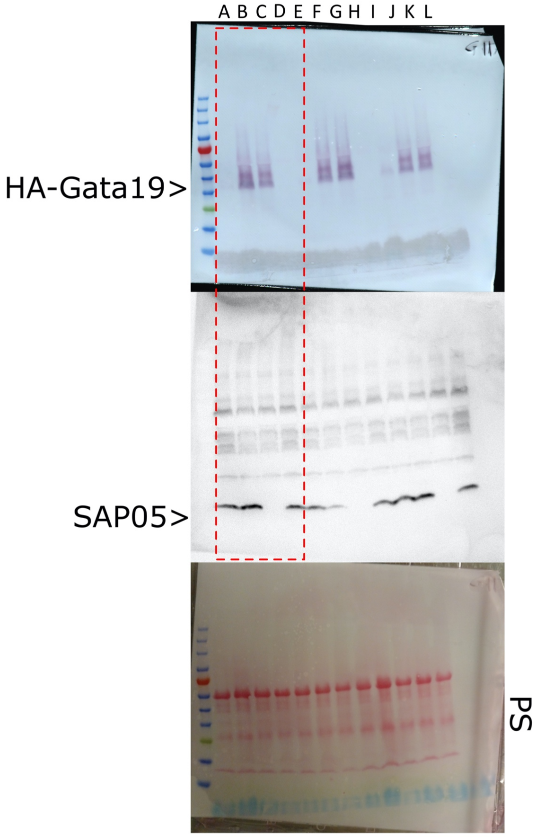


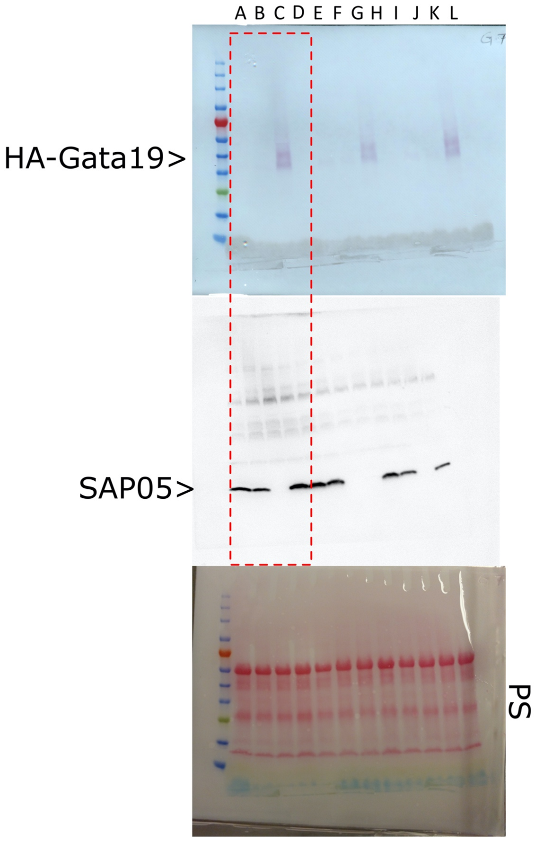

# Fig. 3B

The degradation efficiency (Rel HA/rbcL) was calculated as the HA intensity divided by the RuBisCo large subunit (rbcL) intensity from the same sample, then normalized to the coexpression of GFP and HA-SPL5 sample. Red dashed rectangles indicate the cropped image shown in the main figure.

Repeat 1

| | | | | | SAP05 mutant | | | | | | | | | |
|---|---|---|---|---|---|---|---|---|---|---|---|---|---|---|
| | | | GFP | SAP05 | S50A | H58W | S50A-H58W | H58A | T60W | H58A-T60W | Y127W | S50A-Y127W | N125A | S50A-Y127W |
| HA-SPL5 | – | + | + | + | + | + | + | + | + | + | + | + | + | + |
| Lane | A | B | C | D | E | F | G | H | I | J | K | L | M | N |
| Rel HA/rbcL | 0.12 | 0.98 | 1.00 | 0.16 | 0.14 | 0.13 | 0.22 | 0.12 | 0.16 | 0.27 | 0.14 | 0.14 | 0.12 | 0.13 |

Repeat 2

| | | | | | SAP05 mutant | | | | | | | | | |
|---|---|---|---|---|---|---|---|---|---|---|---|---|---|---|
| | | GFP | GFP | SAP05 | S50A | H58W | S50A-H58W | H58A | T60W | H58A-T60W | Y127W | S50A-Y127W | N125A | S50A-Y127W |
| HA-SPL5 | – | – | + | + | + | + | + | + | + | + | + | + | + | + |
| Lane | A | B | C | D | E | F | G | H | I | J | K | L | M | N |
| Rel HA/rbcL | 0.02 | 0.02 | 1.00 | 0.02 | 0.02 | 0.01 | 0.57 | 0.01 | 0.03 | 0.80 | 0.04 | 0.02 | 0.02 | 0.04 |

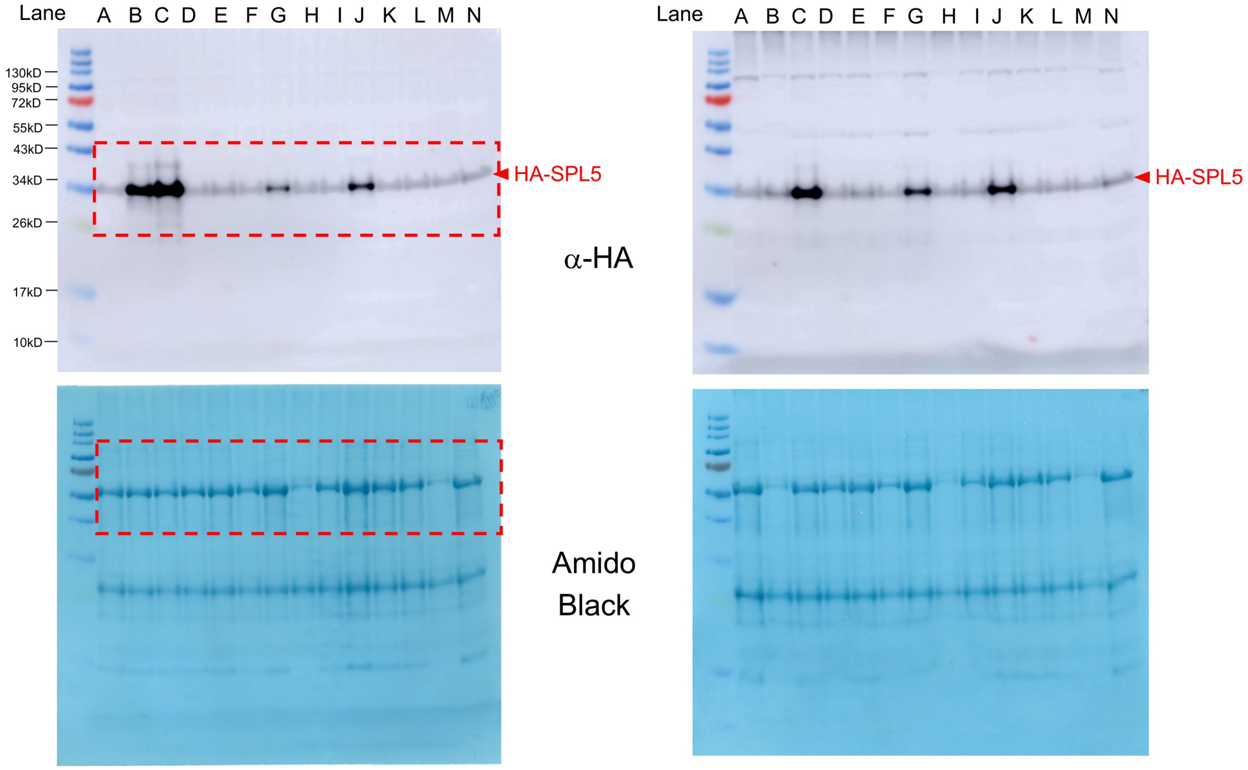

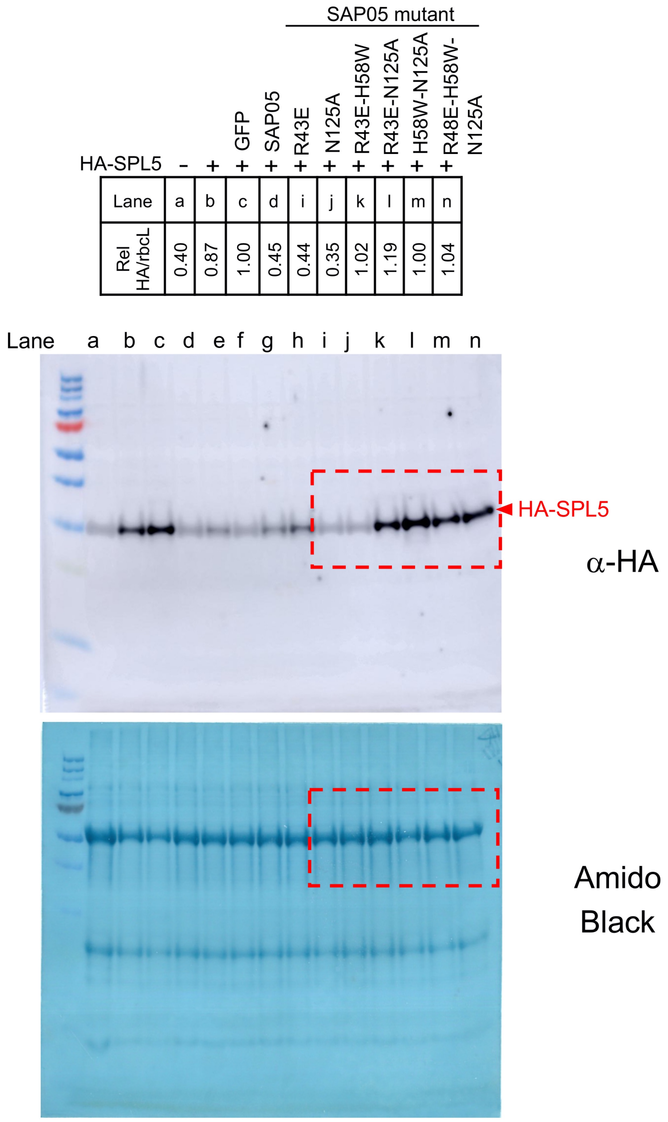
SAP05 mutant
HA-SPL5
– + + GFP + SAP05 + R43E + N125A + R43E-H58W + R43E-N125A + H58W-N125A + R48E-H58W-N125A
Lane a b c d i j k l m n
Rel HA/rbcL 0.40 0.87 1.00 0.45 0.44 0.35 1.02 1.19 1.00 1.04
Lane a b c d e f g h i j k l m n
HA-SPL5
α-HA
Amido Black

# *SI Appendix* Fig. S10

The degradation efficiency (Rel HA/rbcL) was calculated as the HA intensity divided by the RuBisCo large subunit (rbcL) intensity from the same sample, normalized to the intensity of the sample with the highest ratio from the same leaf. The expected size for SAP05, SPL5, and GATA19 is indicated. Experiments included in the main figures are indicated with red dashed rectangles. *Failed experiments due to errors in controls.

| | SAP05 R43A | | | | | | | | | | | |
|---|---|---|---|---|---|---|---|---|---|---|---|---|
| Leaf | Repeat 1 | | | | Repeat 2 | | | | Repeat 3 | | | |
| HA-SPL5 | + | + | + | - | + | + | + | - | + | + | + | - |
| SAP05 (WT) | + | - | - | - | + | - | - | - | + | - | - | - |
| SAP05 (mut) | - | + | - | + | - | + | - | + | - | + | - | + |
| Lane | A | B | C | D | E | F | G | H | I | J | K | L |
| Rel HA/rbcL | 0.38 | 0.28 | 1.00 | 0.03 | 0.47 | 0.46 | 1.00 | 0.03 | 0.14 | 0.19 | 1.00 | 0.02 |

| | SAP05 R43E | | | | | | | | | | | |
|---|---|---|---|---|---|---|---|---|---|---|---|---|
| Leaf | Repeat 1 | | | | Repeat 2 | | | | Repeat 3 | | | |
| HA-SPL5 | + | + | + | - | + | + | + | - | + | + | + | - |
| SAP05 (WT) | + | - | - | - | + | - | - | - | + | - | - | - |
| SAP05 (mut) | - | + | - | + | - | + | - | + | - | + | - | + |
| Lane | A | B | C | D | E | F | G | H | I | J | K | L |
| Rel HA/rbcL | 0.07 | 0.21 | 1.00 | 0.00 | 0.13 | 0.41 | 1.00 | 0.01 | 0.09 | 0.32 | 1.00 | 0.01 |

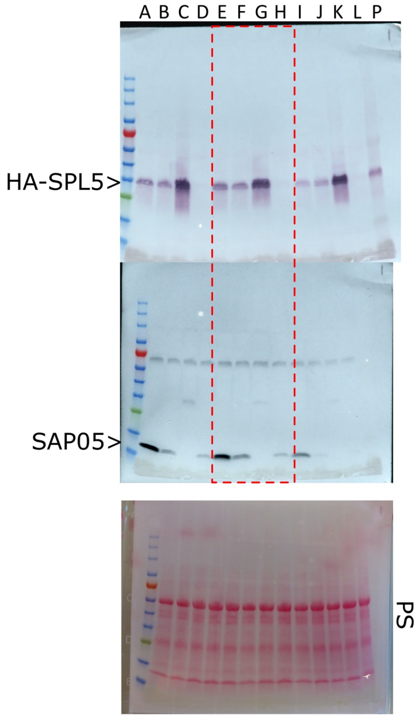


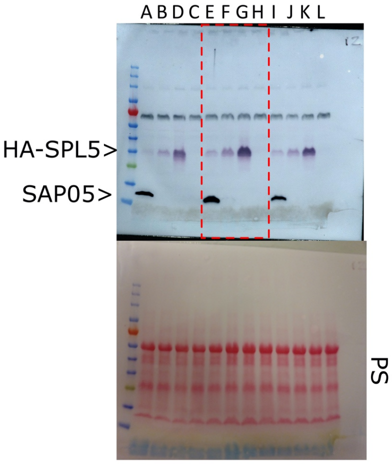

| | SAP05 S50A | | | | | | | | | | | |
|---|---|---|---|---|---|---|---|---|---|---|---|---|
| Leaf | Repeat 1 | | | | Repeat 2* | | | | Repeat 3 | | | |
| HA-SPL5 | + | + | + | - | + | + | + | - | + | + | + | - |
| SAP05 (WT) | + | - | - | - | + | - | - | - | + | - | - | - |
| SAP05 (mut) | - | + | - | + | - | + | - | + | - | + | - | + |
| Lane | A | B | C | D | E | F | G | H | I | J | K | L |
| Rel HA/rbcL | 0.16 | 0.33 | 1.00 | 0.00 | 1.00 | 0.59 | 0.99 | 0.00 | 0.43 | 0.26 | 1.00 | 0.00 |

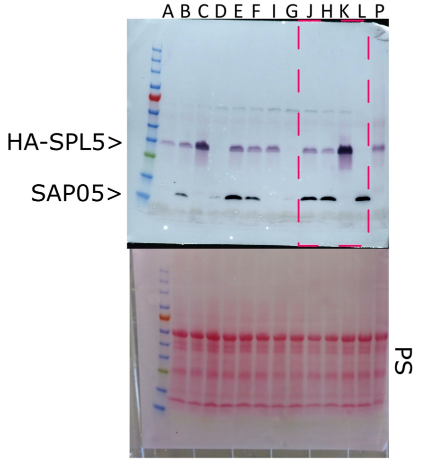


| | SAP05 H58A | | | | | | | | | | | |
|---|---|---|---|---|---|---|---|---|---|---|---|---|
| Leaf | Repeat 1 | | | | Repeat 2 | | | | Repeat 3 | | | |
| HA-SPL5 | + | + | + | - | + | + | + | - | + | + | + | - |
| SAP05 (WT) | + | - | - | - | + | - | - | - | + | - | - | - |
| SAP05 (mut) | - | + | - | + | - | + | - | + | - | + | - | + |
| Lane | A | B | C | D | E | F | G | H | I | J | K | L |
| Rel HA/rbcL | 0.55 | 0.23 | 1.00 | 0.01 | 0.26 | 0.21 | 1.00 | 0.00 | 0.27 | 0.19 | 1.00 | 0.00 |

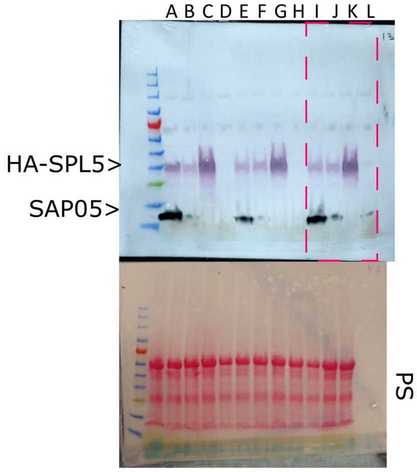


| | SAP05 H58W | | | | | | | | | | | |
|---|---|---|---|---|---|---|---|---|---|---|---|---|
| Leaf | Repeat 1 | | | | Repeat 2* | | | | Repeat 3 | | | |
| HA-SPL5 | + | + | + | - | + | + | + | - | + | + | + | - |
| SAP05 (WT) | + | - | - | - | + | - | - | - | + | - | - | - |
| SAP05 (mut) | - | + | - | + | - | + | - | + | - | + | - | + |
| Lane | A | B | C | D | E | F | G | H | I | J | K | L |
| Rel HA/rbcL | 0.14 | 0.03 | 1.00 | 0.01 | 1.00 | 0.14 | 0.40 | 0.02 | 0.15 | 0.01 | 1.00 | 0.00 |

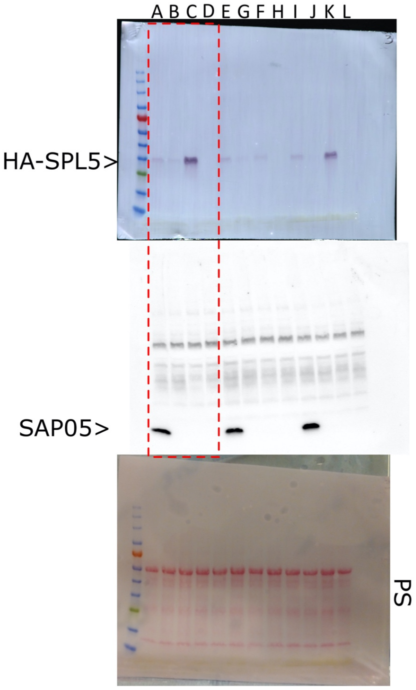


| | SAP05 T60W | | | | | | | | | | | |
|---|---|---|---|---|---|---|---|---|---|---|---|---|
| Leaf | Repeat 1 | | | | Repeat 2 | | | | Repeat 3* | | | |
| HA-SPL5 | + | + | + | - | + | + | + | - | + | + | + | - |
| SAP05 (WT) | + | - | - | - | + | - | - | - | + | - | - | - |
| SAP05 (mut) | - | + | - | + | - | + | - | + | - | + | - | + |
| Lane | A | B | C | D | E | F | G | H | I | J | K | L |
| Rel HA/rbcL | 0.31 | 0.31 | 1.00 | 0.01 | 0.72 | 0.31 | 1.00 | 0.02 | 1.00 | 0.43 | 0.18 | 0.02 |

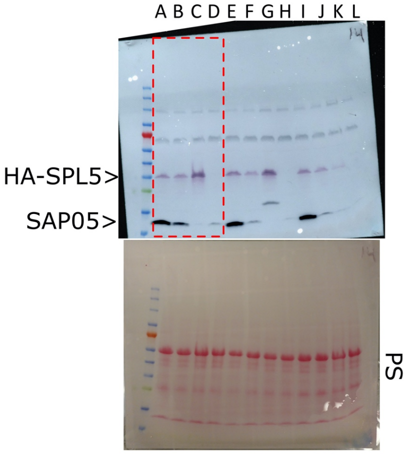

| Leaf | SAP05 N125A | | | | | | | | | | | |
|---|---|---|---|---|---|---|---|---|---|---|---|---|
| | Repeat 1 | | | | Repeat 2* | | | | Repeat 3 | | | |
| HA-SPL5 | + | + | + | - | + | + | + | - | + | + | + | - |
| SAP05 (WT) | + | - | - | - | + | - | - | - | + | - | - | - |
| SAP05 (mut) | - | + | - | + | - | + | - | + | - | + | - | + |
| Lane | A | B | C | D | E | F | G | H | I | J | K | L |
| Rel HA/rbcL | 0.11 | 0.05 | 1.00 | 0.03 | 1.00 | 0.17 | 0.58 | 0.01 | 0.25 | 0.12 | 1.00 | 0.01 |

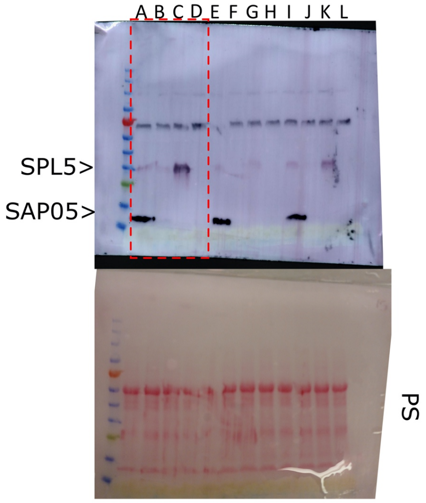


| Leaf | SAP05 N125D | | | | | | | | | | | |
|---|---|---|---|---|---|---|---|---|---|---|---|---|
| | Repeat 1 | | | | Repeat 2 | | | | Repeat 3* | | | |
| HA-SPL5 | + | + | + | - | + | + | + | - | + | + | + | - |
| SAP05 (WT) | + | - | - | - | + | - | - | - | + | - | - | - |
| SAP05 (mut) | - | + | - | + | - | + | - | + | - | + | - | + |
| Lane | A | B | C | D | E | F | G | H | I | J | K | L |
| Rel HA/rbcL | 0.37 | 0.07 | 1.00 | 0.01 | 0.16 | 0.03 | 1.00 | 0.00 | 1.00 | 0.20 | 0.58 | 0.03 |

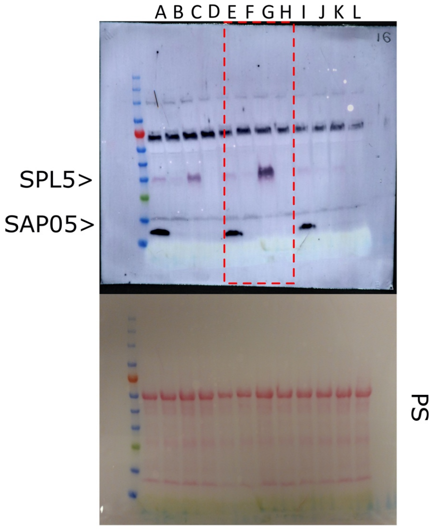


| Leaf | SAP05 Y127W | | | | | | | | | | | |
|---|---|---|---|---|---|---|---|---|---|---|---|---|
| | Repeat 1 | | | | Repeat 2 | | | | Repeat 3 | | | |
| HA-SPL5 | + | + | + | - | + | + | + | - | + | + | + | - |
| SAP05 (WT) | + | - | - | - | + | - | - | - | + | - | - | - |
| SAP05 (mut) | - | + | - | + | - | + | - | + | - | + | - | + |
| Lane | A | B | C | D | E | F | G | H | I | J | K | L |
| Rel HA/rbcL | 0.30 | 0.24 | 1.00 | 0.02 | 0.27 | 0.26 | 1.00 | 0.05 | 0.15 | 0.12 | 1.00 | 0.01 |

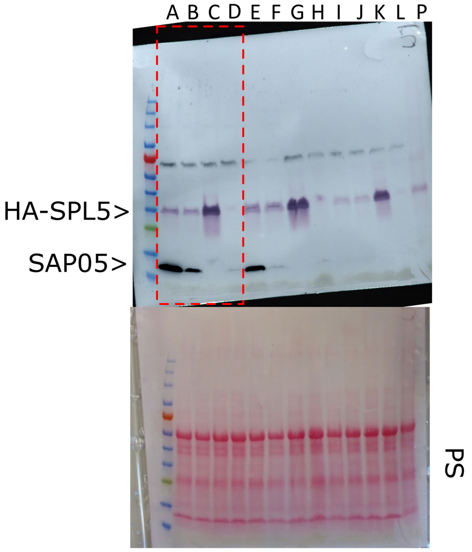

Supplement: Supplementary file 1 — Appendix 01 (PDF) [file pnas.2310664120.sapp.pdf]
